# Genome-Wide Analysis of the Emerging Infection with *Mycobacterium avium* Subspecies *paratuberculosis* in the Arabian Camels (*Camelus dromedarius*)

**DOI:** 10.1371/journal.pone.0031947

**Published:** 2012-02-29

**Authors:** Pallab Ghosh, Chungyi Hsu, Essam J. Alyamani, Maher M. Shehata, Musaad A. Al-Dubaib, Abdulmohsen Al-Naeem, Mahmoud Hashad, Osama M. Mahmoud, Khalid B. J. Alharbi, Khalid Al-Busadah, Abdulaziz M. Al-Swailem, Adel M. Talaat

**Affiliations:** 1 Department of Pathobiological Sciences, University of Wisconsin-Madison, Madison, Wisconsin, United States of America; 2 National Center for Biotechnology, King Abdulaziz City for Science and Technology, Riyadh, Saudi Arabia; 3 College of Agriculture and Veterinary Medicine, Qassim University, Qassim, Saudi Arabia; 4 College of Veterinary Medicine and Animal Resources, King Faisal University, Al-Hassa, Saudi Arabia; 5 Laboratory of Bacterial Genomics, University of Wisconsin-Madison, Madison, Wisconsin, United States of America; University of Louisville, United States of America

## Abstract

*Mycobacterium avium* subspecies *paratuberculosis* (*M. ap*) is the causative agent of paratuberculosis or Johne's disease (JD) in herbivores with potential involvement in cases of Crohn's disease in humans. JD is spread worldwide and is economically important for both beef and dairy industries. Generally, pathogenic ovine strains (*M. ap*-S) are mainly found in sheep while bovine strains (*M. ap*-C) infect other ruminants (e.g. cattle, goat, deer), as well as sheep. In an effort to characterize this emerging infection in dromedary/Arabian camels, we successfully cultured *M. ap* from several samples collected from infected camels suffering from chronic, intermittent diarrhea suggestive of JD. Gene-based typing of isolates indicated that all isolates belong to sheep lineage of strains of *M. ap* (*M. ap*-S), suggesting a putative transmission from infected sheep herds. Screening sheep and goat herds associated with camels identified the circulation of this type in sheep but not goats. The current genome-wide analysis recognizes these camel isolates as a sub-lineage of the sheep strain with a significant number of single nucleotide polymorphisms (SNPs) between sheep and camel isolates (∼1000 SNPs). Such polymorphism could represent geographical differences among isolates or host adaptation of *M. ap* during camel infection. To our knowledge, this is the first attempt to examine the genomic basis of this emerging infection in camels with implications on the evolution of this important pathogen. The sequenced genomes of *M. ap* isolates from camels will further assist our efforts to understand JD pathogenesis and the dynamic of disease transmission across animal species.

## Introduction

*Mycobacterium avium* subspecies *paratuberculosis* (*M. ap*), the causative agent of Johne's disease (JD) in ruminants, continues to be a significant economic drain on the livestock population throughout the world [1]. JD, also known as paratuberculosis, is widespread and infamously complex to control, especially in dairy operations. A recent study by The National Animal Health Monitoring System revealed that 68.1% of cattle dairy operations are infected with *M. ap* in the United States alone [2]. In Australia, a similar trend of *M. ap* infection has been observed among sheep flocks, causing an average loss of $60,500 due to mortalities in 2002 [3]. Once animals are infected with *M. ap*, the disease gradually advances towards its chronic form, which is characterized by granulomatous enteritis, progressive weight loss with diarrhea, and ultimately death [4]. Clinically infected animals can shed 10^6^–10^8^ CFU/g in fecal materials and as little as 10^3^ CFU/animal is sufficient to infect other animals through fecal oral route [5]. In addition, *M. ap* can survive in the environment for an extended periods of time [6], causing a significant risk to naïve animals from infected hosts. A number of domesticated ruminant species, e.g. cattle, buffalo, sheep, and goats are susceptible to *M. ap* infection with variable genotypes [7]–[9]. In camels (*Camelus dromedarius*), the nature of *M. ap* infection is uncharacterized, with increasing numbers of suspected cases in several regions of the world [10]–[12]. In this study we examined groups of camels suggested to contract *M. ap* infection to characterize the causative agent on the gene and genomic levels.

The epizoology of *M. ap* infection among a wide host range remains elusive and examining the interspecies transmission of JD could help in understanding the basis for such wide prevalence. Findings from earlier reports on experimental infection with *M. ap* from different host sources [13], [14] could not generalized to cross species natural infections under field conditions. Fortunately, several assays for genotyping *M. ap* were developed to examine the interspecies transmission of *M. ap* among domesticated animals [15], [16]. Genetic variations based on IS*900* [17], IS*1311* [15] and *gyrB* sequences [18] have revealed the presence of major groups of *M. ap* strains with preference to specific host source: the Type I/Type III or sheep strain (*M. ap*-S) and the Type II or cattle strain (*M. ap*-C). A recent report on genome-wide insertions and deletions further confirmed the presence of the two major lineages of *M. ap* [19]. Sheep strains of *M. ap*-S appear to have a substantial host preference for sheep with fastidious growth and the prevalence of yellow-orange colonies [9]. On the other hand, the *M. ap*-C strains infect a broad host range with more prevalence in cattle while most of the isolates show non-pigmented colonies [20]. The *M. ap*-C isolates are relatively easier to culture and faster to grow than sheep strains. However, the genetic basis of such host preferences for these variable strains is yet to be determined, especially in exotic animals. A few reports examined the development of JD in camels despite being probably the most notable member among the camelidae family. This situation, not surprisingly, is likely due to the failure to isolate the causative agent from the diseased camels.

Camels represent an important animal species in Saudi Arabia, where camel producers have suffered tremendously in recent years because of the annual increase of incidence of JD [11], [21]. The present study was undertaken to isolate and characterize the causative agent responsible for JD in dromedary camels (*Camelus dromedarius*) in Saudi Arabia. We were able to isolate *M. ap* from infected tissues, and genetic typing indicated these were *M. ap*-S strain. However, genome wide sequencing identified a significant number of SNPs between the camel isolates and the sequenced sheep strain. The results obtained provide interesting insights not only into the genome of *M. ap* infecting camels but also to the dynamic of disease transmission among animal species that share the same grazing fields.

## Results

### Johne's disease detection in *Camelus dromedarius*

Veterinary practitioners in north-east Saudi Arabia suspected the presence of JD in camel, sheep and goat herds based on clinical signs and serological testing [10], [11]. The local herd shepherds called this illness “Silag”, an Arabic terminology indicating profuse diarrhea. Our strategy for characterizing this infection in camels was based on collecting samples from animals suffering from chronic diarrhea suspected to have JD as well as from healthy animals. Our strategy also included the sampling of contact animals, mainly sheep and goat. During routine necropsy, sera and tissue samples (intestine, lymph node, liver) were collected. Additionally, fecal samples were collected from living animals with chronic diarrhea, mainly from the Al-Hassa region. While culturing results were pending, we used PCR to detect the presence of *M. ap* genomic DNA in collected samples. Surprisingly, tissues from all suspected cases (camels, sheep and goats) were positive for mycobacterial IS*900* (Fig. S1),16S rRNA and *hsp65* genes (Fig. S2) suggesting an active infection with *M. ap* in agreement with the clinical signs of JD, while none of the healthy animals tested positive for any of *M. ap* genes. In most cases, fecal samples were acid-fast stain and PCR positive, a further confirmation for shedding of *M. ap* bacilli from infected animals.

**Figure 1:**
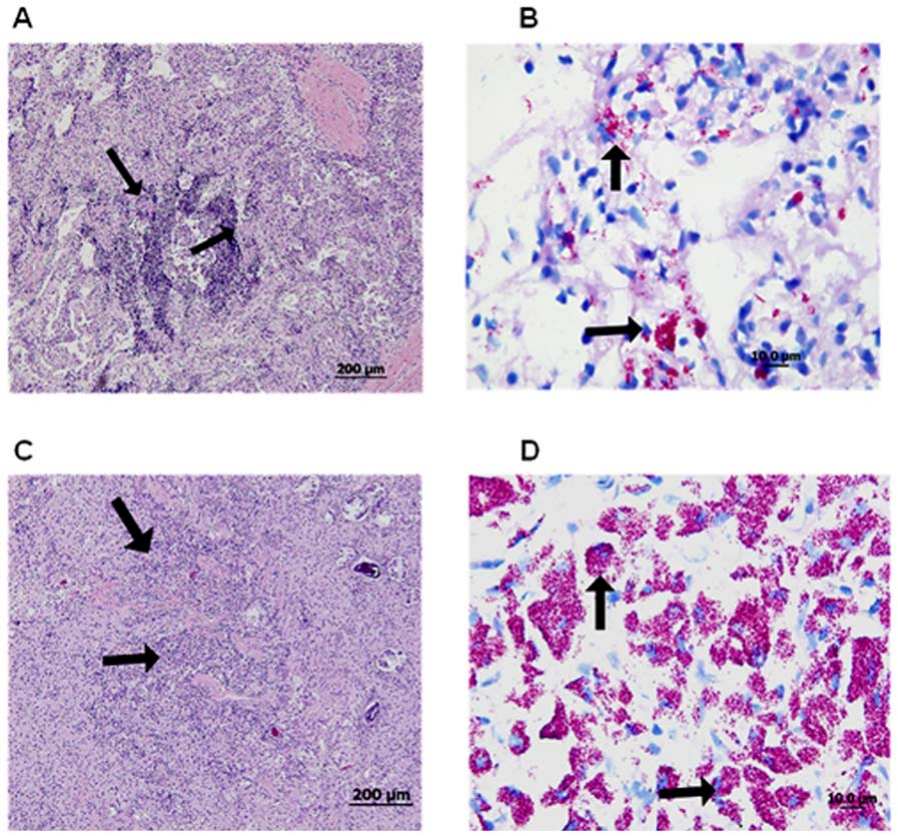
Histological analysis of camel samples collected from animals suffering from Johne's disease. A) A representative of lymph node thin section stained with H&E showing diffuse granulomatous response (arrows). B) A lymph node section stained with Zeil-Neelsen stain showing high level of acid-fast bacilli. C) A representative of intestinal section stained with H&E showing aggregates of lymphatic infiltration (arrows). D) An intestinal section stained with Zeil-Neelsen stain showing patches of acid-fast bacilli. Size bars are included in the bottom of each section.

In a second level of analysis, histopathology of sampled tissues revealed a high level of multi-bacillary form of *M. ap* infections (Fig. 1) in all infected organs (intestine, Lymph node and liver), another confirmation of the late stage of the disease in the examined camels. Examined sections showed diffuse granulomatous lesions (mainly of lymphocytic infiltrations, macrophages and a few giant cells) that were covered with patches of acid fast bacilli. Tissue sections from healthy animals did not show any granulomatous lesions or bacillary forms. When ELISA was performed, only 2 out of 6 serum samples were found to be positive for *M. ap* antigens. Unfortunately, the antibody ELISA has not been validated for the diagnosis of *M. ap* infection in camels and the sensitivity of this assay was based on testing in cattle. Overall, PCR and histopathology approaches indicated the presence of a multi-bacillary form of paratuberculosis in all suspected camel cases.

### Bacterial isolation from infected camels

Previous attempts to isolate *M. ap* from infected camels were unsuccessful [21], [22], likely due to the fastidious nature of this pathogen. In our hands, isolates of *M. ap* were cultured from 3 out of 39 clinical samples despite the extensive infection rate expected from PCR and histological analyses. Such disparity was likely caused by the heat-treatment step (72°C for 30 min) mandated by our shipping protocol that yielded most of the bacteria unculturable. However, colonies were observed from positive samples after 11–12 weeks of incubation and only on 7H10 medium (JQ5 camel Lymph node, Intestine and JQ6 camel liver) despite culturing on 7H9 broth, Lowenstein-Jenson and HEYM media. Suspected colonies were characterized by Ziehl-Neelsen (ZN) staining that revealed their acid resistance. Failure to grow both on 7H9 liquid broth and 7H10 plate without mycobactin J, an iron-chelating cell wall component, indicates that these isolates are strongly dependent on mycobactin, a distinctive feature of *M. ap* from the closely related *M. avium*. The comparative growth curve with *M. ap* K-10 (cattle strain), and *M. ap* S397 (sheep strain) indicated that the camel isolates were much slower to grow to that of the *M. ap* K10 but with similar rate to that of *M. ap* S397 (data not shown).

**Figure 2:**
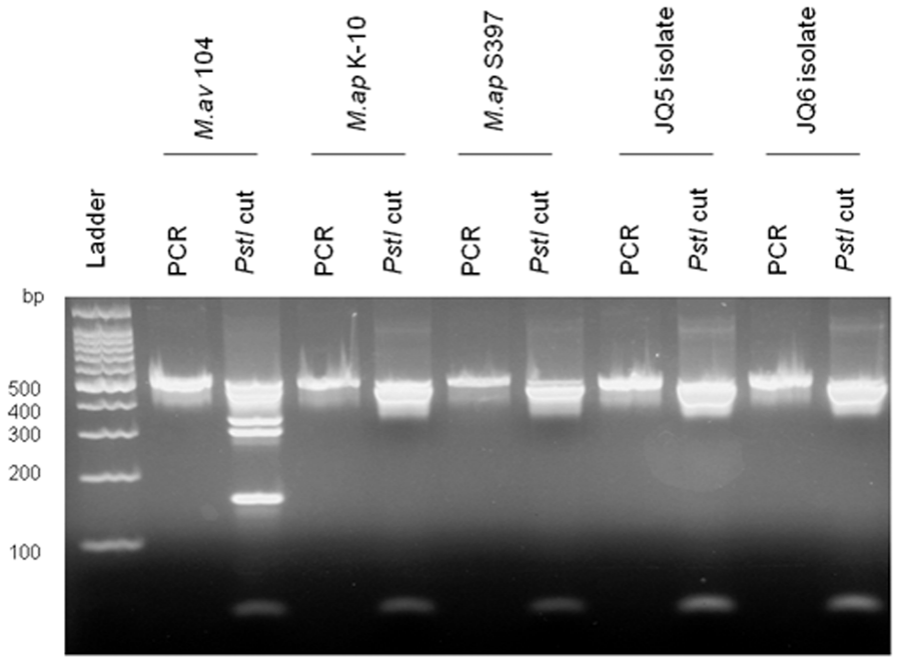
Species-level typing of mycobacterial isolates from camels. Ethidium bromide stained 2% agarose gel of PCR amplicons of the *hsp65* gene, following restriction enzyme analysis (REA) with *Pst*I. For each set, both undigested and digested products (second lane) are shown. A 100-bp Molecular size marker is shown in the first lane.

**Figure 3:**
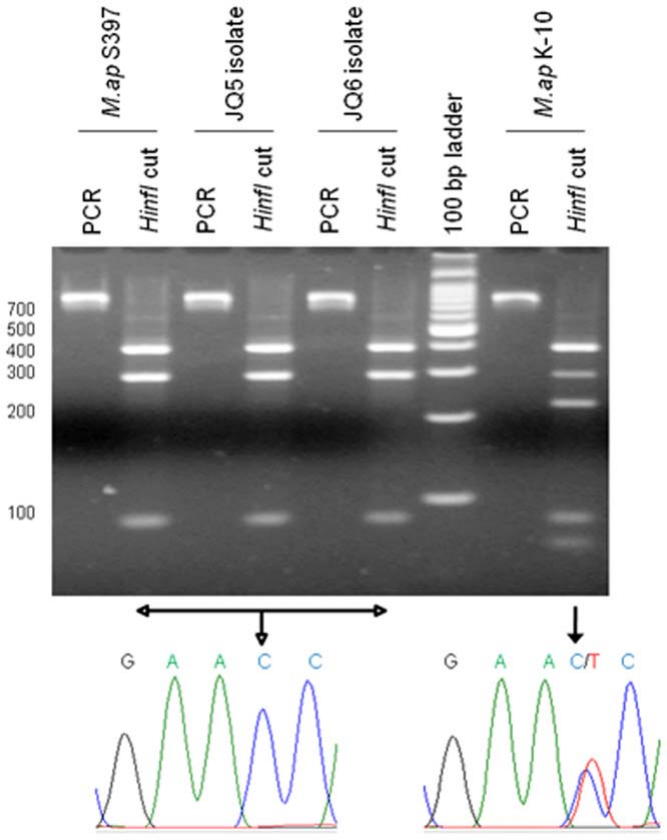
Genotyping of camel isolates. A) Ethidium bromide stained 3% agarose gel of PCR amplicons of the IS*1311* gene, following REA with *Hinf1*. For each set, both undigested and digested products (second lane) are shown. The PCR-REA analysis of IS*1311* shows *M. ap*-S (*M. ap* S397) and *M. ap*-C (*M. ap* K-10) strains in addition to camel isolates JQ5 and JQ6. A 100-bp Molecular size marker is included. B) DNA sequencing electropherogram showing the C/T nucleotide polymorphism surrounding base pair 223 in IS*1311*.

To confirm the identity of the grown colonies, we subjected isolates to a panel of PCR amplification and restriction enzyme analysis in comparison to other isolates with known host origin, namely, *M. ap* K-10 (cattle) [23], *M. avium* 104 (human) [24] and *M. ap* S397 (sheep) [25]. The target genes for analysis included *hsp65*, 16S rRNA, IS*900* and IS*1311*. For this analysis, a partial sequence (938 bp) of the 16S rRNA gene was amplified from all camel isolates, confirming their identity on the mycobacterial genus level [26]. Also, amplicons (517 bp) of the *hsp65* gene were obtained and subjected to *Pst*I digestion, identifying that these isolates as *M. ap* and not *M. avium* species [27] (Fig. 2). However, sequencing 5′ region of *hsp65* gene failed to discriminate camel isolates (*M. ap* JQ5 and *M. ap* JQ6) at strain level but when whole gene was analyzed, we were able to differentiate the camel clinical isolates from *M. ap* K-10 (Fig. S3). Another confirmation of *M. ap* identity came from the successful amplification of the insertion sequence IS*900* [28] that has 17 copies encoded in the *M. ap* genome [23]. To further characterize the new isolates, amplicons were obtained for the IS*1311* sequences and subjected to *Hinf*I restriction digestion (Fig. 3). The obtained pattern of digestion characterized that the JQ5 and JQ6 isolates are *M. ap*-S strains and not *M. ap*-C strains [15]. All of the obtained digestion patterns were further supported by Sanger sequencing of amplicons (Table 1) from each isolate and BLAST comparison to *M. ap* K-10. Overall, the genotyping protocols applied here strongly suggested the identification of a *M. ap*-S strains that caused this multi-bacillary form of JD in examined camels.

**Table 1:** IS*1311* polymorphisms in *M.av* and within *M.ap* strains.

Polymorphism position in IS*1311*sequence	*M. avium* GenBank: U16276	*M. avium ss. paratuberculosis*		
		*M. ap* K-10 from cattle	*M. ap* 397 from sheep	*M. ap* from camel
68	T	C	C	C
223	C	C or T	C	C
236	T	C	C	C
422	T	C	C	C
527	G	A	A	A
628	T	C	C	C

### Origin of the *M. ap* infection in camels

**Figure 4:**
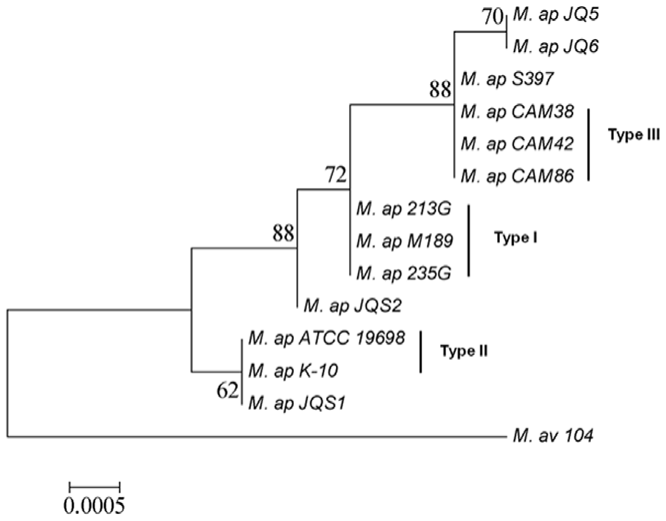
Phylogenetic analysis of *M. ap* isolates form different hosts. A dendrogram displaying the relationship between several *M. ap* isolates based on the sequence of 3 genes (*gyrA, gyrB* and *recF*). Isolates with JQ designation were analyzed in this study either from camels (JQ5, JQ6) or sheep (JQS1, JQS2). *M. ap* S397 is the standard strain for *M. ap*-S [25], *M. ap* K10 is the standard strain for *M. ap*–C [23] while the rest of the isolates were genotyped before [18]. All of the known genotyped are included in the figure. The bootstrap values (1000 replicates) are shown next to the branches. The tree is drawn to scale, with branch lengths in the same units as those of the evolutionary distances used to infer the phylogenetic tree. The evolutionary distances were computed using the Maximum Composite Likelihood method and are in the units of the number of base substitutions per site. See Table 2 for the sequence accession numbers used here.

In several regions of the world, animals are raised in mixed populations with other animal species or even with access to wildlife animals. In searching for the origin of *M. ap* infection in camels, samples were collected from animals co-mixed with camels to determine the possible source of camel infection. Interestingly, PCR-restriction enzyme analysis of IS*1311* from sheep and goat infected samples identified the presence of mainly *M. ap*-C strains in the examined goats. However, both Types *M. ap*-S (*M. ap* JQS2) and *M. ap*-C (*M. ap* JQS1) strains were identified in sheep samples (Fig. S4). To genotype sheep and goat isolates from the same region of Saudi Arabia, we used multi-locus sequence analysis (MLSA) approach that exploits sequence level variability in house-keeping genes conserved in a broad range of microorganisms [29]. We focused our analysis on 3 house-keeping genes (*gyrA, gyrB*, and *recF*) that are polymorphic between *M. ap*-S strains (Type I and Type III) and *M. ap*-C (Type II) strains [18], [30]. There were a total of 9 polymorphic sites between the sheep isolates (*M. ap* JQS1and *M. ap* JQS2) that distinguished them from camel isolates (Fig. 4). The sequence between *M. ap* JQS1 and Type II strains was least variable whereas, *M. ap* JQS2 was grouped with sheep strains, an indication of potential sheep source for camel infections. Further, camel isolates were variable at three sites (one SNP in each *gyrA*, *gyrB*, and *recF* gene) when compared to Type I strains. Such variations suggest the inclusion of camel isolates in type III group, a sub-lineage of sheep isolates identified before [31]. Interestingly within these 3 loci, we found a single variable site in *gyrB* gene that distinguished camel isolates (*M. ap* JQ5 and *M. ap* JQ6) from Type III strains. This site represents camel strain of *M. ap* specific SNP and could be employed in future comparative epidemiological studies of Johne's disease.

To evaluate the nature of evolutionary pressure on the camel isolates, we examined the dN/dS ratio for SNPs in each examined strain. Sequences from 11 house-keeping genes (*gyrA*, *gyrB*, *dnaA*, *dnaK*, *aspB*, *gnd1*, *hsp65*, *groEL1*, *pepB*, *recF*, and *sodA*) were combined and concatenated to generate a 17,190-bp in-frame semantide for each strain included in the study. In total, 175 polymorphic sites (1%) were found within these variable alleles leading to the separation of these strains into individual branches (Fig. S5). In addition, there were 24 SNPs that could be used to distinguish *M. ap* K-10 from sheep strains of *M. ap* (Table S1). In comparison to *M. av* 104, the average dN/dS ratio for all *M. ap* subtypes was ∼0.07, an indication to the highly conserved nature of the selected sequences among members of the *M. avium* complex. In contrast, out of the 24 SNPs that segregated *M. ap*-S and *M. ap*-C strains, 12 (50%) were non-synonymous (nSNPs). The average pairwise dN/dS ratio for both camel isolates and the standard *M. ap* S397 was increased to 0.5, comparable to that previously observed [30] when *M. ap*-S sequences were compared against *M. ap* K-10 (*M. ap*-C). Such analysis indicated the diversifying selective pressure of camel isolates compared to cattle isolates.

### Whole genome analysis of camel isolates

Although gene-based approaches grouped the camel isolates with *M. ap*-S strains, they are not enough to get insights into the unidentified genomic changes (e.g. indels or SNPs) at the whole genome level in these camel isolates. Such genomic rearrangements [24] could play a role in disease surveillance and control [32] as well as provide a better understanding of the evolution of this important pathogen. In an effort to resolve the whole genome of *M. ap* isolated from camels, we used Next-generation sequencing technology (Illumina, HiSeq 2000) to generate a draft sequence of two camel isolates (*M. ap* JQ5, and *M. ap* JQ6; see Table 2 for accession numbers). A total of 22,685,456 and 14,118,565 read counts (for *M. ap* JQ5 and *M. ap* JQ6, respectively) were mapped against a reference genome from a sheep isolate (*M. ap* S397) (see Table 2 for accession number) sequenced by our group. An average coverage of 406× and 240× across the genome for *M. ap* JQ5 and *M. ap* JQ6 isolates, respectively, was achieved. Sequence statistics revealed that the camel isolate *M. ap* JQ5 has a G+C content of 69.3% and a genome size of 4,735,471 bp with 98.3% complete coverage of the genome when compared to the *M. ap* S397 genome. More comparative genomic features of the camel isolates relative to the genomes of isolates from sheep and cattle are detailed in Table 3.

**Table 2:** A list of accession numbers for organisms and genes used in this manuscript.

Organisms or genes	Accession number
*M. avium* 104	NC_008595
*M. ap* S397	AFIF00000000
*M. ap* K-10	SRR060191
*M. ap* JQ5	AHAZ00000000
*M. ap* JQ6	AHBA00000000
*M. intracellulare* ATCC 13950	ABIN00000000
*gyrA* for Type I *M. ap*	EU029115
*gyrA* for Type III *M. ap*	EU029113
*gyrB* for Type I *M. ap*	EU029112
*gyrB* for Type III *M. ap*	EU129114
*recF* for Type I *M. ap*	EU409980
*recF* for Type III *M. ap*	EU409982

**Table 3:** Summary of next generation sequencing results.

Parameters	JQ5 isolate	JQ6 isolate
Reference organism	*M. ap* S397	*M. ap* S397
Reference length (bp)	4,813,711	4,813,711
Consensus length (bp)	4,735,471	4,697,010
% G+C	69.3	69.3
% Homology to *M. ap* S397	98.3	97.6
% Homology to *M. ap* K-10	97	97
Average Coverage	406	240
Mean mapped read length	82.7	80.3
Mean paired read distance	300	335
No. of indels against *M. ap* S397	89	69
No. of SNPs against *M. ap* S397	1026	929
No. of indels against *M. ap* K-10	269	244
No. of SNPs against *M. ap* K-10	3439	3245

### Evolutionary history of the camel isolates

**Figure 5:**
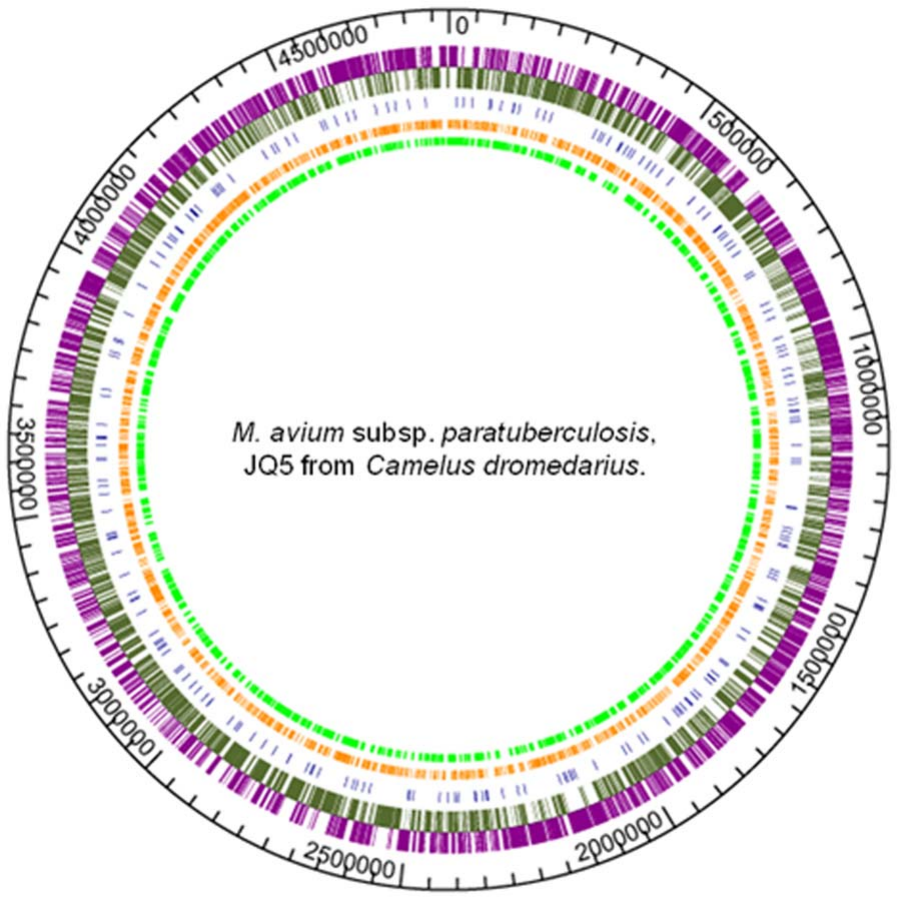
Circular map of the newly identified SNPs and indels in camel isolate JQ5 relative to the *M. ap* K-10 strain [**51**]. The outer circle shows the genomic scale. The second circle shows the location of the 4,395 ORFs in the *M. ap* K-10 genome. Genes (magenta) on the forward strand are shown outside of the baseline; genes (olivegreen) on the reverse strand are shown inside of the baseline. Inner circles show all indels (blue), synonymous SNPs (orange), and nonsynonymous SNPs (lime) identified in *M. ap* of camel origin. The figure was generated with GenVision software (DNAStar, Madison, WI).

One goal of our study was to understand the mechanisms of the emergence of JD among camels. To get a better understanding of the diversity among *M. ap* isolates circulating in independent but mixed animal populations, we compared the genome sequence of camel isolates to *M. ap* S397 (*M. ap*-S strain) as well as the genome of *M. ap* K-10 (*M. ap*-C strain) (see Table 2 for accession number). Genome-wide comparative analysis identified single nucleotide polymorphisms (SNPs) and indels in *M. ap* isolates from camels compared to *M. ap* K-10 by 3439 and 3245 SNPs in *M. ap* JQ5 and *M. ap* JQ6, respectively (Fig. 5). Also, small indels (1–8 bp) were identified (269 to 244 for *M. ap* JQ5 and *M. ap* JQ6, respectively). Interestingly, when the genome of JQ6 compared to that of JQ5, small differences in SNPs (N = 99) were found, suggesting a high sequence conservation in *M. ap* isolated from different camels. However, all phylogenetic analyses using house-keeping genes were not able to distinguish both JQ5 and JQ6 isolates. The complete list of SNPs and indels are available in Tables S2 and S3.

Further inspection of the SNPs in camel isolates indicated that the average number of SNPs was 1 per 1589 bp to 1 per 5288 bp in camel isolate when compared to *M. ap* K-10 and *M. ap* S397, respectively, another indication of the close relationship between camel and sheep isolates. The identified SNPs were classified into four different categories: trasitions, synonymous SNPs, non-synonymous SNPs, and intergenic SNPs (Fig. S6). In both camel isolates, the percentage of intergenic SNPs was around 14%. Interestingly, the majority of the SNPs (86%) in the coding region were non-synonymous (54%) suggesting a positive selective pressure on these organisms. The percentage of SNP transition was 64%, indicating a substitution bias in favor of nucleotide substitution within the purine or pyrimidine group. On the other hand, when the genome of the sheep strain, *M. ap* S397 was compared, the average numbers of SNPs (N = 1036 for JQ5 and 929 for JQ6 isolate) and indels were significantly less than when the *M. ap* K-10 strain was compared. Moreover, when we compared camel isolate JQ5 and *M. ap* S397 against *M. ap* K-10, a small difference in SNPs was observed between sheep strain and camel isolate (Fig. S7). Finally, our analysis indicated majority of the SNPs were synonymous (lower selective pressure) in the genome of camel isolates when compare to the *M. ap* S397 strain, suggesting a recent divergence from a common ancestor. Overall, whole-genome analysis confirmed the identified genotypes based on selected house-keeping genes.

To examine the impact of genomic changes on pathobiology of *M. ap* in camel JD, we analyzed the presence of SNPs on coding sequences. Compared to *M ap* K-10, SNPs were detected in a 1860 gene-coding proteins in the camel isolates (Fig. S8). Most of these genes harbored one to six SNPs with single point mutations being the most common. In certain genes, there were higher numbers of SNPs per gene relative to others (Table 4). Other polymorphic genes included those coding for antigenic proteins (e.g. *pstA*), mammalian cell entry proteins (e.g. *mceA1-2*), PE/PPE family proteins, and mycobactin biosynthesis (e.g. *mbtB-mbtH*). These genes are suggested to be involved in JD pathogenesis and detection of SNPs in their coding sequences suggests a selective pressure for adaptation to the camels. Because of the importance of *mce* gene family, we further analyzed some of its members (*mceA2*, *mce1B*, *mce2*, *mce3*, *mce4*, and *mce1F*) by pairwise alignment that confirmed the close relationship between the camel isolates with *M. ap*-S strain (Fig. S9). Unfortunately, we did not have the full gene sequence of the *mce1A*, a member of the *mce* gene family, to be included in such comparison. Finally, our analysis revealed a large number of SNPs in the mycobactin biosynthesis gene cluster (e.g. *mbtB-mbtH*), providing a possible explanation of delay in growth of the camel isolates in synthetic media.

**Table 4:** List of genes with high number of SNPs in *M. ap* JQ5 compared to *M. ap* K-10.

Gene description	Size of the gene (bp)	Number of SNPs
“MAPK_2348”	19,155	55
“MAPK_0436”	1,389	17
“MAPK_2538”	1,221	17
“MAPK_0308”	1,386	15
“MAPK_1273”	1,419	14
*pks12*	12,513	14
*kasB_2*	1,260	13
“MAPK_2303”	1,455	10
“MAPK_1538”	11,040	10
“MAPK_4243”	3,645	8
*fas*	9,279	8
*mbtE*	5,280	8
“MAPK_0028”	9,207	7
“MAPK_1689”	9,156	7
*pks8*	3,288	7

## Discussion

Several reports suggested the emerging infection with *M. ap* in camels (*Camelus dromedarius*) raised in the Arabian Peninsula [11], [12]. However, the molecular basis of this infection remained elusive likely due to the unsuccessful attempts to culture the organism from the diseased camels. Camels are an economically important species in South Asia, Middle East, and North Africa, where they are utilized for transportation, sport, and the production of meat and milk. These attributes have generated our interest in investigating the causative agent of JD in camel. An important aspect of *M. ap* infection in camels demonstrated in this study was the prevalence of the multi-bacillary form observed in examined tissues. Although we focused our sampling strategy on diseased animals for bacterial isolation, we were surprised that all infected animals showed a large number of granulomas with patches of mycobacterial bacilli. This lepromatous form of JD has also been reported in sheep before [4], [9] but the extent of *M. ap* induced lesion was much higher in the examined camels. The nature of the infection transmission was further analyzed when majority of examined fecal samples were positive for the presence of *M. ap*. Such analysis also suggested that most of the examined animals were at the late stage of JD, another reason for the observed multi-bacillary form. Analysis of more animals with less severe clinical signs could shed the light on the pathogenesis of JD in camels.

We successfully isolated *M. ap* from only two diseased camels (JQ5 and JQ6) despite the detection of *M. ap* in all diseased animals. This low isolation rate could be attributed to the necessary heat treatment protocol for all tissues collected from the sampling location to inactivate potential sample contamination with Foot-Mouth Disease. Additionally, the isolates were fastidious and slow to grow, in contrast to the *M. ap*-C strain. Similar observations were noticed previously for the isolation of sheep strains [33], [34]. Unfortunately, because of the low success rate of ELISA detection from camel samples, we were not able to count on serological testing for the diagnosis of JD. Recently, studies showed loss of function and capacity of the immune cells from the multi-bacillary form of the disease [35]. It is also possible that ELISA based on camel isolates will perform better than those based on the cattle strains.

In this study, we took advantage of molecular typing protocols to decipher the origin of the *M. ap* infection. Based on our MLSA analysis in several conserved genes, we found that isolates sequenced in this study (*M. ap* JQ5 and *M. ap* JQ6) are representative of the *M. ap*-S and closely related to *M. ap* JQS2 cultured from sheep raised in the same region. However, given the fact that there are variable sheep subtypes of *M. ap*, more sheep and goat samples need to be analyzed to confirm this association. Interestingly, three SNPs (one each in *gyrA, gyrB* and *recF* gene) were able to classify JQ5 and JQ6 isolates as Type III strains, a sub-lineage of the sheep strains of *M. ap* [31]. Nonetheless, we opted to utilize the *M. ap*-S characterization of the camel isolates because of the few numbers of SNPs used to designate the Type III strains. More importantly, the lesions associated with type III strains could not be delineated from those associated with typical *M. ap*-S strains. Clearly, full genome sequencing of representatives of Type III strains and experimental animal infections with those strains will increase the confidence in the designation of Type III strains as an independent group instead of being a divergent member of the sheep strain.

Sequencing the genome of camel isolates identified additional genome-wide features that could not be gleaned from specific gene analyses. Comparative genome analysis of the camel isolates against a standard cattle isolate (*M. ap*-C), revealed a significant difference in SNPs and indels which were much less when compared to the sheep isolate (*M. ap*-S). Interestingly, members of the *mce* gene family from *M. ap* (e.g. *mce1A*) was shown before to be highly similar to its counterpart in *M. avium* subspecies *hominissuis* 104 suggesting an *M. avium* origin [36]. However, in our hands, most of this gene family (*mceA2, mce1B,..etc*) were closely related to the *M. ap*-S strain, suggesting a potential origin of this gene family from *M. ap*-S isolates (and not *M.* avium) circulating in sheep. Unfortunately, the full gene sequence for *mce1A* was not obtained for comparative analysis. It is also possible that the *mce1A* is independently evolved from the rest of the gene family. Taken together, our analysis indicated that camels were infected with a member of the *M. ap*-S cluster, most likely originating from sheep despite the presence of *M. ap*-C isolates circulating in goats and sheep raised in the same geographical location. Based on our MLSA and genome-wide comparative analysis, camel isolates could represent a distinctive sub-group of *M. ap*-S, an idea remains to be investigated with a larger number of camel isolates.

In *M. tuberculosis*, a possible mechanism of antigenic drift has been proposed due to the mutations within the immune-dominant epitopes of secretary antigenic protein [37]. Members of the *mce* gene family, PE and PPE families have been found to be immunogenic and are implicated in virulence [38]–[40]. Moreover, mutations in the *mce* operons have been suggested to alter the functional changes in the biology of *M. tuberculosis* [41]. The high number of SNPs observed in the antigenic proteins encoded in the camel isolates included *mce*, and PE/PPE family proteins could be the result of host adaptation during camel infection. Whether these differences are due to adaptive evolution or specific geographic distribution of *M. ap* genotypes is the subject of ongoing investigation. Overall, our study successfully genotyped isolates causing Johne's disease in camels and provided a better understanding of the disease transmission among animals under the desert conditions of the Arabian Peninsula.

## Materials and Methods

### Animals and study site

A total of 45 clinical samples were tested from 20 different camels (*Camelus dromedarius*) suspected of having JD with signs of chronic diarrhea. Of these specimens there were 24 tissue samples (intestine, lymph node and liver), 15 fecal samples and 6 serum samples. We also analyzed a few samples from sheep and goats (N = 5) showing signs of JD (chronic diarrhea, emaciation). Camels were raised in the Arabian Desert, north east region of Saudi Arabia (Qassim and Al-Hassa), in small-sized but mixed flocks with other animals, mainly sheep and goats. Camels suffering from chronic diarrhoea and severe weight loss were anaesthetized by intramuscular injection of xylazine (0.35 mg/kg) and ketamine (6 mg/kg). Animals were bled by severing the major vessels of the neck. Euthanized camels were undergone necropsy for post-mortem changes and sample collection. All serum, tissue and fecal samples were collected by teams of researchers in Saudi Arabia and were heat-treated at 72°C for 30 min before transportation to the Laboratory of Bacterial Genomics, University of Wisconsin-Madison, USA, where they were kept at -80°C until further sample processing. The heat treatment was necessary to inactivate foreign pathogens as mandated by our import license issued by the APHIS services, USA.

### Ethics statement

All procedures for animal euthanasia and sample collection were carried out at the veterinary teaching hospital, Qassim University according to the approved animal protocol (QU#01100) from the Qassim University Committee for Rules of Experimentation on Live Animals. Anaesthesia and euthanasia of animals were carried out in strict accordance with the recommendations by the Code of Practice for camels in Western Australia and others [42]–[44].

### Sample preparation and culturing

Tissue samples were placed in 10 ml PBS containing 5% oxalic acid in order to remove any contaminating organisms and homogenized using a Tissue-Tearor (BioSpec Products, Inc., Bartlesville, OK) with a 7 mm probe diameter at 20,000 rpm within a BSL-II cabinet. The homogenates were centrifuged (4,000 rpm) at room temperature for 10 minutes and the resulting pellets were washed twice with PBS before final resuspension in 10 ml of warm 7H9 broth (Difco, Sparks, MD) supplemented with 0.5% glycerol, 2 µg/ml mycobactin J (Allied Monitor, Fayette, MO), and 10% ADC (2% glucose, 5% bovine serum albumin fraction V, and 0.85% NaCl) [45]. All homogenates were plated on each of the following solid media: Lowenstein-Jensen (LJ) medium (BD, Franklin Lakes, NJ), Herold egg yolk medium (HEYM; BD, Franklin Lakes, NJ), or Middlebrook 7H10 agar (BD) supplemented with 0.5% glycerol, 2 µg/ml mycobactin J, and 10% ADC and plates were incubated at 37°C for 12 weeks. To improve recovery rate, homogenates were also inoculated onto the Middlebrook 7H9 broth supplemented with 0.5% glycerol, 2 µg/ml mycobactin J, 0.05% Tween 80, and 10% ADC at 37°C with shaking at 115 rpm/min. Fecal samples were decontaminated according to the protocol described before [46]. One gram of each fecal sample was treated with 10 ml of 0.9% hexadecyl pyridinium chloride (HPC) overnight (16 hr) to remove fungal and non-mycobacterial contaminants. Supernatants were centrifuged at 4,000 rpm for 15 min to harvest mycobacterial pellets. Pellets were washed with PBS and resuspended in 10 ml of warm 7H9 broth following inoculation on several medium described earlier.

### Histopathology and serological analysis

All organ specimens were preserved in 10% neutral buffered formalin (NBF) before tissues embedded in paraffin and prepared for microtome sectioning (3 to 5 µm). Sections were stained with hematoxylin and eosin staining or Ziehl–Neelsen staining for microscopic evaluation as described previously [47]. Stained slides were examined carefully to score changes in the inflammatory response using a scale ranging from 0 to 5. A score of 1 is indicative of restricted inflammatory response, while a score of 5 represented multiple granulomas in more than three representative microscopic fields. All camel serum samples were sent to the Johne's testing center, Madison, WI for the detection of antibodies against *M. ap* using the Parachek ELISA kit as per manufacturer's directions (Biocor Animal Health Inc. Omaha, NE).

### Isolation of genomic DNA from cultures

To obtain high quality genomic DNA (gDNA), mycobacterial cultures at OD_600_ = 1.5 were pelleted, washed and resuspended in equal volume of TE buffer. The bacterial suspension was placed at 80°C for 20 min to kill all the mycobacteria [26]. Tubes were allowed to cool to room temperature and 10 ul of 100 mg/ml lysozyme was added to each tubes followed by incubation at 37°C for 3 hr with occasional mixing. A solution of SDS (10%) and proteinase K (20 mg/ml) was added to each tube in a ratio of 88∶12. Tubes were incubated at 65°C for 2 hrs followed by addition of 100 µl of 5 M NaCl at 65°C for additional 10 min. Solution of 10% Cetyl trimethyl ammonium bromide (CTAB) (Sigma-Aldrich, St. Louis, MO) (80 µl) was added, mixed and incubated at 65°C for another 10 min. DNA was then extracted with an equal volume of phenol/chloroform/isoamylalcohol (25∶24∶1 v/v/v) and similarly with chloroform/isoamylalcohol (24∶1 v/v) followed by precipitation with 0.6 volumes of ice cold isopropanol [26]. The DNA was pelleted by centrifugation (10,000 rpm) at 4°C for 15 min and pellets were washed with cold 75% ethanol and dried in Speed-Vac for 5 min. The DNA was finally resuspended in 50 µl sterile distilled water. Quality of the gDNA was verified by both NanoDrop (Thermo Scientific, Wilmington, DE) machine and electrophoresis. This gDNA was used for PCR and subsequent reactions.

### Polymerase chain reaction (PCR) and Sanger DNA sequencing

PCR reactions 25 µl, containing 1 M betaine, 50 mM potassium glutamate, 10 mM Tris-HCl pH 8.8, 0.1% Triton X-100, 2 mM magnesium chloride, 0.2 mM dNTPs, 0.5 µM each primer, 0.5 U of Taq DNA polymerase (Promega) and 25 ng of genomic DNA. In some cases, 5 µl of tissue homogenates were directly used as a template. The amplification thermocycle was subjected to an initial denaturation step of 94°C for 5 min followed by 35 cycles of denaturation at 94°C for 30 s, annealing at 55°C for 30 s and extension at 72°C for 1 min, and followed by a final extension at 72°C for 7 min [26]. Polymerase chain reaction amplicons were evaluated by electrophoresis in 2% agarose gels prestained with ethidium bromide (0.5 µg/ml). The interested single-band products from PCR reactions were purified and extracted using gel extraction kit (Promega). A total of 250–300 ng of DNA and 1 U of the restriction enzymes *Hinf*I or *Pst*I (NEB, MA, USA) were used in the digestion reactions according to the manufacturer's recommendations. Digestion reactions were assessed using 2–3% agarose gels stained with ethidium bromide. Following amplication, purified PCR fragments for genotyping and MLSA analysis were sequenced with BigDye Terminator v3.1 (Applied Biosystems, Foster City, CA) with primers listed in Table 5, according to the manufacturer's instruction.

**Table 5:** Primers used in this study.

Primer	Gene and direction	Purpose	Sequence
1	IS*900*, forward	PCR, Sequencing	TACCTTTCTTGAAGGGTGTTCGGGG
2	IS*900*, reverse	PCR, Sequencing	TTGTGCCACAACCACCTCCG
3	IS*1311*, forward	PCR-REA, Sequencing	GCGTGAGGCTCTGTGGTGAA
4	IS*1311*, reverse	PCR-REA, Sequencing	TCAGAGATCACCAGCTGCAC
5	*hsp65*, forward	PCR-REA, Sequencing	GAGGGCGTCATCACCGTCGAGG
6	*hsp65*, reverse	PCR-REA, Sequencing	CGGCGATGGCGTCGGAGTCACC
7	*gyrA*, forward	PCR, Sequencing	ACGTCGTCGTCACCATCAC
8	*gyrA*, reverse	PCR, Sequencing	CCTCACCCAGATTCATCAGC
9	*gyrB* (region 1), forward	PCR, Sequencing	AAGAAGGCGCAAGACGAATA
10	*gyrB* (region 1), reverse	PCR, Sequencing	AGCTTCTTGTCCTTGGCGTA
11	*gyrB* (region 2), forward	PCR, Sequencing	GTACGCCAAGGACAAGAAGC
12	*gyrB* (region 2), reverse	PCR, Sequencing	GTGGGATCCATTGTGGTTTC
13	*recF*, forward	PCR, Sequencing	GGGAAGACGAATCTGCTTGA
14	*recF*, reverse	PCR, Sequencing	GGACTGGTCGTCGCTCAT

### Phylogenetic analysis

All sequences were analyzed with BLASTn algorithm on the NCBI web portal http://blast.ncbi.nlm.nih.gov/Blast.cgi. For genotyping camel isolates, we concatenated sequences from 11 genes (*dnaA, dnaK, hsp65, aspB, gnd1, groEL1, gyrA, gyrB, pepB, recF, and sodA*), generating a 17.190-bp semantide. A rooted tree was computed using MEGA [48] with *M. intracellulare* ATCC 13950 (see Table 2 for accession number) serving as the out-group. Distances were calculated using the neighbor-joining (p-distance) method by pairwise analysis with gaps and performing 1,000 bootstraps replicates. Analyses for Non-synonymous (dN) to synonymous (dS) polymorphisms (dN/dS) [49] were computed for each *M. ap* strains against *M. avium* 104 using the Nei-Gojobori method with Jukes-Cantor correction [48], [50]. MLSA approach was also employed to check relatedness of these camel isolates with *M. ap* strains identified in the sheep herds from the same region of Saudi Arabia. For this, polymorphic regions distinguishing *M. ap*-S from *M. ap*-C were amplified from *gyrA*, *gyrB*, and *recF* loci with primers used before [18], [30]. The accession numbers for genes used in this manuscript are listed in Table 2.

### Next-generation whole genome sequencing

Massive parallel sequencing using the Solexa-technology (Illumina) was performed at the UW-Madison Biotech Center sequencing facility. Whole-genome was sequenced using the Illumina HiSeq 2000 platform using one flow cell lane with 100-cycle paired end chemistry (www.illumina.com). Assembly and analysis of sequencing data were performed with the CLC Genomics Workbench 4.6 (http://www.clcbio.com/). Read mapping of these camel isolates (JQ5 and JQ6) was performed against reference sheep strain (*M. ap* S397) (see Table 2 for accession number). Assemblies were performed for each camel isolate individually and later combined together to get a better and deeper coverage of the genome. Single nucleotide polymorphisms (SNPs) and indels (insertion and deletion polymorphisms) were performed against *M. ap* S397 and *M. ap* K-10 (cattle strain) (see Table 2 for accession number) using CLC Genomics Workbench 4.6. To reduce false calls, SNP analysis parameters were set as follows: average base quality filter cutoff 15, central base quality filter cutoff 20, minimum sequence coverage 10, minimum variation frequency cutoff 50%, and maximum variation 2. For the indel analysis, minimum sequence coverage was set to 10 and minimum variation frequency cutoff was 50%. The sequences for the Whole Genome Shotgun project have been deposited at DDBJ/EMBL/GenBank under the accession AHAZ00000000 for *M. ap* JQ5 and AHBA00000000 for *M. ap* JQ6. The versions described in this paper are the first version.

## Supporting Information

Figure S1 **Confirmation of** ***M. ap*** **isolates identity using PCR.** Amplification of *IS*900 fragments from selected clinical samples. PCR reactions show 241 bp band from the infected tissue samples (A) (Lanes: 4–9) and fecal samples (B) (Lanes: 12–18). Origins of the clinical samples along with the example animal identification are listed at the top of the gel. A 100-bp Molecular size marker is included in lanes: 1 and 10. PCR controls are included in lanes: 2, 3, 11 and 12.
(TIF)

Figure S2 **Species-level differentiation of mycobacterial isolates from camels using** ***hsp65*** **and 16S rRNA gene targets.** Ethidium bromide stained agarose gel of PCR reactions show 517 bp band for *hsp65* (A) (Lanes: 2–6) and 938 bp band for 16S rRNA (B) (Lane 8 and 9). Top of the gel shows origin of the clinical samples along with the animal identification.
(TIF)

Figure S3 **Phylogenetic analysis of camel isolates based on whole** ***hsp65*** **gene.** Camel isolates were distinguished as *M. ap*-S strain when whole *hsp65* gene was analyzed. A total of 4 polymorphic sites, shared with *M. ap* S397, was able to differentiate these clinical isolates from *M. ap* K-10. The bootstrap value (1000 replicates) is shown next to the branch. The tree is drawn to scale, with branch lengths in the same units as those of the evolutionary distances used to infer the phylogenetic tree.
(TIF)

Figure S4 **PCR-REA of IS** ***1311*** **sequence from sheep and goat infected samples.** Ethidium bromide stained 3% agarose gel of PCR amplicons of the IS*1311* gene, following REA with *Hinf1*. For each set, both undigested and digested products (second lane) are shown. The PCR-REA analysis of IS*1311* shows presence of mainly *M. ap*-C strains in the examined goats (JQG1, JQG2, and JQG3) but both *M. ap*-S and *M. ap*-C strains are detected in sheep samples (JGS2 and JQS1, respectively). A 100-bp Molecular size marker is shown in the first lane.
(TIF)

Figure S5 **Phylogenetic analysis of house-keeping genes from** ***M. ap*** **strains analyzed in this study.** The dendrogram was constructed based on 11 genes (*gyrA*, *gyrB*, *dnaA*, *dnaK*, *aspB*, *gnd1*, *hsp65*, *groEL1*, *pepB*, *recF*, and *sodA*) using the Neighbor-Joining method and *M. intracellulare* as an out-group. In total, 175 polymorphic sites (1%) are found within these variable alleles leading to the separation of these strains into individual branches. A single variable site in *gyrB* gene distinguishes camel isolates (*M. ap* JQ5 and *M. ap* JQ6) from *M. ap* S397. The percentage of replicate trees in which the associated taxa clustered together in the bootstrap test (1000 replicates) is shown next to the branches. The tree is drawn to scale, with branch lengths in the same units as those of the evolutionary distances used to infer the phylogenetic tree. The evolutionary distances were computed using the Maximum Composite Likelihood method (pairwise deletion option) and are in the units of the number of base substitutions per site.
(TIF)

Figure S6 **Summary of single nucleotide polymorphisms in** ***M. ap*** **isolates from camel compared to** ***M. ap*** **K-10 strain.** The number in parentheses represents the total number of SNPs in each sequenced isolate. The color bars show the percentage distributions of different SNP categories in each isolate.
(TIF)

Figure S7 **Venn diagram of single nucleotide polymorphisms in sheep strain and camel isolate, JQ5 compared to** ***M. ap*** **K-10.** SNP analysis was performed using MAUVE algorithm [52].
(TIF)

Figure S8 **Distribution of number of SNPs per gene.** Note the large number of genes with single nucleotide polymorphism.
(TIF)

Figure S9 **Phylogenetic analysis of** ***mce*** **genes.** The phylogenetic tree displaying close relationship between the camel isolates with *M. ap*-S strain is based on members of *mce* genes (*mceA2*, *mce1B*, *mce2*, *mce3*, *mce4*, and *mce1F*). The percentage bootstrap values (1000 replicates) are shown next to the branches. The tree is drawn to scale, with branch lengths in the same units as those of the evolutionary distances used to infer the phylogenetic tree. The evolutionary distances were computed using the Maximum Composite Likelihood method using pairwise deletion option.
(TIF)

Table S1 List of polymorphic sites in 11 house-keeping genes from various *M. ap* subtypes.
(DOC)

Table S2 Complete list of SNPs in *M. ap* JQ5 and *M. ap* JQ6 isolates compared to *M. ap* K-10.
(XLSX)

Table S3 Complete list of indels in *M. ap* JQ5 and *M. ap* JQ6 isolates compared to *M. ap* K-10.
(XLS)
