# Supplementary figures and images for: Genome-Wide Analysis of the Emerging Infection with Mycobacterium avium Subspecies paratuberculosis in the Arabian Camels (Camelus dromedarius)
Source: PLoS One. 2012 Feb 29;7(2):e31947. doi: 10.1371/journal.pone.0031947 (PMC3290536; doi:10.1371/journal.pone.0031947)

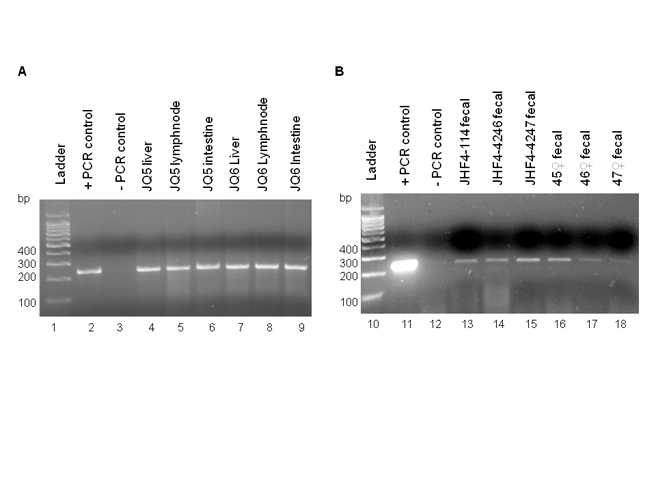

Supplement: Figure S1 — Confirmation of M. ap isolates identity using PCR. Amplification of IS900 fragments from selected clinical samples. PCR reactions show 241 bp band from the infected tissue samples (A) (Lanes: 4–9) and fecal samples (B) (Lanes: 12–18). Origins of the clinical samples along with the example animal identification are listed at the top of the gel. A 100-bp Molecular size marker is included in lanes: 1 and 10. PCR controls are included in lanes: 2, 3, 11 and 12. (TIF) [file pone.0031947.s001.tif]

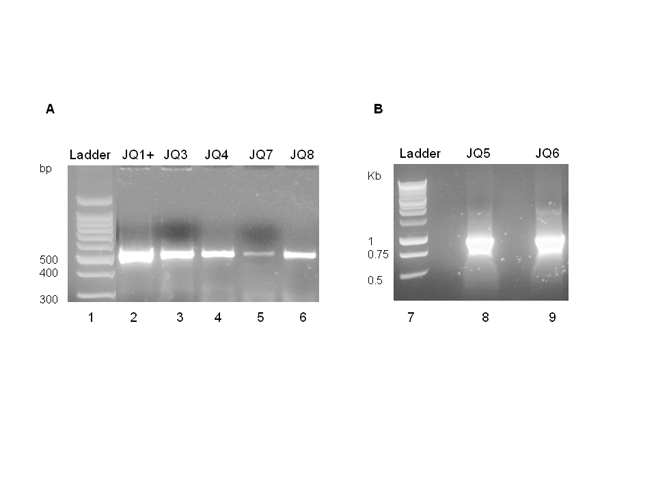

Supplement: Figure S2 — Species-level differentiation of mycobacterial isolates from camels using hsp65 and 16S rRNA gene targets. Ethidium bromide stained agarose gel of PCR reactions show 517 bp band for hsp65 (A) (Lanes: 2–6) and 938 bp band for 16S rRNA (B) (Lane 8 and 9). Top of the gel shows origin of the clinical samples along with the animal identification. (TIF) [file pone.0031947.s002.tif]

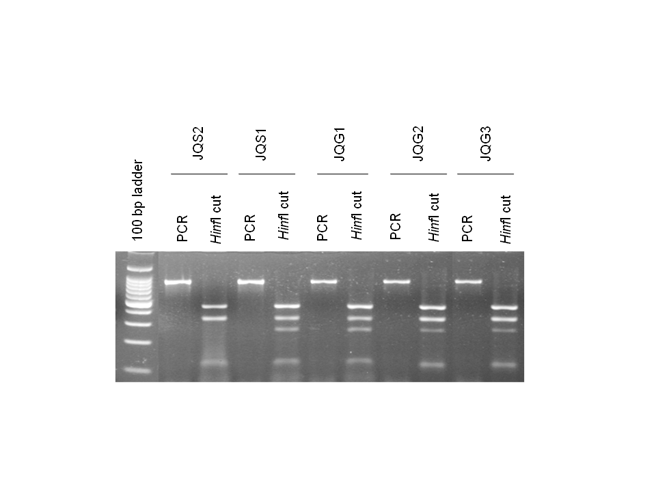

Supplement: Figure S4 — PCR-REA of IS 1311 sequence from sheep and goat infected samples. Ethidium bromide stained 3% agarose gel of PCR amplicons of the IS1311 gene, following REA with Hinf1. For each set, both undigested and digested products (second lane) are shown. The PCR-REA analysis of IS1311 shows presence of mainly M. ap-C strains in the examined goats (JQG1, JQG2, and JQG3) but both M. ap-S and M. ap-C strains are detected in sheep samples (JGS2 and JQS1, respectively). A 100-bp Molecular size marker is shown in the first lane. (TIF) [file pone.0031947.s004.tif]

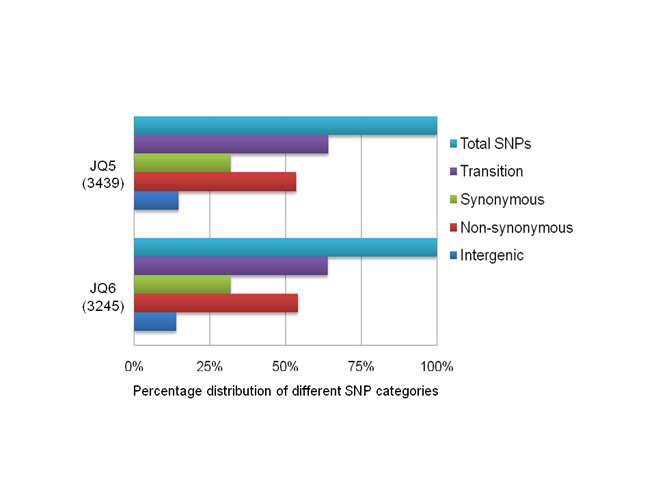

Supplement: Figure S6 — Summary of single nucleotide polymorphisms in M. ap isolates from camel compared to M. ap K-10 strain. The number in parentheses represents the total number of SNPs in each sequenced isolate. The color bars show the percentage distributions of different SNP categories in each isolate. (TIF) [file pone.0031947.s006.tif]

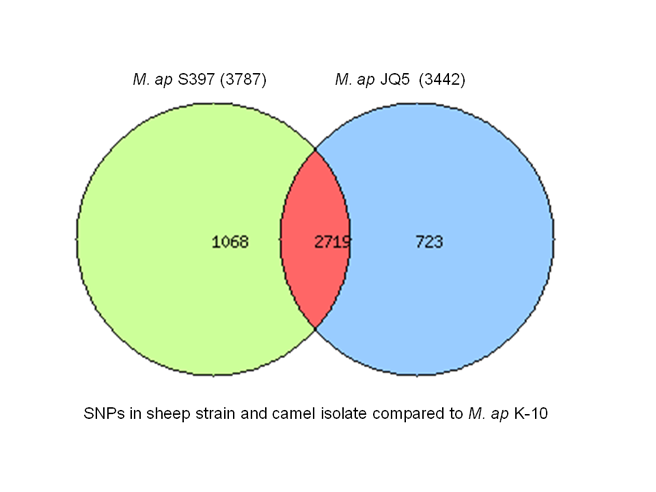

Supplement: Figure S7 — Venn diagram of single nucleotide polymorphisms in sheep strain and camel isolate, JQ5 compared to M. ap K-10. SNP analysis was performed using MAUVE algorithm [52]. (TIF) [file pone.0031947.s007.tif]

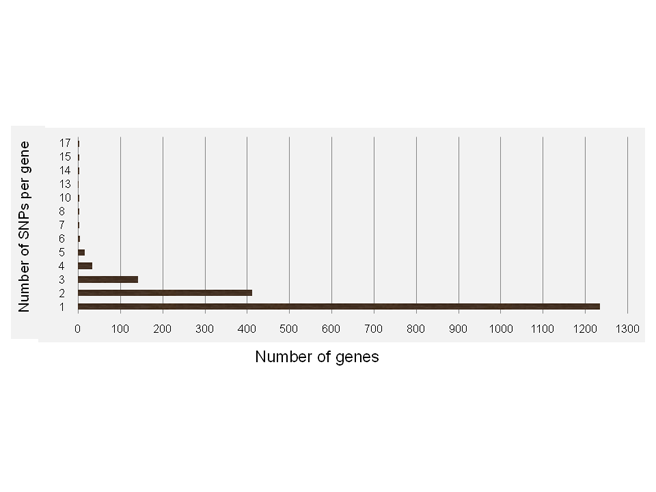

Supplement: Figure S8 — Distribution of number of SNPs per gene. Note the large number of genes with single nucleotide polymorphism. (TIF) [file pone.0031947.s008.tif]

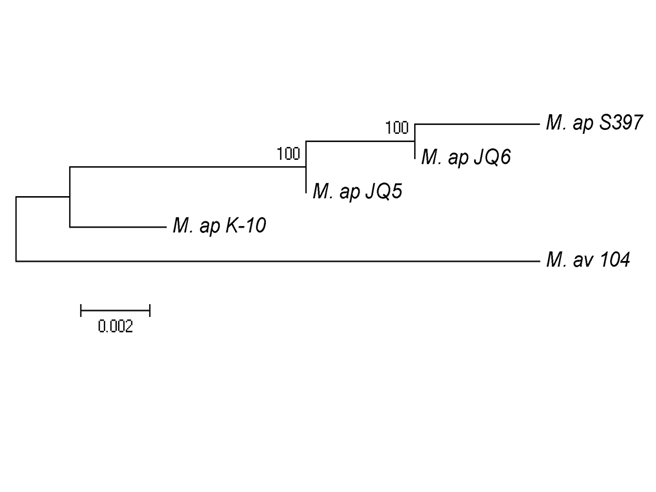

Supplement: Figure S9 — Phylogenetic analysis of mce genes. The phylogenetic tree displaying close relationship between the camel isolates with M. ap-S strain is based on members of mce genes (mceA2, mce1B, mce2, mce3, mce4, and mce1F). The percentage bootstrap values (1000 replicates) are shown next to the branches. The tree is drawn to scale, with branch lengths in the same units as those of the evolutionary distances used to infer the phylogenetic tree. The evolutionary distances were computed using the Maximum Composite Likelihood method using pairwise deletion option. (TIF) [file pone.0031947.s009.tif]
