# Supplementary material for: Genome-Wide Analysis of the Emerging Infection with Mycobacterium avium Subspecies paratuberculosis in the Arabian Camels (Camelus dromedarius)
Source: PLoS One. 2012 Feb 29;7(2):e31947. doi: 10.1371/journal.pone.0031947 (PMC3290536; doi:10.1371/journal.pone.0031947)
Supplement: Table S1 — List of polymorphic sites in 11 house-keeping genes from various M. ap subtypes. (DOC) [file pone.0031947.s010.doc]

**Table S1.** Multi-locus sequence analysis of 11 house-keeping genes in *M. avium* species. All gene positions with variable sites are shown in the concatenated 17,190-bp in-frame semantide.

Gene description and their variable positions are shown:

aspB (aspartate aminotransferase): 1-1167 bp; dnaA: 1168-2697; dnaK: 2698-4569; gnd1 (6-phosphogluconate dehydrogenase): 4570-6054; groEL1 (MAP4265): 6055-7671; GroEL2 : 7672-9297; gyrA: 9298-11817; gyrB: 11818-13851; pepB (leucyl aminopeptidase):13852-15408; recF:15409-16566; sodA: 16567-17190

DataType=Nucleotide; NSites=17190; Identical=.

#M._av_104 GTG ACC GAC CGC GTC TCG CTG CGC GCC GGC ATC CCG CCG TTC TAC [ 45]

#M.ap_K10 ... ... ... ... ... ... ... ... ... ... ... A.A ... ... ... [ 45]

#M._ap_S397 ... ... ... ... ... ... ... ... ... ... ... A.. ... ... ... [ 45]

#M._ap_JQ5 ... ... ... ... ... ... ... ... ... ... ... A.. ... ... ... [ 45]

#M._ap_JQ6 ... ... ... ... ... ... ... ... ... ... ... A.. ... ... ... [ 45]

#M._av_104 GTG ATG GAT GTC TGG CTG GCG GCC GCC GAA AGG CAG CGC AGC CAC [ 90]

#M.ap_K10 ... ... ... ... ... ... ... ..G ... ... ... ... ... ... ... [ 90]

#M._ap_S397 ... ... ... ... ... ... ... ..G ... ... ... ... ... ... ... [ 90]

#M._ap_JQ5 ... ... ... ... ... ... ... ..G ... ... ... ... ... ... ... [ 90]

#M._ap_JQ6 ... ... ... ... ... ... ... ..G ... ... ... ... ... ... ... [ 90]

#M._av_104 GGC GAT TTG GTC AAC CTG TCG GCG GGC CAG CCC AGC GTG GGC GCA [ 135]

#M.ap_K10 ... ... ... ... ... ... ... ... ... ... ... ... ... ... ... [ 135]

#M._ap_S397 ... ... ... ... ... ... ... ... ... ... ... ... ... ... ... [ 135]

#M._ap_JQ5 ... ... ... ... ... ... ... ... ... ... ... ... ... ... ... [ 135]

#M._ap_JQ6 ... ... ... ... ... ... ... ... ... ... ... ... ... ... ... [ 135]

#M._av_104 CCC GAG CCG GTG CGC GCG GCC GCG GCG GCG GCC GTG CAT TCC AAC [ 180]

#M.ap_K10 ... ... ... ... ... ... ... ... ... ... ... ... ... ... ... [ 180]

#M._ap_S397 ... ... ... ... ... ... ... ... ... ... ... ... ... ... ... [ 180]

#M._ap_JQ5 ... ... ... ... ... ... ... ... ... ... ... ... ... ... ... [ 180]

#M._ap_JQ6 ... ... ... ... ... ... ... ... ... ... ... ... ... ... ... [ 180]

#M._av_104 GAG CTG GGC TAC TCG GTG TCG CTG GGC ACG CCG GAG CTG CGG GCC [ 225]

#M.ap_K10 ... ... ... ... ... ... ... ... ... ... ... ... ... ... ... [ 225]

#M._ap_S397 ... ... ... ... ... ... ... ... ... ... ... ... ... ... ... [ 225]

#M._ap_JQ5 ... ... ... ... ... ... ... ... ... ... ... ... ... ... ... [ 225]

#M._ap_JQ6 ... ... ... ... ... ... ... ... ... ... ... ... ... ... ... [ 225]

#M._av_104 GCG ATC GCC GCG GAC TAC CGG CGC CAG CAC GGG CTG GAG GTG GAG [ 270]

#M.ap_K10 ... ... ... ... ... ... ... ... ... ... ... ... ..A ... ... [ 270]

#M._ap_S397 ... ... ... ... ... ... ... ... ... ... ... ... ..A ... ... [ 270]

#M._ap_JQ5 ... ... ... ... ... ... ... ... ... ... ... ... ..A ... ... [ 270]

#M._ap_JQ6 ... ... ... ... ... ... ... ... ... ... ... ... ..A ... ... [ 270]

#M._av_104 CCC GAC GCG GTG GTG ATC ACC ACC GGC TCC TCC GGC GGG TTC CTG [ 315]

#M.ap_K10 ... ... ... ... ... ... ... ... ... ... ... ... ... ... ... [ 315]

#M._ap_S397 ... ... ... ... ... ... ... ... ... ... ... ... ... ... ... [ 315]

#M._ap_JQ5 ... ... ... ... ... ... ... ... ... ... ... ... ... ... ... [ 315]

#M._ap_JQ6 ... ... ... ... ... ... ... ... ... ... ... ... ... ... ... [ 315]

#M._av_104 CTG ACG TTC CTG GCC TGC TTC GAC GTC GGC GAC CGG GTG GCG GTG [ 360]

#M.ap_K10 ... ... ... ... ... ... ... ... ... ... ... ... ... ... C.. [ 360]

#M._ap_S397 ... ... ... ... ... ... ... ... ... ... ... ... ... ... C.. [ 360]

#M._ap_JQ5 ... ... ... ... ... ... ... ... ... ... ... ... ... ... C.. [ 360]

#M._ap_JQ6 ... ... ... ... ... ... ... ... ... ... ... ... ... ... C.. [ 360]

#M._av_104 GCC AGC CCC GGC TAC CCG TGC TAC CGA AAC ATC TTG TCG GCG TTG [ 405]

#M.ap_K10 ... ... ... ... ... ... ... ... ... ... ... ... ... ... ... [ 405]

#M._ap_S397 ... ... ... ... ... ... ... ... ... ... ... ... ... ... ... [ 405]

#M._ap_JQ5 ... ... ... ... ... ... ... ... ... ... ... ... ... ... ... [ 405]

#M._ap_JQ6 ... ... ... ... ... ... ... ... ... ... ... ... ... ... ... [ 405]

#M._av_104 GGA TGT GAG GTG GTG GAG ATC CCC TGC GGG CCG CAG ACC CGG TTC [ 450]

#M.ap_K10 ... ... ... ... ... ... ... ... ... ..A ... ... ... ... ... [ 450]

#M._ap_S397 ... ... ... ... ... ... ... ... ... ..A ... ... ... ... ... [ 450]

#M._ap_JQ5 ... ... ... ... ... ... ... ... ... ..A ... ... ... ... ... [ 450]

#M._ap_JQ6 ... ... ... ... ... ... ... ... ... ..A ... ... ... ... ... [ 450]

#M._av_104 CAG CCC ACC GCG GCG ATG CTC GCC GAA CTC GAC CCG CCG GTG CAG [ 495]

#M.ap_K10 ... ... ... ... ... ... ... ... ... ... ... ... ... ... ... [ 495]

#M._ap_S397 ... ... ... ... ... ... ... ... ... ... ... ... ... ... ... [ 495]

#M._ap_JQ5 ... ... ... ... ... ... ... ... ... ... ... ... ... ... ... [ 495]

#M._ap_JQ6 ... ... ... ... ... ... ... ... ... ... ... ... ... ... ... [ 495]

#M._av_104 GGC GTC ATC GTG GCG AGC CCG GCC AAC CCC ACA GGA ACC GTG ATC [ 540]

#M.ap_K10 ... ... ... ... ... ... ... ... ... ... ..C ... ... ... ... [ 540]

#M._ap_S397 ... ... ... ... ... ... ... ... ... ... ..C ... ... ... ... [ 540]

#M._ap_JQ5 ... ... ... ... ... ... ... ... ... ... ..C ... ... ... ... [ 540]

#M._ap_JQ6 ... ... ... ... ... ... ... ... ... ... ..C ... ... ... ... [ 540]

#M._av_104 GCG CCG GAG GAG TTG GCC GCC ATC GCG TCC TGG TGC GAG GCG TCC [ 585]

#M.ap_K10 ... ... ... ... ... ... ... ... ... ... ... ... ... ... ... [ 585]

#M._ap_S397 ... ... ... ... ... ... ... ... ... ... ... ... ... ... ... [ 585]

#M._ap_JQ5 ... ... ... ... ... ... ... ... ... ... ... ... ... ... ... [ 585]

#M._ap_JQ6 ... ... ... ... ... ... ... ... ... ... ... ... ... ... ... [ 585]

#M._av_104 GGG GCC CGC CTC GTC AGC GAC GAG GTC TAC CAC GGC CTG GTC TAC [ 630]

#M.ap_K10 ... ... ... ... ... ... ... ... ... ... ... ... ... ... ... [ 630]

#M._ap_S397 ... ... ... ... ... ... ... ... ... ... ... ... ... ... ... [ 630]

#M._ap_JQ5 ... ... ... ... ... ... ... ... ... ... ... ... ... ... ... [ 630]

#M._ap_JQ6 ... ... ... ... ... ... ... ... ... ... ... ... ... ... ... [ 630]

#M._av_104 GAG GGG GCG CCA CCC ACC AGC TGC GCG TGG CAG ACC TCG CGG AAT [ 675]

#M.ap_K10 ... ... ... ... ... ... ... ... ... ... ... ... ... ... ... [ 675]

#M._ap_S397 ... ... ... ... ... ... ... ... ... ... ... ... ... ... ... [ 675]

#M._ap_JQ5 ... ... ... ... ... ... ... ... ... ... ... ... ... ... ... [ 675]

#M._ap_JQ6 ... ... ... ... ... ... ... ... ... ... ... ... ... ... ... [ 675]

#M._av_104 GCC GTG GTG GTC AAC AGC TTT TCG AAG TAC TAC GCG ATG ACC GGC [ 720]

#M.ap_K10 ... ... ... ... ... ... ... ... ... ... ... ... ... ... ... [ 720]

#M._ap_S397 ... ... ... ... ... ... ... ... ... ... ... ... ... ... ... [ 720]

#M._ap_JQ5 ... ... ... ... ... ... ... ... ... ... ... ... ... ... ... [ 720]

#M._ap_JQ6 ... ... ... ... ... ... ... ... ... ... ... ... ... ... ... [ 720]

#M._av_104 TGG CGG CTG GGC TGG CTG CTG GTG CCG GTC GAA CTG CGC CGC GCG [ 765]

#M.ap_K10 ... ... ... ... ... ... ... ... ... ... ... ... ... ... ... [ 765]

#M._ap_S397 ... ... ... ... ... ... ... ... ... ... ... ... ... ... ... [ 765]

#M._ap_JQ5 ... ... ... ... ... ... ... ... ... ... ... ... ... ... ... [ 765]

#M._ap_JQ6 ... ... ... ... ... ... ... ... ... ... ... ... ... ... ... [ 765]

#M._av_104 GTG GAA TGC CTG ACC GGC AAC TTC ACC ATC TGC CCG CCC GTG CTG [ 810]

#M.ap_K10 ... ... ... ... ... ... ... ... ... ... ... ... ... ... ... [ 810]

#M._ap_S397 ... ... ... ... ... ... ... ... ... ... ... ... ... ... ... [ 810]

#M._ap_JQ5 ... ... ... ... ... ... ... ... ... ... ... ... ... ... ... [ 810]

#M._ap_JQ6 ... ... ... ... ... ... ... ... ... ... ... ... ... ... ... [ 810]

#M._av_104 TCG CAG CTG GCC GCG GTG GCC GCC TTC ACC CCC GAG GCC ACC GCC [ 855]

#M.ap_K10 ... ... ... ... ... ... ... ... ... ... ... ... ... ... ... [ 855]

#M._ap_S397 ... ... ... ... ... ... ... ... ... ... ... ... ... ... ... [ 855]

#M._ap_JQ5 ... ... ... ... ... ... ... ... ... ... ... ... ... ... ... [ 855]

#M._ap_JQ6 ... ... ... ... ... ... ... ... ... ... ... ... ... ... ... [ 855]

#M._av_104 GAG GCC GAC GGC CAC CTG CAC CAC TAC GCC AAG AAC CGC GCG CTG [ 900]

#M.ap_K10 ... ... ... ..G ... ... ... ... ... ... ... ... ... ... ... [ 900]

#M._ap_S397 ... ... ... ..G ... ... ... ... ... ... ... ... ... ... ... [ 900]

#M._ap_JQ5 ... ... ... ..G ... ... ... ... ... ... ... ... ... ... ... [ 900]

#M._ap_JQ6 ... ... ... ..G ... ... ... ... ... ... ... ... ... ... ... [ 900]

#M._av_104 CTG CTG GAC GGC CTG CGC GGC ATC GGC ATC ACC CGC CTG GCC CCC [ 945]

#M.ap_K10 ... ... ... ... ... ... ... ... ..G ... ... ... ... ... ... [ 945]

#M._ap_S397 ... ... ... ... ... ... ... ... ... ... ... ... ... ... ... [ 945]

#M._ap_JQ5 ... ... ... ... ... ... ... ... ... ... ... ... ... ... ... [ 945]

#M._ap_JQ6 ... ... ... ... ... ... ... ... ... ... ... ... ... ... ... [ 945]

#M._av_104 ACC GAC GGC GCC TTC TAC GTG TAC GCC GAC GTC TCG GAC TTC ACC [ 990]

#M.ap_K10 ... ... ... ... ... ... ... ... ... ... ... ... ... ... ... [ 990]

#M._ap_S397 ... ... ... ... ... ... ... ... ... ... ... ... ... ... ... [ 990]

#M._ap_JQ5 ... ... ... ... ... ... ... ... ... ... ... ... ... ... ... [ 990]

#M._ap_JQ6 ... ... ... ... ... ... ... ... ... ... ... ... ... ... ... [ 990]

#M._av_104 GAC AAC TCC ATG GAG TTC TGC TCG AAG TTG TTG GAG CAG ACC GGC [ 1035]

#M.ap_K10 ... ... ... ... ... ... ... ... ... ... ... ..A ... ... ... [ 1035]

#M._ap_S397 ... ... ... ... ... ... ... ... ... ... ... ..A ... ... ... [ 1035]

#M._ap_JQ5 ... ... ... ... ... ... ... ... ... ... ... ..A ... ... ... [ 1035]

#M._ap_JQ6 ... ... ... ... ... ... ... ... ... ... ... ..A ... ... ... [ 1035]

#M._av_104 GTT GCC ATC GCA CCC GGG ATC GAC TTC GAC ACC ACA CGG GGC AAC [ 1080]

#M.ap_K10 ... ... ... ... ... ... ... ... ... ... ... ... ... ... ... [ 1080]

#M._ap_S397 ... ... ... ... ... ... ... ... ... ... ... ... ... ... ... [ 1080]

#M._ap_JQ5 ... ... ... ... ... ... ... ... ... ... ... ... ... ... ... [ 1080]

#M._ap_JQ6 ... ... ... ... ... ... ... ... ... ... ... ... ... ... ... [ 1080]

#M._av_104 TCG TTC GTC CGG CTG TCC TTC GCC GGC CCC ACC ACC GAC ATC GAG [ 1125]

#M.ap_K10 ... ... ... ... ... ... ... ... ... ... ... ... ... ... ... [ 1125]

#M._ap_S397 ... ... ... ... ... ... ... ... ... ... ... ... ... ... ... [ 1125]

#M._ap_JQ5 ... ... ... ... ... ... ... ... ... ... ... ... ... ... ... [ 1125]

#M._ap_JQ6 ... ... ... ... ... ... ... ... ... ... ... ... ... ... ... [ 1125]

#M._av_104 GAG GCG GTG CGA CGG CTG GGC TCG TGG CTG CGC GCC CGC TAG --- [ 1170]

#M.ap_K10 ... ... ... ... ... ... ... ... ... ... ... ... ... ... TTG [ 1170]

#M._ap_S397 ... ... ... ... ... ... ... ... ... ... ... ... ... ... TTG [ 1170]

#M._ap_JQ5 ... ... ... ... ... ... ... ... ... ... ... ... ... ... TTG [ 1170]

#M._ap_JQ6 ... ... ... ... ... ... ... ... ... ... ... ... ... ... TTG [ 1170]

#M._av_104 --- --- --- --- --- --- --- --- --- --- GTG TGG AAT GCG GTC [ 1215]

#M.ap_K10 GCC GAT GAC CCC GGT TCA AGC TTC ACC ACG ... ... ... ... ... [ 1215]

#M._ap_S397 GCC GAT GAC CCC GGT TCA AGC TTC ACC ACG ... ... ... ... ... [ 1215]

#M._ap_JQ5 GCC GAT GAC CCC GGT TCA AGC TTC ACC ACG ... ... ... ... ... [ 1215]

#M._ap_JQ6 GCC GAT GAC CCC GGT TCA AGC TTC ACC ACG ... ... ... ... ... [ 1215]

#M._av_104 GTT TCG GAG CTC AAC GGC GAG CCC GTC GCC GAC GGC GGA GCC GCC [ 1260]

#M.ap_K10 ... ... ... ... ... ... ... ... ... ... ... ... ... ... ... [ 1260]

#M._ap_S397 ... ... ... ... ... ... ... ... ... ... ... ... ... ... ... [ 1260]

#M._ap_JQ5 ... ... ... ... ... ... ... ... ... ... ... ... ... ... ... [ 1260]

#M._ap_JQ6 ... ... ... ... ... ... ... ... ... ... ... ... ... ... ... [ 1260]

#M._av_104 AAC CGC ACG ACT CTG GTC ACT CCC CTC ACC CCT CAG CAA AGA GCG [ 1305]

#M.ap_K10 ... ... ... ... ... ... ... ... ... ... ... ... ... ... ... [ 1305]

#M._ap_S397 ... ... ... ... ... ... ... ... ... ... ... ... ... ... ... [ 1305]

#M._ap_JQ5 ... ... ... ... ... ... ... ... ... ... ... ... ... ... ... [ 1305]

#M._ap_JQ6 ... ... ... ... ... ... ... ... ... ... ... ... ... ... ... [ 1305]

#M._av_104 TGG CTC AAT CTG GTC CGC CCG CTG ACC ATC GTC GAG GGG TTT GCT [ 1350]

#M.ap_K10 ... ... ... ... ... ... ... ... ... ... ... ... ... ... ... [ 1350]

#M._ap_S397 ... ... ... ... ... ... ... ... ... ... ... ... ... ... ... [ 1350]

#M._ap_JQ5 ... ... ... ... ... ... ... ... ... ... ... ... ... ... ... [ 1350]

#M._ap_JQ6 ... ... ... ... ... ... ... ... ... ... ... ... ... ... ... [ 1350]

#M._av_104 CTG CTG TCA GTG CCG AGC AGT TTC GTG CAG AAC GAG ATC GAA CGG [ 1395]

#M.ap_K10 ... ... ..G ... ... ... ... ... ... ... ... ... ... ... ... [ 1395]

#M._ap_S397 ... ... ..G ... ... ... ... ... ... ... ... ... ... ... ... [ 1395]

#M._ap_JQ5 ... ... ..G ... ... ... ... ... ... ... ... ... ... ... ... [ 1395]

#M._ap_JQ6 ... ... ..G ... ... ... ... ... ... ... ... ... ... ... ... [ 1395]

#M._av_104 CAC CTG CGC GCC CCG ATC ACC GAC GCG CTC AGC CGC CGC CTG GGT [ 1440]

#M.ap_K10 ... ... ... ... ... ... ... ... ... ... ... ... ... ... ... [ 1440]

#M._ap_S397 ... ... ... ... ... ... ... ... ... ... ... ... ... ... ... [ 1440]

#M._ap_JQ5 ... ... ... ... ... ... ... ... ... ... ... ... ... ... ... [ 1440]

#M._ap_JQ6 ... ... ... ... ... ... ... ... ... ... ... ... ... ... ... [ 1440]

#M._av_104 CAG CAG ATC CAG CTG GGG GTC CGC ATC GCT CCC CCA CCC GAC GAC [ 1485]

#M.ap_K10 ... ... ... ... ... ..A ... ... ... ... ... ... ... ... ... [ 1485]

#M._ap_S397 ... ... ... ... ... ..A ... ... ... ... ... ... ... ... ... [ 1485]

#M._ap_JQ5 ... ... ... ... ... ..A ... ... ... ... ... ... ... ... ... [ 1485]

#M._ap_JQ6 ... ... ... ... ... ..A ... ... ... ... ... ... ... ... ... [ 1485]

#M._av_104 GTC GAG GAC GCG CTC ATC CCG CCG GCC GAG CCG TTC CCC GAC ACC [ 1530]

#M.ap_K10 ... ... ... ... .C. ... ... ... ... ... ... ... ... ... ... [ 1530]

#M._ap_S397 ... ... ... ... ... ... ... ... ... ... ... ... ... ... ... [ 1530]

#M._ap_JQ5 ... ... ... ... ... ... ... ... ... ... ... ... ... ... ... [ 1530]

#M._ap_JQ6 ... ... ... ... ... ... ... ... ... ... ... ... ... ... ... [ 1530]

#M._av_104 GAC GCC GCC CTG TCC GCC GAC GAC GGC GCC GAC GGC GAG CCG GTG [ 1575]

#M.ap_K10 ... ... ... ... ... ... ... ... ... ... ... ... ... ... ... [ 1575]

#M._ap_S397 ... ... ... ... ... ... ... ... ... ... ... ... ... ... ... [ 1575]

#M._ap_JQ5 ... ... ... ... ... ... ... ... ... ... ... ... ... ... ... [ 1575]

#M._ap_JQ6 ... ... ... ... ... ... ... ... ... ... ... ... ... ... ... [ 1575]

#M._av_104 GAG AAC GGG GAG CCG GTC ACC GAC ACC CAG CCC GGC TGG CCC AAC [ 1620]

#M.ap_K10 ... ... ... ... ... ... ... ... ... ... ... ... ... ... ... [ 1620]

#M._ap_S397 ... ... ... ... ... ... ... ... ... ... ... ... ... ... ... [ 1620]

#M._ap_JQ5 ... ... ... ... ... ... ... ... ... ... ... ... ... ... ... [ 1620]

#M._ap_JQ6 ... ... ... ... ... ... ... ... ... ... ... ... ... ... ... [ 1620]

#M._av_104 TAC TTC ACC GAG CGG CCG CAC GCC ATC GAT CCC GCC GTC GCC GCC [ 1665]

#M.ap_K10 ... ... ... ... ... ... ... ... ... ... ... ... ... ... ... [ 1665]

#M._ap_S397 ... ... ... ... ... ... ... ... ... ... ... ... ... ... ... [ 1665]

#M._ap_JQ5 ... ... ... ... ... ... ... ... ... ... ... ... ... ... ... [ 1665]

#M._ap_JQ6 ... ... ... ... ... ... ... ... ... ... ... ... ... ... ... [ 1665]

#M._av_104 GGA ACG AGC CTC AAC CGC CGC TAC ACC TTC GAC ACG TTC GTG ATC [ 1710]

#M.ap_K10 ... ... ... ... ... ... ... ... ... ... ... ... ... ... ... [ 1710]

#M._ap_S397 ... ... ... ... ... ... ... ... ... ... ... ... ... ... ... [ 1710]

#M._ap_JQ5 ... ... ... ... ... ... ... ... ... ... ... ... ... ... ... [ 1710]

#M._ap_JQ6 ... ... ... ... ... ... ... ... ... ... ... ... ... ... ... [ 1710]

#M._av_104 GGG GCG TCC AAC CGG TTC GCG CAC GCC GCC GCC CTG GCC ATC GCC [ 1755]

#M.ap_K10 ... ... ... ... ... ... ... ... ... ... ... ... ... ... ... [ 1755]

#M._ap_S397 ... ... ... ... ... ... ... ... ... ... ... ... ... ... ... [ 1755]

#M._ap_JQ5 ... ... ... ... ... ... ... ... ... ... ... ... ... ... ... [ 1755]

#M._ap_JQ6 ... ... ... ... ... ... ... ... ... ... ... ... ... ... ... [ 1755]

#M._av_104 GAA GCA CCG GCC CGC GCC TAC AAC CCG TTG TTC ATC TGG GGC GAG [ 1800]

#M.ap_K10 ... ... ... ... ... ... ... ... ... ... ... ... ... ... ... [ 1800]

#M._ap_S397 ... ... ... ... ... ... ... ... ... ... ... ... ... ... ... [ 1800]

#M._ap_JQ5 ... ... ... ... ... ... ... ... ... ... ... ... ... ... ... [ 1800]

#M._ap_JQ6 ... ... ... ... ... ... ... ... ... ... ... ... ... ... ... [ 1800]

#M._av_104 TCC GGT CTG GGC AAG ACG CAC CTG CTG CAC GCC GCC GGC AAT TAC [ 1845]

#M.ap_K10 ... ... ... ... ... ... ... ... ... ... ... ... ... ... ... [ 1845]

#M._ap_S397 ... ... ... ... ... ... ... ... ... ... ... ... ... ... ... [ 1845]

#M._ap_JQ5 ... ... ... ... ... ... ... ... ... ... ... ... ... ... ... [ 1845]

#M._ap_JQ6 ... ... ... ... ... ... ... ... ... ... ... ... ... ... ... [ 1845]

#M._av_104 GCG CAG CGG CTC TTC CCC GGC ATG CGG GTC AAG TAC GTC TCC ACC [ 1890]

#M.ap_K10 ... ..A ... ... ... ... ... ... ... ... ... ... ... ... ... [ 1890]

#M._ap_S397 ... ... ... ... ... ... ... ... ... ... ... ... ... ... ... [ 1890]

#M._ap_JQ5 ... ... ... ... ... ... ... ... ... ... ... ... ... ... ... [ 1890]

#M._ap_JQ6 ... ... ... ... ... ... ... ... ... ... ... ... ... ... ... [ 1890]

#M._av_104 GAG GAA TTC ACC AAC GAC TTC ATC AAC TCG CTG CGC GAC GAC CGC [ 1935]

#M.ap_K10 ... ... ... ..G ... ... ... ... ... .T. ... ... ... ... ... [ 1935]

#M._ap_S397 ... ... ... ..G ... ... ... ... ... ... ... ... ... ... ... [ 1935]

#M._ap_JQ5 ... ... ... ..G ... ... ... ... ... ... ... ... ... ... ... [ 1935]

#M._ap_JQ6 ... ... ... ..G ... ... ... ... ... ... ... ... ... ... ... [ 1935]

#M._av_104 AAG GTC GCC TTC AAG CGC AGC TAT CGC GAC GTC GAC GTG CTG CTG [ 1980]

#M.ap_K10 ... ... ... ... ... ... ... ... ... ... ... ... ... ..C ... [ 1980]

#M._ap_S397 ... ... ... ... ... ... ... ... ... ... ... ... ... ..C ... [ 1980]

#M._ap_JQ5 ... ... ... ... ... ... ... ... ... ... ... ... ... ..C ... [ 1980]

#M._ap_JQ6 ... ... ... ... ... ... ... ... ... ... ... ... ... ..C ... [ 1980]

#M._av_104 GTC GAT GAC ATC CAG TTC ATC GAG GGC AAG GAA GGC ATC CAG GAG [ 2025]

#M.ap_K10 ... ... ... ... ..A ... ... ... ... ... ... ... ... ... ... [ 2025]

#M._ap_S397 ... ... ... ... ..A ... ... ... ... ... ... ... ... ... ... [ 2025]

#M._ap_JQ5 ... ... ... ... ..A ... ... ... ... ... ... ... ... ... ... [ 2025]

#M._ap_JQ6 ... ... ... ... ..A ... ... ... ... ... ... ... ... ... ... [ 2025]

#M._av_104 GAG TTC TTC CAC ACC TTC AAC ACG CTG CAC AAC GCC AAC AAG CAG [ 2070]

#M.ap_K10 ... ... ... ... ... ... ... ... ... ... ... ... ... ... ... [ 2070]

#M._ap_S397 ... ... ... ... ... ... ... ... ... ... ... ... ... ... ... [ 2070]

#M._ap_JQ5 ... ... ... ... ... ... ... ... ... ... ... ... ... ... ... [ 2070]

#M._ap_JQ6 ... ... ... ... ... ... ... ... ... ... ... ... ... ... ... [ 2070]

#M._av_104 ATC GTC ATC TCC TCC GAC CGG CCG CCC AAA CAG CTG GCC ACC CTG [ 2115]

#M.ap_K10 ... ... ... ... ... ... ... ... ... ... ... ... ... ... ... [ 2115]

#M._ap_S397 ... ... ... ... ... ... ... ... ... ... ... ... ... ... ... [ 2115]

#M._ap_JQ5 ... ... ... ... ... ... ... ... ... ... ... ... ... ... ... [ 2115]

#M._ap_JQ6 ... ... ... ... ... ... ... ... ... ... ... ... ... ... ... [ 2115]

#M._av_104 GAA GAC CGG CTG CGA ACC CGG TTC GAG TGG GGC CTG ATC ACC GAC [ 2160]

#M.ap_K10 ... ... ... ... ... ... ... ... ... ... ... ... ... ... ... [ 2160]

#M._ap_S397 ... ... ... ... ... ... ... ... ... ... ... ... ... ... ... [ 2160]

#M._ap_JQ5 ... ... ... ... ... ... ... ... ... ... ... ... ... ... ... [ 2160]

#M._ap_JQ6 ... ... ... ... ... ... ... ... ... ... ... ... ... ... ... [ 2160]

#M._av_104 GTG CAG CCC CCC GAA CTC GAA ACC CGC ATC GCG ATC CTG CGC AAG [ 2205]

#M.ap_K10 ... ... ... ... ... ... ... ... ... ... ... ... ... ... ... [ 2205]

#M._ap_S397 ... ... ... ... ... ... ... ... ... ... ... ... ... ... ... [ 2205]

#M._ap_JQ5 ... ... ... ... ... ... ... ... ... ... ... ... ... ... ... [ 2205]

#M._ap_JQ6 ... ... ... ... ... ... ... ... ... ... ... ... ... ... ... [ 2205]

#M._av_104 AAG GCA CAG ATG GAG CGC CTG GCG GTG CCC GAC GAC GTG CTG GAA [ 2250]

#M.ap_K10 ... ..G ... ... ... ... ... ... ... ... ... ... ... ... ... [ 2250]

#M._ap_S397 ... ..G ... ... ... ... ... ... ... ... ... ... ... ... ... [ 2250]

#M._ap_JQ5 ... ..G ... ... ... ... ... ... ... ... ... ... ... ... ... [ 2250]

#M._ap_JQ6 ... ..G ... ... ... ... ... ... ... ... ... ... ... ... ... [ 2250]

#M._av_104 CTC ATC GCC AGC AGC ATC GAG CGC AAC ATC CGC GAA CTC GAG GGC [ 2295]

#M.ap_K10 ... ... ... ... ... ... ... ... ... ... ... ... ... ... ... [ 2295]

#M._ap_S397 ... ... ... ... ... ... ... ... ... ... ... ... ... ... ... [ 2295]

#M._ap_JQ5 ... ... ... ... ... ... ... ... ... ... ... ... ... ... ... [ 2295]

#M._ap_JQ6 ... ... ... ... ... ... ... ... ... ... ... ... ... ... ... [ 2295]

#M._av_104 GCC CTG ATC CGG GTC ACC GCG TTC GCC TCG CTG AAC AAG ACT CCG [ 2340]

#M.ap_K10 ... ... ... ... ... ... ... ... ... ... ... ... ... ..C ... [ 2340]

#M._ap_S397 ... ... ... ... ... ... ... ... ... ... ... ... ... ..C ... [ 2340]

#M._ap_JQ5 ... ... ... ... ... ... ... ... ... ... ... ... ... ..C ... [ 2340]

#M._ap_JQ6 ... ... ... ... ... ... ... ... ... ... ... ... ... ..C ... [ 2340]

#M._av_104 ATC GAC AAG TCG CTG GCC GAG ATC GTG CTG CGC GAT TTG ATC GCC [ 2385]

#M.ap_K10 ... ... ... ... ... ... ... ... ... ... ... ... ... ..T ... [ 2385]

#M._ap_S397 ... ... ... ... ... ... ... ... ... ... ... ... ... ..T ... [ 2385]

#M._ap_JQ5 ... ... ... ... ... ... ... ... ... ... ... ... ... ..T ... [ 2385]

#M._ap_JQ6 ... ... ... ... ... ... ... ... ... ... ... ... ... ..T ... [ 2385]

#M._av_104 GAC GCC AGC ACC ATG CAG ATC AGC GCG GCC ACC ATC ATG GCC GCC [ 2430]

#M.ap_K10 ... ... ... ... ... ... ... ... ... ... ... ... ... ... ... [ 2430]

#M._ap_S397 ... ... ... ... ... ... ... ... ... ... ... ... ... ... ... [ 2430]

#M._ap_JQ5 ... ... ... ... ... ... ... ... ... ... ... ... ... ... ... [ 2430]

#M._ap_JQ6 ... ... ... ... ... ... ... ... ... ... ... ... ... ... ... [ 2430]

#M._av_104 ACC GCC GAA TAC TTC GAC ACC ACC GTC GAG GAA CTG CGC GGG CCG [ 2475]

#M.ap_K10 ... ... ... ... ... ... ... ... ... ... ... ... ... ... ... [ 2475]

#M._ap_S397 ... ... ... ... ... ... ... ... ... ... ... ... ... ... ... [ 2475]

#M._ap_JQ5 ... ... ... ... ... ... ... ... ... ... ... ... ... ... ... [ 2475]

#M._ap_JQ6 ... ... ... ... ... ... ... ... ... ... ... ... ... ... ... [ 2475]

#M._av_104 GGC AAG ACC CGG GCG CTG GCC CAG TCC CGT CAA ATC GCG ATG TAC [ 2520]

#M.ap_K10 ... ... ... ... ... ... ... ... ... ..C ... ... ... ... ... [ 2520]

#M._ap_S397 ... ... ... ... ... ... ... ... ... ..C ... ... ... ... ... [ 2520]

#M._ap_JQ5 ... ... ... ... ... ... ... ... ... ..C ... ... ... ... ... [ 2520]

#M._ap_JQ6 ... ... ... ... ... ... ... ... ... ..C ... ... ... ... ... [ 2520]

#M._av_104 CTG TGC CGC GAG CTC ACG GAT CTG TCG CTG CCC AAG ATC GGG CAG [ 2565]

#M.ap_K10 ... ... ... ... ... ... ... ... ... ... ... ... ... ... ... [ 2565]

#M._ap_S397 ... ... ... ... ... ... ... ... ... ... ... ... ... ... ... [ 2565]

#M._ap_JQ5 ... ... ... ... ... ... ... ... ... ... ... ... ... ... ... [ 2565]

#M._ap_JQ6 ... ... ... ... ... ... ... ... ... ... ... ... ... ... ... [ 2565]

#M._av_104 GCC TTC GGC CGC GAC CAC ACC ACG GTG ATG TAC GCC CAG CGC AAG [ 2610]

#M.ap_K10 ... ... ... ... ... ... ... ... ... ... ... ... ... ... ... [ 2610]

#M._ap_S397 ... ... ... ... ... ... ... ... ... ... ... ... ... ... ... [ 2610]

#M._ap_JQ5 ... ... ... ... ... ... ... ... ... ... ... ... ... ... ... [ 2610]

#M._ap_JQ6 ... ... ... ... ... ... ... ... ... ... ... ... ... ... ... [ 2610]

#M._av_104 ATC CTG TCC GAG ATG GCC GAG CGA CGC GAG GTG TTC GAT CAC GTC [ 2655]

#M.ap_K10 ... ... ... ... ... ... ... ... ... ... ... ... ... ... ... [ 2655]

#M._ap_S397 ... ... ... ... ... ... ... ... ... ... ... ... ... ... ... [ 2655]

#M._ap_JQ5 ... ... ... ... ... ... ... ... ... ... ... ... ... ... ... [ 2655]

#M._ap_JQ6 ... ... ... ... ... ... ... ... ... ... ... ... ... ... ... [ 2655]

#M._av_104 AAG GAG CTC ACC ACT CGC ATT CGT CAG CGC TCC AAG CGC TGA ATG [ 2700]

#M.ap_K10 ... ... ... ... ... ... ... ... ... ... ... ... ... ... ... [ 2700]

#M._ap_S397 ... ... ... ... ... ... ... ... ... ... ... ... ... ... ... [ 2700]

#M._ap_JQ5 ... ... ... ... ... ... ... ... ... ... ... ... ... ... ... [ 2700]

#M._ap_JQ6 ... ... ... ... ... ... ... ... ... ... ... ... ... ... ... [ 2700]

#M._av_104 GCT CGT GCG GTC GGT ATC GAC CTC GGG ACC ACC AAC TCC GTC GTC [ 2745]

#M.ap_K10 ... ... ... ... ... ... ... ... ... ... ... ... ... ... ... [ 2745]

#M._ap_S397 ... ... ... ... ... ... ... ... ... ... ... ... ... ... ... [ 2745]

#M._ap_JQ5 ... ... ... ... ... ... ... ... ... ... ... ... ... ... ... [ 2745]

#M._ap_JQ6 ... ... ... ... ... ... ... ... ... ... ... ... ... ... ... [ 2745]

#M._av_104 GCA GTC CTC GAG GGC GGT GAC CCC GTC GTC GTC GCC AAC TCC GAA [ 2790]

#M.ap_K10 ... ... ... ... ... ... ... ... ... ... ... ... ... ... ..G [ 2790]

#M._ap_S397 ... ... ... ... ... ... ... ... ... ... ... ... ... ... ..G [ 2790]

#M._ap_JQ5 ... ... ... ... ... ... ... ... ... ... ... ... ... ... ..G [ 2790]

#M._ap_JQ6 ... ... ... ... ... ... ... ... ... ... ... ... ... ... ..G [ 2790]

#M._av_104 GGC TCG CGG ACC ACC CCG TCC ATC GTC GCG TTC GCC CGC AAC GGC [ 2835]

#M.ap_K10 ... ... ... ... ... ... ... ... ... ... ... ... ... ... ... [ 2835]

#M._ap_S397 ... ... ... ... ... ... ... ... ... ... ... ... ... ... ... [ 2835]

#M._ap_JQ5 ... ... ... ... ... ... ... ... ... ... ... ... ... ... ... [ 2835]

#M._ap_JQ6 ... ... ... ... ... ... ... ... ... ... ... ... ... ... ... [ 2835]

#M._av_104 GAG GTG CTC GTC GGC CAG CCC GCC AAG AAC CAG GCG GTG ACC AAC [ 2880]

#M.ap_K10 ... ... ... ... ... ... ... ... ... ... ... ... ... ... ... [ 2880]

#M._ap_S397 ... ... ... ... ... ... ... ... ... ... ... ... ... ... ... [ 2880]

#M._ap_JQ5 ... ... ... ... ... ... ... ... ... ... ... ... ... ... ... [ 2880]

#M._ap_JQ6 ... ... ... ... ... ... ... ... ... ... ... ... ... ... ... [ 2880]

#M._av_104 GTC GAC CGC ACC ATC CGT TCG GTC AAG CGG CAC ATG GGC ACC GAC [ 2925]

#M.ap_K10 ... ... ... ... ... ... ... ... ... ... ... ... ... ... ... [ 2925]

#M._ap_S397 ... ... ... ... ... ... ... ... ... ... ... ... ... ... ... [ 2925]

#M._ap_JQ5 ... ... ... ... ... ... ... ... ... ... ... ... ... ... ... [ 2925]

#M._ap_JQ6 ... ... ... ... ... ... ... ... ... ... ... ... ... ... ... [ 2925]

#M._av_104 TGG TCC ATC GAG ATC GAC GGC AAG AAA TAC ACC GCT CAG GAG ATC [ 2970]

#M.ap_K10 ... ... ... ... ... ... ... ... ... ... ... ... ... ... ... [ 2970]

#M._ap_S397 ... ... ... ... ... ... ... ... ... ... ... ... ... ... ... [ 2970]

#M._ap_JQ5 ... ... ... ... ... ... ... ... ... ... ... ... ... ... ... [ 2970]

#M._ap_JQ6 ... ... ... ... ... ... ... ... ... ... ... ... ... ... ... [ 2970]

#M._av_104 AGC GCC CGC GTG CTG ATG AAG CTC AAG CGC GAC GCC GAG GCC TAT [ 3015]

#M.ap_K10 ... ... ... ... ... ... ... ... ... ... ... ... ... ... ... [ 3015]

#M._ap_S397 ... ... ... ... ... ... ... ... ... ... ... ... ... ... ... [ 3015]

#M._ap_JQ5 ... ... ... ... ... ... ... ... ... ... ... ... ... ... ... [ 3015]

#M._ap_JQ6 ... ... ... ... ... ... ... ... ... ... ... ... ... ... ... [ 3015]

#M._av_104 CTG GGT GAG GAC ATC ACC GAC GCG GTC ATC ACC GTA CCG GCG TAC [ 3060]

#M.ap_K10 ... ... ... ... ... ... ... ... ... ... ... ... ... ... ... [ 3060]

#M._ap_S397 ... ... ... ... ... ... ... A.. ... ... ... ... ... ... ... [ 3060]

#M._ap_JQ5 ... ... ... ... ... ... ... A.. ... ... ... ... ... ... ... [ 3060]

#M._ap_JQ6 ... ... ... ... ... ... ... A.. ... ... ... ... ... ... ... [ 3060]

#M._av_104 TTC AAC GAC GCC CAG CGT CAG GCG ACC AAG GAA GCC GGC CAG ATC [ 3105]

#M.ap_K10 ... ... ... ... ... ... ... ... ... ... ... ... ... ... ... [ 3105]

#M._ap_S397 ... ... ... ... ... ... ... ... ... ... ... ... ... ... ... [ 3105]

#M._ap_JQ5 ... ... ... ... ... ... ... ... ... ... ... ... ... ... ... [ 3105]

#M._ap_JQ6 ... ... ... ... ... ... ... ... ... ... ... ... ... ... ... [ 3105]

#M._av_104 GCC GGC CTC AAC GTG CTG CGC ATC GTC AAC GAG CCG ACC GCG GCC [ 3150]

#M.ap_K10 ... ... ... ... ... ... ... ... ... ... ... ... ... ... ... [ 3150]

#M._ap_S397 ... ... ... ... ... ... ... ... ... ... ... ... ... ... ... [ 3150]

#M._ap_JQ5 ... ... ... ... ... ... ... ... ... ... ... ... ... ... ... [ 3150]

#M._ap_JQ6 ... ... ... ... ... ... ... ... ... ... ... ... ... ... ... [ 3150]

#M._av_104 GCG CTG GCC TAC GGC CTG GAC AAG GGC GAG AAG GAG CAG ACC ATC [ 3195]

#M.ap_K10 ... ... ... ... ... ... ... ... ... ... ... ... ... ... ... [ 3195]

#M._ap_S397 ... ... ... ... ... ... ... ... ... ... ... ... ... ... ... [ 3195]

#M._ap_JQ5 ... ... ... ... ... ... ... ... ... ... ... ... ... ... ... [ 3195]

#M._ap_JQ6 ... ... ... ... ... ... ... ... ... ... ... ... ... ... ... [ 3195]

#M._av_104 CTG GTC TTC GAC CTC GGC GGC GGC ACG TTC GAC GTC TCG CTG CTC [ 3240]

#M.ap_K10 ... ... ... ... ... ... ... ... ... ... ... ..T ... ... ... [ 3240]

#M._ap_S397 ... ... ... ... ... ... ... ... ... ... ... ..T ... ... ... [ 3240]

#M._ap_JQ5 ... ... ... ... ... ... ... ... ... ... ... ..T ... ... ... [ 3240]

#M._ap_JQ6 ... ... ... ... ... ... ... ... ... ... ... ..T ... ... ... [ 3240]

#M._av_104 GAG ATC GGC GAG GGT GTG GTC GAG GTC CGC GCC ACC AGC GGT GAC [ 3285]

#M.ap_K10 ... ... ... ... ... ... ... ... ... ... ... ... ... ... ... [ 3285]

#M._ap_S397 ... ... ... ... ... ... ... ... ... ... ... ... ... ... ... [ 3285]

#M._ap_JQ5 ... ... ... ... ... ... ... ... ... ... ... ... ... ... ... [ 3285]

#M._ap_JQ6 ... ... ... ... ... ... ... ... ... ... ... ... ... ... ... [ 3285]

#M._av_104 AAC CAC CTC GGT GGC GAC GAC TGG GAC GAC CGG ATC GTC AAC TGG [ 3330]

#M.ap_K10 ... ..A ... ... ... ... ... ... ... ... ... ... ... ... ... [ 3330]

#M._ap_S397 ... ... ... ... ... ... ... ... ... ... ... ... ... ... ... [ 3330]

#M._ap_JQ5 ... ... ... ... ... ... ... ... ... ... ... ... ... ... ... [ 3330]

#M._ap_JQ6 ... ... ... ... ... ... ... ... ... ... ... ... ... ... ... [ 3330]

#M._av_104 CTG GTC GAC AAG TTC AAG GGC ACC AGC GGC ATC GAC CTG ACC AAG [ 3375]

#M.ap_K10 ... ... ... ... ... ... ... ... ... ... ... ... ... ... ... [ 3375]

#M._ap_S397 ... ... ... ... ... ... ... ... ... ... ... ... ... ... ... [ 3375]

#M._ap_JQ5 ... ... ... ... ... ... ... ... ... ... ... ... ... ... ... [ 3375]

#M._ap_JQ6 ... ... ... ... ... ... ... ... ... ... ... ... ... ... ... [ 3375]

#M._av_104 GAC AAG ATG GCC ATG CAG CGG CTA CGT GAG GCC GCC GAG AAG GCC [ 3420]

#M.ap_K10 ... ... ... ... ... ... ... ..G ... ... ... ... ... ... ... [ 3420]

#M._ap_S397 ... ... ... ... ... ... ... ..G ... ... ... ... ... ... ... [ 3420]

#M._ap_JQ5 ... ... ... ... ... ... ... ..G ... ... ... ... ... ... ... [ 3420]

#M._ap_JQ6 ... ... ... ... ... ... ... ..G ... ... ... ... ... ... ... [ 3420]

#M._av_104 AAG ATC GAG CTG TCC AGC TCG CAG AGC ACC TCG ATC AAC CTG CCC [ 3465]

#M.ap_K10 ... ... ... ... ... ... ... ... ... ... ... ... ... ... ... [ 3465]

#M._ap_S397 ... ... ... ... ... ... ... ... ... ... ... ... ... ... ... [ 3465]

#M._ap_JQ5 ... ... ... ... ... ... ... ... ... ... ... ... ... ... ... [ 3465]

#M._ap_JQ6 ... ... ... ... ... ... ... ... ... ... ... ... ... ... ... [ 3465]

#M._av_104 TAC ATC ACC GTC GAC GCG GAC AAG AAC CCG CTG TTC CTC GAC GAG [ 3510]

#M.ap_K10 ... ... ... ... ... ... ... ... ... ... ... ... ... ... ... [ 3510]

#M._ap_S397 ... ... ... ... ... ... ... ... ... ... ... ... ... ... ... [ 3510]

#M._ap_JQ5 ... ... ... ... ... ... ... ... ... ... ... ... ... ... ... [ 3510]

#M._ap_JQ6 ... ... ... ... ... ... ... ... ... ... ... ... ... ... ... [ 3510]

#M._av_104 CAG CTG ACC CGC GCC GAA TTC CAG CGC ATC ACC CAG GAT CTG CTG [ 3555]

#M.ap_K10 ... ... ... ... ... ... ... ... ... ... ... ... ... ... ... [ 3555]

#M._ap_S397 ... ... ... ... ... ... ... ... ... ... ... ... ... ... ... [ 3555]

#M._ap_JQ5 ... ... ... ... ... ... ... ... ... ... ... ... ... ... ... [ 3555]

#M._ap_JQ6 ... ... ... ... ... ... ... ... ... ... ... ... ... ... ... [ 3555]

#M._av_104 GAC CGC ACC CGT CAG CCG TTC AAG TCG GTG ATC GCC GAC GCC GGC [ 3600]

#M.ap_K10 ... ... ... ... ... ... ... ... ... ... ... ... ... ... ... [ 3600]

#M._ap_S397 ... ... ... ... ... ... ... ... ... ... ... ... ... ... ... [ 3600]

#M._ap_JQ5 ... ... ... ... ... ... ... ... ... ... ... ... ... ... ... [ 3600]

#M._ap_JQ6 ... ... ... ... ... ... ... ... ... ... ... ... ... ... ... [ 3600]

#M._av_104 ATC TCG GTG TCC GAC ATC GAC CAC GTG GTG CTG GTG GGT GGT TCC [ 3645]

#M.ap_K10 ... ... ... ... ... ... ... ... ... ... ... ... ... ... ... [ 3645]

#M._ap_S397 ... ... ... ... ... ... ... ... ... ... ... ... ... ... ... [ 3645]

#M._ap_JQ5 ... ... ... ... ... ... ... ... ... ... ... ... ... ... ... [ 3645]

#M._ap_JQ6 ... ... ... ... ... ... ... ... ... ... ... ... ... ... ... [ 3645]

#M._av_104 ACC CGG ATG CCC GCG GTG ACC GAC CTG GTC AAG GAA CTC ACC GGC [ 3690]

#M.ap_K10 ... ... ... ... ... ... ... ... ... ... ... ... ... ... ... [ 3690]

#M._ap_S397 ... ... ... ... ... ... ... ... ... ... ... ... ... ... ... [ 3690]

#M._ap_JQ5 ... ... ... ... ... ... ... ... ... ... ... ... ... ... ... [ 3690]

#M._ap_JQ6 ... ... ... ... ... ... ... ... ... ... ... ... ... ... ... [ 3690]

#M._av_104 GGC AAG GAG CCC AAC AAG GGC GTC AAC CCC GAC GAG GTT GTC GCG [ 3735]

#M.ap_K10 ... ... ... ... ... ... ... ... ... ... ... ... ... ... ... [ 3735]

#M._ap_S397 ... ... ... ... ... ... ... ... ... ... ... ... ... ... ... [ 3735]

#M._ap_JQ5 ... ... ... ... ... ... ... ... ... ... ... ... ... ... ... [ 3735]

#M._ap_JQ6 ... ... ... ... ... ... ... ... ... ... ... ... ... ... ... [ 3735]

#M._av_104 GTG GGT GCC GCC CTG CAG GCC GGT GTG CTT AAG GGC GAG GTG AAA [ 3780]

#M.ap_K10 ... ... ... ... ... ... ... ... ... ... ... ... ... ... ... [ 3780]

#M._ap_S397 ... ... ... ... ... ... ... ... ... ... ... ... ... ... ... [ 3780]

#M._ap_JQ5 ... ... ... ... ... ... ... ... ... ... ... ... ... ... ... [ 3780]

#M._ap_JQ6 ... ... ... ... ... ... ... ... ... ... ... ... ... ... ... [ 3780]

#M._av_104 GAC GTT CTG CTG CTT GAC GTT ACG CCG CTG AGC CTG GGT ATC GAG [ 3825]

#M.ap_K10 ... ... ... ... ... ... ... ... ... ... ... ... ... ... ... [ 3825]

#M._ap_S397 ... ... ... ... ... ... ... ... ... ... ... ... ... ... ... [ 3825]

#M._ap_JQ5 ... ... ... ... ... ... ... ... ... ... ... ... ... ... ... [ 3825]

#M._ap_JQ6 ... ... ... ... ... ... ... ... ... ... ... ... ... ... ... [ 3825]

#M._av_104 ACC AAG GGT GGC GTG ATG ACC AAG CTG ATC GAA CGC AAC ACC ACC [ 3870]

#M.ap_K10 ... ... ... ... ... ... ... ... ... ... ... ... ... ... ... [ 3870]

#M._ap_S397 ... ... ... ... ... ... ... ... ... ... ... ... ... ... ... [ 3870]

#M._ap_JQ5 ... ... ... ... ... ... ... ... ... ... ... ... ... ... ... [ 3870]

#M._ap_JQ6 ... ... ... ... ... ... ... ... ... ... ... ... ... ... ... [ 3870]

#M._av_104 ATC CCG ACC AAG CGG TCC GAG ACG TTC ACC ACG GCC GAC GAC AAC [ 3915]

#M.ap_K10 ... ... ... ... ... ... ... ... ... ... ... ... ... ... ... [ 3915]

#M._ap_S397 ... ... ... ... ... ... ... ... ... ... ... ... ... ... ... [ 3915]

#M._ap_JQ5 ... ... ... ... ... ... ... ... ... ... ... ... ... ... ... [ 3915]

#M._ap_JQ6 ... ... ... ... ... ... ... ... ... ... ... ... ... ... ... [ 3915]

#M._av_104 CAG CCG TCG GTG CAG ATC CAG GTG TAC CAG GGT GAG CGC GAA ATC [ 3960]

#M.ap_K10 ... ... ... ... ... ... ... ... ..T ... ... ... ... ... ... [ 3960]

#M._ap_S397 ... ... ... ... ... ... ... ... ..T ... ... ... ... ... ... [ 3960]

#M._ap_JQ5 ... ... ... ... ... ... ... ... ..T ... ... ... ... ... ... [ 3960]

#M._ap_JQ6 ... ... ... ... ... ... ... ... ..T ... ... ... ... ... ... [ 3960]

#M._av_104 GCC GCG CAC AAC AAG CTG CTC GGC TCC TTC GAG CTG ACC GGA ATC [ 4005]

#M.ap_K10 ... ... ... ... ... ... ... ... ... ... ... ... ... ... ..T [ 4005]

#M._ap_S397 ... ... ... ... ... ... ... ... ... ... ... ... ... ... ..T [ 4005]

#M._ap_JQ5 ... ... ... ... ... ... ... ... ... ... ... ... ... ... ..T [ 4005]

#M._ap_JQ6 ... ... ... ... ... ... ... ... ... ... ... ... ... ... ..T [ 4005]

#M._av_104 CCG CCG GCG CCC CGC GGC GTG CCG CAG ATC GAG GTC ACC TTC GAC [ 4050]

#M.ap_K10 ... ... ... ... ... ... ... ... ... ... ... ... ... ... ... [ 4050]

#M._ap_S397 ... ... ... ... ... ... ... ... ... ... ... ... ... ... ... [ 4050]

#M._ap_JQ5 ... ... ... ... ... ... ... ... ... ... ... ... ... ... ... [ 4050]

#M._ap_JQ6 ... ... ... ... ... ... ... ... ... ... ... ... ... ... ... [ 4050]

#M._av_104 ATC GAC GCC AAC GGC ATC GTG CAC GTC ACC GCC AAG GAC AAG GGC [ 4095]

#M.ap_K10 ... ... ... ... ... ... ... ... ... ... ... ... ... ... ... [ 4095]

#M._ap_S397 ... ... ... ... ... ... ... ... ... ... ... ... ... ... ... [ 4095]

#M._ap_JQ5 ... ... ... ... ... ... ... ... ... ... ... ... ... ... ... [ 4095]

#M._ap_JQ6 ... ... ... ... ... ... ... ... ... ... ... ... ... ... ... [ 4095]

#M._av_104 ACC GGT AAG GAG AAC ACG ATC AAG ATC CAG GAG GGC TCC GGC CTG [ 4140]

#M.ap_K10 ... ... ... ... ... ... ... ... ... ... ... ... ... ... ... [ 4140]

#M._ap_S397 ... ... ... ... ... ... ... ... ... ... ... ... ... ... ... [ 4140]

#M._ap_JQ5 ... ... ... ... ... ... ... ... ... ... ... ... ... ... ... [ 4140]

#M._ap_JQ6 ... ... ... ... ... ... ... ... ... ... ... ... ... ... ... [ 4140]

#M._av_104 TCC AAG GAG GAG ATC GAC CGG ATG ATC AAG GAC GCC GAG GCG CAC [ 4185]

#M.ap_K10 ... ... ... ... ... ... ... ... ... ... ... ... ... ... ... [ 4185]

#M._ap_S397 ... ... ... ... ... ... ... ... ... ... ... ... ... ... ... [ 4185]

#M._ap_JQ5 ... ... ... ... ... ... ... ... ... ... ... ... ... ... ... [ 4185]

#M._ap_JQ6 ... ... ... ... ... ... ... ... ... ... ... ... ... ... ... [ 4185]

#M._av_104 GCC GAG GAG GAC CGC AAG CGG CGC GAG GAA GCC GAC GTC CGC AAC [ 4230]

#M.ap_K10 ... ... ... ... ... ... A.. ... ... ... ... ... ... ... ... [ 4230]

#M._ap_S397 ... ... ... ... ... ... A.. ... ... ... ... ... ... ... ... [ 4230]

#M._ap_JQ5 ... ... ... ... ... ... A.. ... ... ... ... ... ... ... ... [ 4230]

#M._ap_JQ6 ... ... ... ... ... ... A.. ... ... ... ... ... ... ... ... [ 4230]

#M._av_104 CAA GCG GAA TCG CTT GTC TAC CAG ACG GAG AAG TTC GTC AAG GAC [ 4275]

#M.ap_K10 ... ... ... ... ... ... ... ... ... ... ... ... ... ... ... [ 4275]

#M._ap_S397 ... ... ... ... ... ... ... ... ... ... ... ... ... ... ... [ 4275]

#M._ap_JQ5 ... ... ... ... ... ... ... ... ... ... ... ... ... ... ... [ 4275]

#M._ap_JQ6 ... ... ... ... ... ... ... ... ... ... ... ... ... ... ... [ 4275]

#M._av_104 CAG CGC GAG GCC GAG GGT GGC TCG AAG GTT CCC GAG GAG ACG TTG [ 4320]

#M.ap_K10 ... ... ... ... ... ..C ... ... ... ... ... ... ... ... C.. [ 4320]

#M._ap_S397 ... ... ... ... ... ..C ... ... ... ... ... ... ... ... C.. [ 4320]

#M._ap_JQ5 ... ... ... ... ... ..C ... ... ... ... ... ... ... ... C.. [ 4320]

#M._ap_JQ6 ... ... ... ... ... ..C ... ... ... ... ... ... ... ... C.. [ 4320]

#M._av_104 TCC AAG GTC GAC GCC GCG ATC GCC GAC GCC AAG ACG GCC CTG GGC [ 4365]

#M.ap_K10 ... ... ... ... ... ... ... ... ... ... ... ... ... ... ... [ 4365]

#M._ap_S397 ... ... ... ... ... ... ... ... ... ... ... ... ... ... ... [ 4365]

#M._ap_JQ5 ... ... ... ... ... ... ... ... ... ... ... ... ... ... ... [ 4365]

#M._ap_JQ6 ... ... ... ... ... ... ... ... ... ... ... ... ... ... ... [ 4365]

#M._av_104 GGC ACC GAC ATC ACC GCG ATC AAG TCG GCG ATG GAG AAG CTC GGC [ 4410]

#M.ap_K10 ... ... ... ... ... ... ... ... ... ... ... ... ... ... ... [ 4410]

#M._ap_S397 ... ... ... ... ... ... ... ... ... ... ... ... ... ... ... [ 4410]

#M._ap_JQ5 ... ... ... ... ... ... ... ... ... ... ... ... ... ... ... [ 4410]

#M._ap_JQ6 ... ... ... ... ... ... ... ... ... ... ... ... ... ... ... [ 4410]

#M._av_104 CAG GAG TCG CAA GCG CTG GGA CAG GCA ATC TAC GAG GCC ACC CAG [ 4455]

#M.ap_K10 ... ... ... ... ... ... ... ... ... ... ... ... ... ... ... [ 4455]

#M._ap_S397 ... ... ... ... ... ... ... ... ... ... ... ... ... ... ... [ 4455]

#M._ap_JQ5 ... ... ... ... ... ... ... ... ... ... ... ... ... ... ... [ 4455]

#M._ap_JQ6 ... ... ... ... ... ... ... ... ... ... ... ... ... ... ... [ 4455]

#M._av_104 GCC GAG TCC GCC CAG GCC GGC GGG CCG GAC GGT GCC GCG GCC GGC [ 4500]

#M.ap_K10 ... ... ... ... ... ..T ... ... ... ... ... ... ... ... ... [ 4500]

#M._ap_S397 ... ... ... ... ... ..T ... ... ... ... ... ... ... ... ... [ 4500]

#M._ap_JQ5 ... ... ... ... ... ..T ... ... ... ... ... ... ... ... ... [ 4500]

#M._ap_JQ6 ... ... ... ... ... ..T ... ... ... ... ... ... ... ... ... [ 4500]

#M._av_104 GGC GGG TCC GGA TCC GCC GAC GAC GTT GTG GAC GCG GAG GTG GTC [ 4545]

#M.ap_K10 ... ... ... ... ... ... ... ..T ... ... ... ... ... ... ... [ 4545]

#M._ap_S397 ... ... ... ... ... ... ... ..T ... ... ... ... ... ... ... [ 4545]

#M._ap_JQ5 ... ... ... ... ... ... ... ..T ... ... ... ... ... ... ... [ 4545]

#M._ap_JQ6 ... ... ... ... ... ... ... ..T ... ... ... ... ... ... ... [ 4545]

#M._av_104 GAC GAT GAC CGG GAG TCC AAG TGA TTG AGA GGC GAA GAC ATG AGT [ 4590]

#M.ap_K10 ... ... ... ... ... ... ... ... --- --- --- --- --- ... ... [ 4590]

#M._ap_S397 ... ... ... ... ... ... ... ... --- --- --- --- --- ... ... [ 4590]

#M._ap_JQ5 ... ... ... ... ... ... ... ... --- --- --- --- --- ... ... [ 4590]

#M._ap_JQ6 ... ... ... ... ... ... ... ... --- --- --- --- --- ... ... [ 4590]

#M._av_104 TCG TCG GTT ACC CCG TCG CGT CCC ACC ACC GGC ACC GCC CAG ATC [ 4635]

#M.ap_K10 ... ... ... ... ... ... ... ... ... ... ... ... ... ... ... [ 4635]

#M._ap_S397 ... ... ... ... ... ... ... ... ... ... ... ... ... ... ... [ 4635]

#M._ap_JQ5 ... ... ... ... ... ... ... ... ... ... ... ... ... ... ... [ 4635]

#M._ap_JQ6 ... ... ... ... ... ... ... ... ... ... ... ... ... ... ... [ 4635]

#M._av_104 GGC GTC ACC GGG CTG GCC GTG ATG GGT TCG AAC ATC GCC CGC AAT [ 4680]

#M.ap_K10 ... ... ... ... ... ... ... ... ... ... ... ... ... ... ... [ 4680]

#M._ap_S397 ... ... ... ... ... ... ... ... ... ... ... ... ... ... ... [ 4680]

#M._ap_JQ5 ... ... ... ... ... ... ... ... ... ... ... ... ... ... ... [ 4680]

#M._ap_JQ6 ... ... ... ... ... ... ... ... ... ... ... ... ... ... ... [ 4680]

#M._av_104 TTC GCC AGG CAC GGC TAC ACC GTG GCG CTG CAC AAC CGG TCG ATC [ 4725]

#M.ap_K10 ... ... C.. ... ..T ... ... ... ... ... ... ... ... ..T ... [ 4725]

#M._ap_S397 ... ... C.. ... ..T ... ... ... ... ... ... ... ... ..T ... [ 4725]

#M._ap_JQ5 ... ... C.. ... ..T ... ... ... ... ... ... ... ... ..T ... [ 4725]

#M._ap_JQ6 ... ... C.. ... ..T ... ... ... ... ... ... ... ... ..T ... [ 4725]

#M._av_104 GCC AAA ACC GAC GCG CTG CTG AAA GAG CAC GGC GAC GAG GGC GAG [ 4770]

#M.ap_K10 ... ... ... ... ... ... ... ... ... ... ... ... ... ... A.. [ 4770]

#M._ap_S397 ... ... ... ... ... ... ... ... ... ... ... ... ... ... A.. [ 4770]

#M._ap_JQ5 ... ... ... ... ... ... ... ... ... ... ... ... ... ... A.. [ 4770]

#M._ap_JQ6 ... ... ... ... ... ... ... ... ... ... ... ... ... ... A.. [ 4770]

#M._av_104 TTC GTG CGC TGC GAA ACC ATC GCC GAA TTC CTC GAC GCG CTG GAA [ 4815]

#M.ap_K10 ... ... ... ... ... ... ... ... ... ... ... ... ... ... ... [ 4815]

#M._ap_S397 ... ... ... ... ... ... ... ... ... ... ... ... ... ... ... [ 4815]

#M._ap_JQ5 ... ... ... ... ... ... ... ... ... ... ... ... ... ... ... [ 4815]

#M._ap_JQ6 ... ... ... ... ... ... ... ... ... ... ... ... ... ... ... [ 4815]

#M._av_104 AAA CCG CGC CGC GTG CTG ATC ATG GTC AAG GCC GGC GAC CCC ACC [ 4860]

#M.ap_K10 ... ... ..T ... ... ... ..A ... ... ... ... ... ... ... ... [ 4860]

#M._ap_S397 ... ... ..T ... ... ... ..A ... ... ... ... ... ... ... ... [ 4860]

#M._ap_JQ5 ... ... ..T ... ... ... ..A ... ... ... ... ... ... ... ... [ 4860]

#M._ap_JQ6 ... ... ..T ... ... ... ..A ... ... ... ... ... ... ... ... [ 4860]

#M._av_104 GAC GCG GTG ATC AAC GAG CTC GCC GAC GCC ATG GAG CCC GGC GAC [ 4905]

#M.ap_K10 ... ... ... ... ... ... ... ... ..T ... ... ... ... ... ... [ 4905]

#M._ap_S397 ... ... ... ... ... ... ... ... ..T ... ... ... ... ... ... [ 4905]

#M._ap_JQ5 ... ... ... ... ... ... ... ... ..T ... ... ... ... ... ... [ 4905]

#M._ap_JQ6 ... ... ... ... ... ... ... ... ..T ... ... ... ... ... ... [ 4905]

#M._av_104 ATC ATC ATC GAC GGC GGC AAC GCC CTC TAC ACC GAC ACC ATC CGC [ 4950]

#M.ap_K10 ... ... ... ... ... ... ... ... ... ... ... ... ... ... ... [ 4950]

#M._ap_S397 ... ... ... ... ... ... ... ... ... ... ... ... ... ... ... [ 4950]

#M._ap_JQ5 ... ... ... ... ... ... ... ... ... ... ... ... ... ... ... [ 4950]

#M._ap_JQ6 ... ... ... ... ... ... ... ... ... ... ... ... ... ... ... [ 4950]

#M._av_104 CGG GAG CAG GCG ATG CGC GAA CGC GGC CTG CAC TTC GTC GGC GCC [ 4995]

#M.ap_K10 ... ... ... ... ... ... ... ... ... ... ... ... ..G ... ... [ 4995]

#M._ap_S397 ... ... ... ... ... ... ... ... ... ... ... ... ..G ... ... [ 4995]

#M._ap_JQ5 ... ... ... ... ... ... ... ... ... ... ... ... ..G ... ... [ 4995]

#M._ap_JQ6 ... ... ... ... ... ... ... ... ... ... ... ... ..G ... ... [ 4995]

#M._av_104 GGC ATC TCC GGC GGC GAG GAG GGC GCG CTC AAC GGC CCG TCG ATC [ 5040]

#M.ap_K10 ... ... ... ... ... ... ... ... ... ... ... ... ... ... ... [ 5040]

#M._ap_S397 ... ... ... ... ... ... ... ... ... ... ... ... ... ... ... [ 5040]

#M._ap_JQ5 ... ... ... ... ... ... ... ... ... ... ... ... ... ... ... [ 5040]

#M._ap_JQ6 ... ... ... ... ... ... ... ... ... ... ... ... ... ... ... [ 5040]

#M._av_104 ATG CCC GGC GGC CCG GCC GAG TCC TAC CGG TCG CTG GGC CCG CTG [ 5085]

#M.ap_K10 ... ... ... ... ... ... ... ... ... ... ... ... ... ... ... [ 5085]

#M._ap_S397 ... ... ... ... ... ... ... ... ... ... ... ... ... ... ... [ 5085]

#M._ap_JQ5 ... ... ... ... ... ... ... ... ... ... ... ... ... ... ... [ 5085]

#M._ap_JQ6 ... ... ... ... ... ... ... ... ... ... ... ... ... ... ... [ 5085]

#M._av_104 CTC GAG GAG ATC TCC GCG CAC GTC GAC GGG GTG CCG TGC TGC ACG [ 5130]

#M.ap_K10 ... ... ... ... ... ... ... ... ... ... ... ... ... ... ... [ 5130]

#M._ap_S397 ... ... ... ... ... ... ... ... ... ... ... ... ... ... ... [ 5130]

#M._ap_JQ5 ... ... ... ... ... ... ... ... ... ... ... ... ... ... ... [ 5130]

#M._ap_JQ6 ... ... ... ... ... ... ... ... ... ... ... ... ... ... ... [ 5130]

#M._av_104 CAC ATC GGC CCC GAC GGG GCC GGG CAC TTC GTC AAG ATG GTG CAC [ 5175]

#M.ap_K10 ... ... ... ... ... ... ... ... ... ... ... ... ... ... ... [ 5175]

#M._ap_S397 ... ... ... ... ... ... ... ... ... ... ... ... ... ... ... [ 5175]

#M._ap_JQ5 ... ... ... ... ... ... ... ... ... ... ... ... ... ... ... [ 5175]

#M._ap_JQ6 ... ... ... ... ... ... ... ... ... ... ... ... ... ... ... [ 5175]

#M._av_104 AAC GGC ATC GAG TAC TCC GAC ATG CAG CTG ATC GGT GAG GCC TAC [ 5220]

#M.ap_K10 ... ... ... ... ... ... ... ... ... ... ... ..C ... ... ... [ 5220]

#M._ap_S397 ... ... ... ... ... ... ... ... ... ... ... ..C ... ... ... [ 5220]

#M._ap_JQ5 ... ... ... ... ... ... ... ... ... ... ... ..C ... ... ... [ 5220]

#M._ap_JQ6 ... ... ... ... ... ... ... ... ... ... ... ..C ... ... ... [ 5220]

#M._av_104 CAG CTG CTG CGC GAC GCG CTG GGC AAG ACC GCC GAG CAG ATC GCC [ 5265]

#M.ap_K10 ... ... ... ... ... ... ... ... ... ... ... ... ... ... ... [ 5265]

#M._ap_S397 ... ... ... ... ... ... ... ... ... ... ... ... ... ... ... [ 5265]

#M._ap_JQ5 ... ... ... ... ... ... ... ... ... ... ... ... ... ... ... [ 5265]

#M._ap_JQ6 ... ... ... ... ... ... ... ... ... ... ... ... ... ... ... [ 5265]

#M._av_104 GAC GTG TTC GAC GAA TGG AAC TCC GGC GAC CTG GAC AGC TTC CTG [ 5310]

#M.ap_K10 ... ... ... ... ... ... ... ... ... ... ... ... ... ... ... [ 5310]

#M._ap_S397 ... ... ... ... ... ... ... ... ... ... ... ... ... ... ... [ 5310]

#M._ap_JQ5 ... ... ... ... ... ... ... ... ... ... ... ... ... ... ... [ 5310]

#M._ap_JQ6 ... ... ... ... ... ... ... ... ... ... ... ... ... ... ... [ 5310]

#M._av_104 GTC GAG ATC ACC GCG CAG GTG CTG CGC CAG ACC GAC GCC AAG ACC [ 5355]

#M.ap_K10 ... ... ... ... ... ... ... ... ... ... ... ... ... ... ... [ 5355]

#M._ap_S397 ... ... ... ... ... ... ... ... ... ... ... ... ... ... ... [ 5355]

#M._ap_JQ5 ... ... ... ... ... ... ... ... ... ... ... ... ... ... ... [ 5355]

#M._ap_JQ6 ... ... ... ... ... ... ... ... ... ... ... ... ... ... ... [ 5355]

#M._av_104 GGA AAG CCG CTC GTC GAC CTC ATC CTC GAC GAG GCC GAA CAG AAG [ 5400]

#M.ap_K10 ... ... ... ... ... ... ... ... ... ... ... ... ... ... ... [ 5400]

#M._ap_S397 ... ... ... ... ... ... ... ... ... ... ... ... ... ... ... [ 5400]

#M._ap_JQ5 ... ... ... ... ... ... ... ... ... ... ... ... ... ... ... [ 5400]

#M._ap_JQ6 ... ... ... ... ... ... ... ... ... ... ... ... ... ... ... [ 5400]

#M._av_104 GGC ACC GGC CGC TGG ACG GTC AAA TCG GCG CTG GAC CTC GGC GTG [ 5445]

#M.ap_K10 ... ... ... ... ... ... ... ... ... ... ... ... ... ... ... [ 5445]

#M._ap_S397 ... ... ... ... ... ... ... ... ... ... ... ... ... ... ... [ 5445]

#M._ap_JQ5 ... ... ... ... ... ... ... ... ... ... ... ... ... ... ... [ 5445]

#M._ap_JQ6 ... ... ... ... ... ... ... ... ... ... ... ... ... ... ... [ 5445]

#M._av_104 CCG GTG ACC GGC ATC GCC GAG GCG GTG TTC GCC CGC GCG CTG TCG [ 5490]

#M.ap_K10 ... ... ... ... ... ... ... ... ... ... ... ... ... T.. ... [ 5490]

#M._ap_S397 ... ... ... ... ... ... ... ... ... ... ... ... ... T.. ... [ 5490]

#M._ap_JQ5 ... ... ... ... ... ... ... ... ... ... ... ... ... T.. ... [ 5490]

#M._ap_JQ6 ... ... ... ... ... ... ... ... ... ... ... ... ... T.. ... [ 5490]

#M._av_104 GGC TCG GTG GCC CAG CGC AGG GCC ACC ACC GGG CTG GCG TCG GGC [ 5535]

#M.ap_K10 ... ... ... ... ... ... ... ... ... ... ... ... ... ... ... [ 5535]

#M._ap_S397 ... ... ... ... ... ... ... ... ... ... ... ... ... ... ... [ 5535]

#M._ap_JQ5 ... ... ... ... ... ... ... ... ... ... ... ... ... ... ... [ 5535]

#M._ap_JQ6 ... ... ... ... ... ... ... ... ... ... ... ... ... ... ... [ 5535]

#M._av_104 CGA CTC GGC GAA AAG CCA AGC GAT GCA GCA CAG TTC ACC GAG GAC [ 5580]

#M.ap_K10 ..G T.. ... ... ... ... ... ..C ... ... ... ... ... ... ... [ 5580]

#M._ap_S397 ..G T.. ... ... ... ... ... ..C ... ... ... ... ... ... ... [ 5580]

#M._ap_JQ5 ..G T.. ... ... ... ... ... ..C ... ... ... ... ... ... ... [ 5580]

#M._ap_JQ6 ..G T.. ... ... ... ... ... ..C ... ... ... ... ... ... ... [ 5580]

#M._av_104 ATC CGC CAG GCT TTG TAC GCG TCC AAG ATC ATC GCC TAC GCG CAG [ 5625]

#M.ap_K10 ... ... ... ..C C.. ... ... ... ... ... ... ... ... ... ... [ 5625]

#M._ap_S397 ... ... ... ..C C.. ... ... ... ... ... ... ... ... ... ... [ 5625]

#M._ap_JQ5 ... ... ... ..C C.. ... ... ... ... ... ... ... ... ... ... [ 5625]

#M._ap_JQ6 ... ... ... ..C C.. ... ... ... ... ... ... ... ... ... ... [ 5625]

#M._av_104 GGC TTC AAC CAG ATC CAG GCC GGC AGC GCC GAA TAC GGC TGG GAC [ 5670]

#M.ap_K10 ... ... ... ... ... ... ... ... ... ... ... ... ... ... ... [ 5670]

#M._ap_S397 ... ... ... ... ... ... ... ... ... ... ... ... ... ... ... [ 5670]

#M._ap_JQ5 ... ... ... ... ... ... ... ... ... ... ... ... ... ... ... [ 5670]

#M._ap_JQ6 ... ... ... ... ... ... ... ... ... ... ... ... ... ... ... [ 5670]

#M._av_104 ATC ACC CCC GGC GAC CTG GCC ACC ATC TGG CGC GGC GGC TGC ATC [ 5715]

#M.ap_K10 ... ... ... ... ... ... ... ... ... ... ... ... ... ... ... [ 5715]

#M._ap_S397 ... ... ... ... ... ... ... ... ... ... ... ... ... ... ... [ 5715]

#M._ap_JQ5 ... ... ... ... ... ... ... ... ... ... ... ... ... ... ... [ 5715]

#M._ap_JQ6 ... ... ... ... ... ... ... ... ... ... ... ... ... ... ... [ 5715]

#M._av_104 ATC CGG GCC AAG TTC CTC AAC CGG ATC AAG GAC GCC TTC GAC GAG [ 5760]

#M.ap_K10 ... ... ... ... ... ... ... ... ... ... ... ... ... ... ... [ 5760]

#M._ap_S397 ... ... ... ... ... ... ... ... ... ... ... ... ... ... ... [ 5760]

#M._ap_JQ5 ... ... ... ... ... ... ... ... ... ... ... ... ... ... ... [ 5760]

#M._ap_JQ6 ... ... ... ... ... ... ... ... ... ... ... ... ... ... ... [ 5760]

#M._av_104 AAC CCC GAC CTG CCG ACC CTG ATC GTC GCG CCG TAT TTC CGC AGC [ 5805]

#M.ap_K10 ... ... ... ... ... ... ... ... ... ... ... ... ... ... ... [ 5805]

#M._ap_S397 ... ... ... ... ... ... ... ... ... ... ... ... ... ... ... [ 5805]

#M._ap_JQ5 ... ... ... ... ... ... ... ... ... ... ... ... ... ... ... [ 5805]

#M._ap_JQ6 ... ... ... ... ... ... ... ... ... ... ... ... ... ... ... [ 5805]

#M._av_104 GCG ATC GAG GCC GCC ATC GAC GGC TGG CGC CGG GTC GTG GTC ACG [ 5850]

#M.ap_K10 ... ... ... ... ... ... ... ... ... ... ... ... ... ... ... [ 5850]

#M._ap_S397 ... ... ... ... ... ... ... ... ... ... ... ... ... ... ... [ 5850]

#M._ap_JQ5 ... ... ... ... ... ... ... ... ... ... ... ... ... ... ... [ 5850]

#M._ap_JQ6 ... ... ... ... ... ... ... ... ... ... ... ... ... ... ... [ 5850]

#M._av_104 GCC ACC CGG TTG GGC ATC CCG ATC CCC GGC TTC TCC TCG GCG CTG [ 5895]

#M.ap_K10 ... ... ... ... ... ... ... ... ... ... ... ... ... ... ... [ 5895]

#M._ap_S397 ... ... ... ... ... ... ... ... ... ... ... ... ... ... ... [ 5895]

#M._ap_JQ5 ... ... ... ... ... ... ... ... ... ... ... ... ... ... ... [ 5895]

#M._ap_JQ6 ... ... ... ... ... ... ... ... ... ... ... ... ... ... ... [ 5895]

#M._av_104 TCC TAC TAC GAC GCG CTG CGC ACC GAG CGG CTG CCC GCC GCG CTC [ 5940]

#M.ap_K10 ... ... ... ... ... ... ... ... ... ... ... ... ... ... ... [ 5940]

#M._ap_S397 ... ... ... ... ... ... ... ... ... ... ... ... ... ... ... [ 5940]

#M._ap_JQ5 ... ... ... ... ... ... ... ... ... ... ... ... ... ... ... [ 5940]

#M._ap_JQ6 ... ... ... ... ... ... ... ... ... ... ... ... ... ... ... [ 5940]

#M._av_104 ACC CAG GGT CTG CGG GAC TTC TTC GGC GCG CAC ACC TAC GGG CGC [ 5985]

#M.ap_K10 ... ... ... ... ... ... ... ... ... ... ... ... ... ... ... [ 5985]

#M._ap_S397 ... ... ... ... ... ... ... ... ... ... ... ... ... ... ... [ 5985]

#M._ap_JQ5 ... ... ... ... ... ... ... ... ... ... ... ... ... ... ... [ 5985]

#M._ap_JQ6 ... ... ... ... ... ... ... ... ... ... ... ... ... ... ... [ 5985]

#M._av_104 ATC GAC GAG GAC CCC GAC AAG CGC TTC CAC ACG CTG TGG AGC GCC [ 6030]

#M.ap_K10 ... ... ... ... ... ... ... ... ... ... ... ... ... ... ... [ 6030]

#M._ap_S397 ... ... ... ... ... ... ... ... ... ... ... ... ... ... ... [ 6030]

#M._ap_JQ5 ... ... ... ... ... ... ... ... ... ... ... ... ... ... ... [ 6030]

#M._ap_JQ6 ... ... ... ... ... ... ... ... ... ... ... ... ... ... ... [ 6030]

#M._av_104 GAC CGC CGC GAA GTG CCG GCG TAA ATG AGC AAG ATC ATT GAG TAC [ 6075]

#M.ap_K10 ... ... ... ... ... ... ... ... ... ... ... ... ... ... ... [ 6075]

#M._ap_S397 ... ... ... ... ... ... ... ... ... ... ... ... ... ... ... [ 6075]

#M._ap_JQ5 ... ... ... ... ... ... ... ... ... ... ... ... ... ... ... [ 6075]

#M._ap_JQ6 ... ... ... ... ... ... ... ... ... ... ... ... ... ... ... [ 6075]

#M._av_104 GAC GAG ACC GCG CGC CGC GCC ATC GAG GCC GGC GTC AAC ACG CTT [ 6120]

#M.ap_K10 ... ... ... ... ... ... ... ... ... ... ... ... ... ... ... [ 6120]

#M._ap_S397 ... ... ... ... ... ... ... ... ... ... ... ... ... ... ... [ 6120]

#M._ap_JQ5 ... ... ... ... ... ... ... ... ... ... ... ... ... ... ... [ 6120]

#M._ap_JQ6 ... ... ... ... ... ... ... ... ... ... ... ... ... ... ... [ 6120]

#M._av_104 GCC GAC GCG GTC CGG GTG ACG CTG GGT CCG CGG GGC CGG CAT GTG [ 6165]

#M.ap_K10 ... ..T ... ... ... ... ... ... ... ... ... ... ... ... ... [ 6165]

#M._ap_S397 ... ..T ... ... ... ... ... ... ... ... ... ... ... ... ... [ 6165]

#M._ap_JQ5 ... ..T ... ... ... ... ... ... ... ... ... ... ... ... ... [ 6165]

#M._ap_JQ6 ... ..T ... ... ... ... ... ... ... ... ... ... ... ... ... [ 6165]

#M._av_104 GTG CTG GCC AAG GCA TTC GGC GGC CCG GCC GTC ACC AAC GAC GGC [ 6210]

#M.ap_K10 ... ... ... ... ... ... ... ..G ... ... ... ... ... ... ... [ 6210]

#M._ap_S397 ... ... ... ... ... ... ... ..G ... ... ... ... ... ... ... [ 6210]

#M._ap_JQ5 ... ... ... ... ... ... ... ..G ... ... ... ... ... ... ... [ 6210]

#M._ap_JQ6 ... ... ... ... ... ... ... ..G ... ... ... ... ... ... ... [ 6210]

#M._av_104 GTC ACC GTC GCG CGG GAA ATC GAC CTG GAG GAC CCG TTC GAG AAC [ 6255]

#M.ap_K10 ... ... ... ... ... ... ... ... ... ... ... ... ... ... ... [ 6255]

#M._ap_S397 ... ... ... ... ... ... ... ... ... ... ... ... ... ... ... [ 6255]

#M._ap_JQ5 ... ... ... ... ... ... ... ... ... ... ... ... ... ... ... [ 6255]

#M._ap_JQ6 ... ... ... ... ... ... ... ... ... ... ... ... ... ... ... [ 6255]

#M._av_104 CTG GGC GCC CAG CTG GTG AAG TCG GTG GCG ACC AAG ACC AAC GAC [ 6300]

#M.ap_K10 ... ... ... ... ... ... ... ... ... ... ... ... ..T ... ... [ 6300]

#M._ap_S397 ... ... ... ... ... ... ... ... ... ... ... ... ..T ... ... [ 6300]

#M._ap_JQ5 ... ... ... ... ... ... ... ... ... ... ... ... ..T ... ... [ 6300]

#M._ap_JQ6 ... ... ... ... ... ... ... ... ... ... ... ... ..T ... ... [ 6300]

#M._av_104 GTC GCC GGC GAC GGC ACC ACC ACC GCG ACC GTG CTG GCG CAG GCG [ 6345]

#M.ap_K10 ... ... ... ... ... ... ... ... ... ... ... ... ... ... ... [ 6345]

#M._ap_S397 ... ... ... ... ... ... ... ... ... ... ... ... ... ... ... [ 6345]

#M._ap_JQ5 ... ... ... ... ... ... ... ... ... ... ... ... ... ... ... [ 6345]

#M._ap_JQ6 ... ... ... ... ... ... ... ... ... ... ... ... ... ... ... [ 6345]

#M._av_104 CTG GTG AAG GGC GGC CTG CGG CTG GTG GCG GCG GGC GCC AAC CCC [ 6390]

#M.ap_K10 ... ... ... ... ... ... ... ... ... ... ..C ... ... ... ... [ 6390]

#M._ap_S397 ... ... ... ... ... ... ... ... ... ... ..C ... ... ... ... [ 6390]

#M._ap_JQ5 ... ... ... ... ... ... ... ... ... ... ..C ... ... ... ... [ 6390]

#M._ap_JQ6 ... ... ... ... ... ... ... ... ... ... ..C ... ... ... ... [ 6390]

#M._av_104 ATC GAG CTC GGT GCC GGA ATC TCC AAG GCC GCC GAC GCG GTG TCC [ 6435]

#M.ap_K10 ... ... ... ..C ... ... ... ... ... ... ... ... ... ... ... [ 6435]

#M._ap_S397 ... ... ... ..C ... ... ... ... ... ... ... ... ... ... ... [ 6435]

#M._ap_JQ5 ... ... ... ..C ... ... ... ... ... ... ... ... ... ... ... [ 6435]

#M._ap_JQ6 ... ... ... ..C ... ... ... ... ... ... ... ... ... ... ... [ 6435]

#M._av_104 GAG GCG CTG CTG GCC TCG GCC ACC ACG GTG TCC GGC AAG GAC GCG [ 6480]

#M.ap_K10 ... ... ... ... ... ... ... ... C.. ... ... ... ... ... ... [ 6480]

#M._ap_S397 ... ... ... ... ... ... ... ... C.. ... ... ... ... ... ... [ 6480]

#M._ap_JQ5 ... ... ... ... ... ... ... ... C.. ... ... ... ... ... ... [ 6480]

#M._ap_JQ6 ... ... ... ... ... ... ... ... C.. ... ... ... ... ... ... [ 6480]

#M._av_104 ATC GCC CAG GTG GCG ACC GTG TCG TCG CGC GAC CAG GTG CTC GGC [ 6525]

#M.ap_K10 ... ... ... ... ... ... ... ... ... ..T ... ... ... ... ... [ 6525]

#M._ap_S397 ... ... ... ... ... ... ... ... ... ..T ... ... ... ... ... [ 6525]

#M._ap_JQ5 ... ... ... ... ... ... ... ... ... ..T ... ... ... ... ... [ 6525]

#M._ap_JQ6 ... ... ... ... ... ... ... ... ... ..T ... ... ... ... ... [ 6525]

#M._av_104 GAG CTG GTC GGC GAG GCG ATG ACC AAG GTC GGG GTC GAC GGC GTG [ 6570]

#M.ap_K10 ... ... ... ... ... ... ... ... ... ... ... ... ... ... ... [ 6570]

#M._ap_S397 ... ... ... ... ... ... ... ... ... ... ... ... ... ... ... [ 6570]

#M._ap_JQ5 ... ... ... ... ... ... ... ... ... ... ... ... ... ... ... [ 6570]

#M._ap_JQ6 ... ... ... ... ... ... ... ... ... ... ... ... ... ... ... [ 6570]

#M._av_104 GTC AGC GTC GAA GAG TCC TCG ACG CTG AAC ACC GAG CTG GAG TTC [ 6615]

#M.ap_K10 ... ... ... ... ... ... ... ... ... ... ... ... ... ... ... [ 6615]

#M._ap_S397 ... ... ... ... ... ... ... ... ... ... ... ... ... ... ... [ 6615]

#M._ap_JQ5 ... ... ... ... ... ... ... ... ... ... ... ... ... ... ... [ 6615]

#M._ap_JQ6 ... ... ... ... ... ... ... ... ... ... ... ... ... ... ... [ 6615]

#M._av_104 ACC GAG GGC GTC GGC TTC GAC AAG GGC TTC CTG TCG GCC TAC TTC [ 6660]

#M.ap_K10 ... ... ... ... ... ... ... ... ... ... ... ... ... ... ... [ 6660]

#M._ap_S397 ... ... ... ... ... ... ... ... ... ... ... ... ... ... ... [ 6660]

#M._ap_JQ5 ... ... ... ... ... ... ... ... ... ... ... ... ... ... ... [ 6660]

#M._ap_JQ6 ... ... ... ... ... ... ... ... ... ... ... ... ... ... ... [ 6660]

#M._av_104 GTC ACC GAC TTC GAC GCC CAG CAG GCC GTG CTG GAT GAC CCG GTG [ 6705]

#M.ap_K10 ... ... ... ... ... ... ... ... ... ... ... ... ... ... ... [ 6705]

#M._ap_S397 ... ... ... ... ... ... ... ... ... ... ... ... ... ... ... [ 6705]

#M._ap_JQ5 ... ... ... ... ... ... ... ... ... ... ... ... ... ... ... [ 6705]

#M._ap_JQ6 ... ... ... ... ... ... ... ... ... ... ... ... ... ... ... [ 6705]

#M._av_104 ATC CTG TTG CAC CAG GAG AAG ATC AGC TCG CTG CCC GAC CTG CTG [ 6750]

#M.ap_K10 ... ... ... ... ... ... ... ... ... ... ... ... ... ... ... [ 6750]

#M._ap_S397 ... ... ... ... ... ... ... ... ... ... ... ... ... ... ... [ 6750]

#M._ap_JQ5 ... ... ... ... ... ... ... ... ... ... ... ... ... ... ... [ 6750]

#M._ap_JQ6 ... ... ... ... ... ... ... ... ... ... ... ... ... ... ... [ 6750]

#M._av_104 CCG ATG CTG GAG AAG GTC GCC GAG TCG GGC AAA CCG CTG CTG ATC [ 6795]

#M.ap_K10 ... ... ... ... ... ... ... ... ... ... ... ... ... ... ... [ 6795]

#M._ap_S397 ... ... ... ... ... ... ... ... ... ... ... ... ... ... ... [ 6795]

#M._ap_JQ5 ... ... ... ... ... ... ... ... ... ... ... ... ... ... ... [ 6795]

#M._ap_JQ6 ... ... ... ... ... ... ... ... ... ... ... ... ... ... ... [ 6795]

#M._av_104 ATC GCC GAG GAC ATC GAG GGC GAG GCG CTG GCC ACC CTC GTC GTC [ 6840]

#M.ap_K10 ... ... ... ... ... ... ... ... ... ... ... ... ... ... ... [ 6840]

#M._ap_S397 ... ... ... ... ... ... ... ... ... ... ... ... ... ... ... [ 6840]

#M._ap_JQ5 ... ... ... ... ... ... ... ... ... ... ... ... ... ... ... [ 6840]

#M._ap_JQ6 ... ... ... ... ... ... ... ... ... ... ... ... ... ... ... [ 6840]

#M._av_104 AAC TCC ATT CGC AAG ACG CTG AAA GCC GTT GCG GTC AAG GCG CCG [ 6885]

#M.ap_K10 ... ... ... ... ... ... ... ... ... ... ... ... ... ... ... [ 6885]

#M._ap_S397 ... ... ... ... ... ... ... ... ... ... ... ... ... ... ... [ 6885]

#M._ap_JQ5 ... ... ... ... ... ... ... ... ... ... ... ... ... ... ... [ 6885]

#M._ap_JQ6 ... ... ... ... ... ... ... ... ... ... ... ... ... ... ... [ 6885]

#M._av_104 TTC TTC GGC GAC CGG CGC AAG GCG TTC CTG GAG GAC CTG GCC ATC [ 6930]

#M.ap_K10 ... ... ... ... ... ... ... ... ... ... ... ... ... ... ... [ 6930]

#M._ap_S397 ... ... ... ... ... ... ... ... ... ... ... ... ... ... ... [ 6930]

#M._ap_JQ5 ... ... ... ... ... ... ... ... ... ... ... ... ... ... ... [ 6930]

#M._ap_JQ6 ... ... ... ... ... ... ... ... ... ... ... ... ... ... ... [ 6930]

#M._av_104 GTG ACC GGC GGC CAG GTG ATC AAC CCC GAC ACC GGC CTG CTG CTG [ 6975]

#M.ap_K10 ... ... ... ..G ... ... ... ... ... ... ... ... ... ... ... [ 6975]

#M._ap_S397 ... ... ... ..G ... ... ... ... ... ... ... ... ... ... ... [ 6975]

#M._ap_JQ5 ... ... ... ..G ... ... ... ... ... ... ... ... ... ... ... [ 6975]

#M._ap_JQ6 ... ... ... ..G ... ... ... ... ... ... ... ... ... ... ... [ 6975]

#M._av_104 CGC GAG GTC GGC ACC GAG GTG CTC GGC TCG GCC CGG CGG GTG GTG [ 7020]

#M.ap_K10 ... ... ... ... ... ... ... ... ... ... ... ... ... ... ... [ 7020]

#M._ap_S397 ... ... ... ... ... ... ... ... ... ... ... ... ... ... ... [ 7020]

#M._ap_JQ5 ... ... ... ... ... ... ... ... ... ... ... ... ... ... ... [ 7020]

#M._ap_JQ6 ... ... ... ... ... ... ... ... ... ... ... ... ... ... ... [ 7020]

#M._av_104 GTC AGC AAG GAC GAC ACC ATC ATC GTC GAC GGC GGC GGC GCC AAA [ 7065]

#M.ap_K10 ... ... ... ... ... ... ... ... ... ... ... ... ... ... ... [ 7065]

#M._ap_S397 ... ... ... ... ... ... ... ... ... ... ... ... ... ... ... [ 7065]

#M._ap_JQ5 ... ... ... ... ... ... ... ... ... ... ... ... ... ... ... [ 7065]

#M._ap_JQ6 ... ... ... ... ... ... ... ... ... ... ... ... ... ... ... [ 7065]

#M._av_104 GAC GCC GTG GCC AAT CGG ATC AAG CAG CTG CGC GCC GAG ATC GAG [ 7110]

#M.ap_K10 ... ... ... ... ..C ... ... ... ... ... ... ... ... ... ... [ 7110]

#M._ap_S397 ... ... ... ... ..C ... ... ... ... ... ... ... ... ... ... [ 7110]

#M._ap_JQ5 ... ... ... ... ..C ... ... ... ... ... ... ... ... ... ... [ 7110]

#M._ap_JQ6 ... ... ... ... ..C ... ... ... ... ... ... ... ... ... ... [ 7110]

#M._av_104 AAG ACC GAC TCC GAC TGG GAC CGC GAG AAG CTG CAG GAG CGG CTG [ 7155]

#M.ap_K10 ... ... ... ... ... ... ... ... ... ... ... ... ... ... ... [ 7155]

#M._ap_S397 ... ... ... ... ... ... ... ... ... ... ... ... ... ... ... [ 7155]

#M._ap_JQ5 ... ... ... ... ... ... ... ... ... ... ... ... ... ... ... [ 7155]

#M._ap_JQ6 ... ... ... ... ... ... ... ... ... ... ... ... ... ... ... [ 7155]

#M._av_104 GCC AAG CTG GCC GGC GGC GTG GCG GTG ATC AAG GTC GGT GCG GCC [ 7200]

#M.ap_K10 ... ... ... ... ... ... ... ... ... ... ... ... ... ... ... [ 7200]

#M._ap_S397 ... ... ... ... ... ... ... ... ... ... ... ... ... ... ... [ 7200]

#M._ap_JQ5 ... ... ... ... ... ... ... ... ... ... ... ... ... ... ... [ 7200]

#M._ap_JQ6 ... ... ... ... ... ... ... ... ... ... ... ... ... ... ... [ 7200]

#M._av_104 ACC GAG ACC GCG CTC AAG GAA CGC AAG GAG AGC GTC GAG GAC GCG [ 7245]

#M.ap_K10 ... ... ... ... ... ... ... ... ... ..A ... ... ... ... ... [ 7245]

#M._ap_S397 ... ... ... ... ... ... ... ... ... ..A ... ... ... ... ... [ 7245]

#M._ap_JQ5 ... ... ... ... ... ... ... ... ... ..A ... ... ... ... ... [ 7245]

#M._ap_JQ6 ... ... ... ... ... ... ... ... ... ..A ... ... ... ... ... [ 7245]

#M._av_104 GTG GCG GCC GCC AAG GCC GCC GTC GAG GAG GGC ATC GTC GCC GGT [ 7290]

#M.ap_K10 ... ..C ... ... ... ... ..G ... ... ... ... ... ... ... ..C [ 7290]

#M._ap_S397 ... ..C ... ... ... ... ..G ... ... ... ... ... ... ... ..C [ 7290]

#M._ap_JQ5 ... ..C ... ... ... ... ..G ... ... ... ... ... ... ... ..C [ 7290]

#M._ap_JQ6 ... ..C ... ... ... ... ..G ... ... ... ... ... ... ... ..C [ 7290]

#M._av_104 GGC GGG TCG GCG CTG CTG CAG GCC CGT AAG GCC CTC GAC GAG CTG [ 7335]

#M.ap_K10 ... ... ... ... ... ... ... ... ..C ... ... ... ... ... ... [ 7335]

#M._ap_S397 ... ... ... ... ... ... ... ... ..C ... ... ... ... ... ... [ 7335]

#M._ap_JQ5 ... ... ... ... ... ... ... ... ..C ... ... ... ... ... ... [ 7335]

#M._ap_JQ6 ... ... ... ... ... ... ... ... ..C ... ... ... ... ... ... [ 7335]

#M._av_104 CGC GGA TCG CTG AGC GGC GAT CAG GCC CTG GGC GTC GAC GTC TTC [ 7380]

#M.ap_K10 ... ... ... ... ... ... ... ... ..G ... ... ... ... ... ... [ 7380]

#M._ap_S397 ... ... ... ... ... ... ... ... ..G ... ... ... ... ... ... [ 7380]

#M._ap_JQ5 ... ... ... ... ... ... ... ... ..G ... ... ... ... ... ... [ 7380]

#M._ap_JQ6 ... ... ... ... ... ... ... ... ..G ... ... ... ... ... ... [ 7380]

#M._av_104 GCC GAG GCG CTG GGG GCG CCG CTG TAC TGG ATC GCC AGC AAC GCC [ 7425]

#M.ap_K10 ... ... ... ... ... ... ... ... ... ... ... ... ... ... ... [ 7425]

#M._ap_S397 ... ... ... ... ... ... ... ... ... ... ... ... ... ... ... [ 7425]

#M._ap_JQ5 ... ... ... ... ... ... ... ... ... ... ... ... ... ... ... [ 7425]

#M._ap_JQ6 ... ... ... ... ... ... ... ... ... ... ... ... ... ... ... [ 7425]

#M._av_104 GGG CTG GAC GGC GCG GTG GCC GTG CAC AAG GTC GCC GAG CTG CCC [ 7470]

#M.ap_K10 ... ... ... ... ... ... ... ... ... ... ... ... ... ... ... [ 7470]

#M._ap_S397 ... ... ... ... ... ... ... ... ... ... ... ... ... ... ... [ 7470]

#M._ap_JQ5 ... ... ... ... ... ... ... ... ... ... ... ... ... ... ... [ 7470]

#M._ap_JQ6 ... ... ... ... ... ... ... ... ... ... ... ... ... ... ... [ 7470]

#M._av_104 GCC GGT CAC GGG CTC AAC GCC GAG AAG CTC AGC TAC GGC GAC CTG [ 7515]

#M.ap_K10 ... ..C ... ... ... ... ... ... ... ... ... ... ... ... ... [ 7515]

#M._ap_S397 ... ..C ... ... ... ... ... ... ... ... ... ... ... ... ... [ 7515]

#M._ap_JQ5 ... ..C ... ... ... ... ... ... ... ... ... ... ... ... ... [ 7515]

#M._ap_JQ6 ... ..C ... ... ... ... ... ... ... ... ... ... ... ... ... [ 7515]

#M._av_104 ATC GCC GAC GGC GTC ATC GAC CCG GTC AAG GTG ACC CGC TCG GCG [ 7560]

#M.ap_K10 ... ... ... ... ... ... ... ... ... ... ... ... ... ... ... [ 7560]

#M._ap_S397 ... ... ... ... ... ... ... ... ... ... ... ... ... ... ... [ 7560]

#M._ap_JQ5 ... ... ... ... ... ... ... ... ... ... ... ... ... ... ... [ 7560]

#M._ap_JQ6 ... ... ... ... ... ... ... ... ... ... ... ... ... ... ... [ 7560]

#M._av_104 GTG CTC AAC TCG GCC TCG GTG GCG CGG ATG GTG CTC ACC ACC GAG [ 7605]

#M.ap_K10 ... ... ... ... ... ... ... ... ... ... ... ... ... ... ... [ 7605]

#M._ap_S397 ... ... ... ... ... ... ... ... ... ... ... ... ... ... ... [ 7605]

#M._ap_JQ5 ... ... ... ... ... ... ... ... ... ... ... ... ... ... ... [ 7605]

#M._ap_JQ6 ... ... ... ... ... ... ... ... ... ... ... ... ... ... ... [ 7605]

#M._av_104 ACG GCG GTT GTC GAC AAG CCG GCC GAG GAG GCC GAC GAC CAC GGC [ 7650]

#M.ap_K10 ... ... ... ... ... ... ... ... ... ... ... ... ... ... ... [ 7650]

#M._ap_S397 ... ... ... ... ... ... ... ... ... ... ... ... ... ... ... [ 7650]

#M._ap_JQ5 ... ... ... ... ... ... ... ... ... ... ... ... ... ... ... [ 7650]

#M._ap_JQ6 ... ... ... ... ... ... ... ... ... ... ... ... ... ... ... [ 7650]

#M._av_104 CAC GGT CAT CAC CAT CAC TGA ATG GCC AAG ACA ATT GCG TAC GAC [ 7695]

#M.ap_K10 ... ..A ... ... ... ... .AG ... ... ... ... ... ... ... ... [ 7695]

#M._ap_S397 ... ..A ... ... ... ... .AG ... ... ... ... ... ... ... ... [ 7695]

#M._ap_JQ5 ... ..A ... ... ... ... .AG ... ... ... ... ... ... ... ... [ 7695]

#M._ap_JQ6 ... ..A ... ... ... ... .AG ... ... ... ... ... ... ... ... [ 7695]

#M._av_104 GAA GAG GCC CGT CGC GGC CTC GAG CGG GGG CTC AAC GCC CTC GCC [ 7740]

#M.ap_K10 ... ... ... ... ... ... ... ... ... ... ... ... ... ... ... [ 7740]

#M._ap_S397 ... ... ... ... ... ... ... ... ... ... ... ... ... ... ... [ 7740]

#M._ap_JQ5 ... ... ... ... ... ... ... ... ... ... ... ... ... ... ... [ 7740]

#M._ap_JQ6 ... ... ... ... ... ... ... ... ... ... ... ... ... ... ... [ 7740]

#M._av_104 GAC GCG GTA AAG GTC ACG TTG GGC CCC AAG GGT CGC AAC GTC GTC [ 7785]

#M.ap_K10 ... ... ... ... ... ... ... ... ... ... ... ... ... ... ... [ 7785]

#M._ap_S397 ... ... ... ... ... ... ... ... ... ... ... ... ... ... ... [ 7785]

#M._ap_JQ5 ... ... ... ... ... ... ... ... ... ... ... ... ... ... ... [ 7785]

#M._ap_JQ6 ... ... ... ... ... ... ... ... ... ... ... ... ... ... ... [ 7785]

#M._av_104 CTG GAG AAG AAG TGG GGT GCC CCC ACG ATC ACC AAC GAT GGT GTG [ 7830]

#M.ap_K10 ... ... ... ... ... ... ... ... ... ... ... ... ... ... ... [ 7830]

#M._ap_S397 ... ... ... ... ... ... ... ... ... ... ... ... ... ... ... [ 7830]

#M._ap_JQ5 ... ... ... ... ... ... ... ... ... ... ... ... ... ... ... [ 7830]

#M._ap_JQ6 ... ... ... ... ... ... ... ... ... ... ... ... ... ... ... [ 7830]

#M._av_104 TCC ATC GCC AAG GAG ATC GAG CTG GAG GAC CCG TAC GAG AAG ATC [ 7875]

#M.ap_K10 ... ... ... ... ... ... ... ... ... ... ... ... ... ... ... [ 7875]

#M._ap_S397 ... ... ... ... ... ... ... ... ... ... ... ... ... ... ... [ 7875]

#M._ap_JQ5 ... ... ... ... ... ... ... ... ... ... ... ... ... ... ... [ 7875]

#M._ap_JQ6 ... ... ... ... ... ... ... ... ... ... ... ... ... ... ... [ 7875]

#M._av_104 GGC GCC GAG CTG GTC AAG GAA GTC GCC AAG AAG ACC GAC GAC GTC [ 7920]

#M.ap_K10 ... ... ... ... ... ... ... ... ... ... ... ... ... ... ... [ 7920]

#M._ap_S397 ... ... ... ... ... ... ... ... ... ... ... ..T ... ... ... [ 7920]

#M._ap_JQ5 ... ... ... ... ... ... ... ... ... ... ... ..T ... ... ... [ 7920]

#M._ap_JQ6 ... ... ... ... ... ... ... ... ... ... ... ..T ... ... ... [ 7920]

#M._av_104 GCC GGT GAC GGC ACG ACG ACG GCC ACG GTG CTC GCC CAG GCG TTG [ 7965]

#M.ap_K10 ... ... ... ... ... ... ... ... ... ... ... ... ... ... ... [ 7965]

#M._ap_S397 ... ... ... ... ... ... ... ... ... ... ... ... ... ... ... [ 7965]

#M._ap_JQ5 ... ... ... ... ... ... ... ... ... ... ... ... ... ... ... [ 7965]

#M._ap_JQ6 ... ... ... ... ... ... ... ... ... ... ... ... ... ... ... [ 7965]

#M._av_104 GTC CGC GAG GGC CTG CGC AAC GTC GCG GCC GGC GCC AAC CCG CTG [ 8010]

#M.ap_K10 ... ... ... ... ... ... ... ... ... ... ... ... ... ... ... [ 8010]

#M._ap_S397 ... ... ... ... ... ... ... ... ... ... ... ... ... ... ... [ 8010]

#M._ap_JQ5 ... ... ... ... ... ... ... ... ... ... ... ... ... ... ... [ 8010]

#M._ap_JQ6 ... ... ... ... ... ... ... ... ... ... ... ... ... ... ... [ 8010]

#M._av_104 GGT CTC AAG CGC GGC ATC GAG AAG GCC GTC GAG AAG GTC ACC GAG [ 8055]

#M.ap_K10 ... ... ... ... ... ... ... ... ... ... ... ... ... ... ... [ 8055]

#M._ap_S397 ... ... ... ... ... ... ... ... ... ... ... ... ... ... ... [ 8055]

#M._ap_JQ5 ... ... ... ... ... ... ... ... ... ... ... ... ... ... ... [ 8055]

#M._ap_JQ6 ... ... ... ... ... ... ... ... ... ... ... ... ... ... ... [ 8055]

#M._av_104 ACC CTG CTC AAG TCG GCC AAG GAG GTC GAG ACC AAG GAC CAG ATC [ 8100]

#M.ap_K10 ... ... ... ... ... ... ... ... ... ... ... ... ... ... ... [ 8100]

#M._ap_S397 ... ... ... ... ... ... ... ... ... ... ... ... ... ... ... [ 8100]

#M._ap_JQ5 ... ... ... ... ... ... ... ... ... ... ... ... ... ... ... [ 8100]

#M._ap_JQ6 ... ... ... ... ... ... ... ... ... ... ... ... ... ... ... [ 8100]

#M._av_104 GCT GCC ACC GCG GCC ATC TCC GCG GGC GAC CAG TCG ATC GGC GAC [ 8145]

#M.ap_K10 ... ... ... ... ... ... ... ... ... ... ... ... ... ... ... [ 8145]

#M._ap_S397 ... ... ... ... ... ... ... ... ... ... ... ... ... ... ... [ 8145]

#M._ap_JQ5 ... ... ... ... ... ... ... ... ... ... ... ... ... ... ... [ 8145]

#M._ap_JQ6 ... ... ... ... ... ... ... ... ... ... ... ... ... ... ... [ 8145]

#M._av_104 CTG ATC GCC GAG GCG ATG GAC AAG GTC GGC AAC GAG GGC GTC ATC [ 8190]

#M.ap_K10 ... ... ... ... ... ... ... ... ... ... ... ... ... ... ... [ 8190]

#M._ap_S397 ... ... ... ... ... ... ... ... ... ... ... ... ... ... ... [ 8190]

#M._ap_JQ5 ... ... ... ... ... ... ... ... ... ... ... ... ... ... ... [ 8190]

#M._ap_JQ6 ... ... ... ... ... ... ... ... ... ... ... ... ... ... ... [ 8190]

#M._av_104 ACC GTC GAG GAG TCC AAC ACC TTC GGC CTG CAG CTC GAG CTC ACC [ 8235]

#M.ap_K10 ... ... ... ... ... ... ... ... ... ... ... ... ... ... ... [ 8235]

#M._ap_S397 ... ... ... ... ... ... ... ... ... ... ... ... ... ... ... [ 8235]

#M._ap_JQ5 ... ... ... ... ... ... ... ... ... ... ... ... ... ... ... [ 8235]

#M._ap_JQ6 ... ... ... ... ... ... ... ... ... ... ... ... ... ... ... [ 8235]

#M._av_104 GAG GGT ATG CGG TTC GAC AAG GGT TAC ATC TCG GGC TAC TTC GTC [ 8280]

#M.ap_K10 ... ... ... ... ... ... ... ... ... ... ... ... ... ... ... [ 8280]

#M._ap_S397 ... ... ... ... ... ... ... ... ... ... ... ... ... ... ... [ 8280]

#M._ap_JQ5 ... ... ... ... ... ... ... ... ... ... ... ... ... ... ... [ 8280]

#M._ap_JQ6 ... ... ... ... ... ... ... ... ... ... ... ... ... ... ... [ 8280]

#M._av_104 ACC GAC GCC GAG CGT CAG GAA GCC GTC CTC GAG GAC CCG TTC ATC [ 8325]

#M.ap_K10 ..G ... ... ... ... ... ... ..G ... ... ... ... ... ... ... [ 8325]

#M._ap_S397 ..G ... ... ... ... ... ... ..G ... ... ... ... ... ... ... [ 8325]

#M._ap_JQ5 ..G ... ... ... ... ... ... ..G ... ... ... ... ... ... ... [ 8325]

#M._ap_JQ6 ..G ... ... ... ... ... ... ..G ... ... ... ... ... ... ... [ 8325]

#M._av_104 CTG CTG GTC AGC TCC AAG GTC TCG ACC GTC AAG GAC CTG CTG CCG [ 8370]

#M.ap_K10 ... ... ... ... ... ... ... ... ... ... ... ... ... ... ... [ 8370]

#M._ap_S397 ... ... ... ... ... ... ... ... ... ... ... ... ... ... ... [ 8370]

#M._ap_JQ5 ... ... ... ... ... ... ... ... ... ... ... ... ... ... ... [ 8370]

#M._ap_JQ6 ... ... ... ... ... ... ... ... ... ... ... ... ... ... ... [ 8370]

#M._av_104 CTG CTG GAG AAG GTC ATC CAG GCC GGC AAG CCG CTG CTG ATC ATC [ 8415]

#M.ap_K10 ... ... ... ... ... ... ... ... ... ... ... ... ... ... ... [ 8415]

#M._ap_S397 ... ... ... ... ... ... ... ... ... ... ... ... ... ... ... [ 8415]

#M._ap_JQ5 ... ... ... ... ... ... ... ... ... ... ... ... ... ... ... [ 8415]

#M._ap_JQ6 ... ... ... ... ... ... ... ... ... ... ... ... ... ... ... [ 8415]

#M._av_104 GCC GAG GAC GTC GAG GGC GAG GCC CTG TCC ACC CTG GTC GTC AAC [ 8460]

#M.ap_K10 ... ... ... ... ... ... ... ... ... ... ... ... ... ... ... [ 8460]

#M._ap_S397 ... ... ... ... ... ... ... ... ... ... ... ... ... ... ... [ 8460]

#M._ap_JQ5 ... ... ... ... ... ... ... ... ... ... ... ... ... ... ... [ 8460]

#M._ap_JQ6 ... ... ... ... ... ... ... ... ... ... ... ... ... ... ... [ 8460]

#M._av_104 AAG ATC CGC GGC ACC TTC AAG TCG GTG GCC GTC AAG GCG CCC GGC [ 8505]

#M.ap_K10 ... ... ... ... ... ... ... ... ... ... ... ... ... ... ... [ 8505]

#M._ap_S397 ... ... ... ... ... ... ... ... ... ... ... ... ... ... ... [ 8505]

#M._ap_JQ5 ... ... ... ... ... ... ... ... ... ... ... ... ... ... ... [ 8505]

#M._ap_JQ6 ... ... ... ... ... ... ... ... ... ... ... ... ... ... ... [ 8505]

#M._av_104 TTC GGC GAC CGC CGC AAG GCG ATG CTG CAG GAC ATG GCC ATC CTC [ 8550]

#M.ap_K10 ... ... ... ... ... ... ... ... ..T ... ... ... ... ... ... [ 8550]

#M._ap_S397 ... ... ... ... ... ... ... ... ..T ... ... ... ... ... ... [ 8550]

#M._ap_JQ5 ... ... ... ... ... ... ... ... ..T ... ... ... ... ... ... [ 8550]

#M._ap_JQ6 ... ... ... ... ... ... ... ... ..T ... ... ... ... ... ... [ 8550]

#M._av_104 ACC GGC GGC CAG GTC ATC AGC GAA GAG GTC GGC CTG TCG CTG GAG [ 8595]

#M.ap_K10 ... ... ... ... ... ... ... ... ... ... ... ... ... ... ... [ 8595]

#M._ap_S397 ... ... ... ... ... ... ... ... ... ... ... ... ... ... ... [ 8595]

#M._ap_JQ5 ... ... ... ... ... ... ... ... ... ... ... ... ... ... ... [ 8595]

#M._ap_JQ6 ... ... ... ... ... ... ... ... ... ... ... ... ... ... ... [ 8595]

#M._av_104 AGC GCC GAC ATC TCG CTG CTC GGT AAG GCC CGC AAG GTC GTC GTC [ 8640]

#M.ap_K10 ... ... ... ... ... ... ... ... ... ... ... ... ... ... ... [ 8640]

#M._ap_S397 ... ... ... ... ... ... ... ... ... ... ... ... ... ... ... [ 8640]

#M._ap_JQ5 ... ... ... ... ... ... ... ... ... ... ... ... ... ... ... [ 8640]

#M._ap_JQ6 ... ... ... ... ... ... ... ... ... ... ... ... ... ... ... [ 8640]

#M._av_104 ACC AAG GAC GAG ACC ACC ATC GTC GAG GGC GCC GGT GAC TCC GAC [ 8685]

#M.ap_K10 ... ... ... ... ... ... ... ... ... ... ... ... ... ... ... [ 8685]

#M._ap_S397 ... ... ... ... ... ... ... ... ... ... ... ... ... ... ... [ 8685]

#M._ap_JQ5 ... ... ... ... ... ... ... ... ... ... ... ... ... ... ... [ 8685]

#M._ap_JQ6 ... ... ... ... ... ... ... ... ... ... ... ... ... ... ... [ 8685]

#M._av_104 GCC ATC GCC GGC CGG GTG GCC CAG ATC CGC ACC GAG ATC GAG AAC [ 8730]

#M.ap_K10 ... ... ... ... ... ... ... ... ... ... ... ... ... ... ... [ 8730]

#M._ap_S397 ... ... ... ... ... ... ... ... ... ... ... ... ... ... ... [ 8730]

#M._ap_JQ5 ... ... ... ... ... ... ... ... ... ... ... ... ... ... ... [ 8730]

#M._ap_JQ6 ... ... ... ... ... ... ... ... ... ... ... ... ... ... ... [ 8730]

#M._av_104 AGC GAC TCC GAC TAC GAC CGC GAG AAG CTG CAG GAG CGG CTG GCC [ 8775]

#M.ap_K10 ... ... ... ... ... ... ... ... ... ... ... ... ... ... ... [ 8775]

#M._ap_S397 ... ... ... ... ... ... ... ... ... ... ... ... ... ... ... [ 8775]

#M._ap_JQ5 ... ... ... ... ... ... ... ... ... ... ... ... ... ... ... [ 8775]

#M._ap_JQ6 ... ... ... ... ... ... ... ... ... ... ... ... ... ... ... [ 8775]

#M._av_104 AAG CTG GCC GGC GGC GTG GCG GTC ATC AAG GCC GGC GCC GCG ACC [ 8820]

#M.ap_K10 ... ... ... ... ... ... ... ..G ... ... ... ... ... ... ... [ 8820]

#M._ap_S397 ... ... ... ... ... ... ... ..G ... ... ... ... ... ... ... [ 8820]

#M._ap_JQ5 ... ... ... ... ... ... ... ..G ... ... ... ... ... ... ... [ 8820]

#M._ap_JQ6 ... ... ... ... ... ... ... ..G ... ... ... ... ... ... ... [ 8820]

#M._av_104 GAG GTC GAG CTC AAG GAG CGC AAG CAC CGC ATC GAG GAC GCG GTC [ 8865]

#M.ap_K10 ... ... ... ... ... ... ... ... ... ... ... ... ... ... ... [ 8865]

#M._ap_S397 ... ... ... ... ... ... ... ... ... ... ... ... ... ... ... [ 8865]

#M._ap_JQ5 ... ... ... ... ... ... ... ... ... ... ... ... ... ... ... [ 8865]

#M._ap_JQ6 ... ... ... ... ... ... ... ... ... ... ... ... ... ... ... [ 8865]

#M._av_104 CGC AAC GCC AAG GCG GCC GTG GAA GAG GGC ATC GTC GCC GGC GGT [ 8910]

#M.ap_K10 ... ... ... ... ... ... ... ..G ... ... ... ... ... ... ... [ 8910]

#M._ap_S397 ... ... ... ... ... ... ... ..G ... ... ... ... ... ... ... [ 8910]

#M._ap_JQ5 ... ... ... ... ... ... ... ..G ... ... ... ... ... ... ... [ 8910]

#M._ap_JQ6 ... ... ... ... ... ... ... ..G ... ... ... ... ... ... ... [ 8910]

#M._av_104 GGC GTG GCC CTG CTG CAC GCG ATC CCG GCG CTC GAC GAG CTG AAG [ 8955]

#M.ap_K10 ... ... ... ... ... ... ... ... ... ..T ..G ... ... ... ... [ 8955]

#M._ap_S397 ... ... ... ... ... ... ... ... ... ..C ..G ... ... ... ... [ 8955]

#M._ap_JQ5 ... ... ... ... ... ... ... ... ... ..C ..G ... ... ... ... [ 8955]

#M._ap_JQ6 ... ... ... ... ... ... ... ... ... ..C ..G ... ... ... ... [ 8955]

#M._av_104 CTC GAG GGC GAC GAG GCG ACC GGC GCC AAC ATC GTC CGG GTG GCC [ 9000]

#M.ap_K10 ... ... ... ..A ... ... ... ... ... ... ... ... ... ... ... [ 9000]

#M._ap_S397 ... ... ... ... ... ... ... ... ... ... ... ... ... ... ... [ 9000]

#M._ap_JQ5 ... ... ... ... ... ... ... ... ... ... ... ... ... ... ... [ 9000]

#M._ap_JQ6 ... ... ... ... ... ... ... ... ... ... ... ... ... ... ... [ 9000]

#M._av_104 CTC GAG GCT CCG CTG AAG CAG ATC GCC TTC AAC GGT GGC CTG GAG [ 9045]

#M.ap_K10 ... ... ... ... ... ... ... ... ... ... ... ... ... ... ... [ 9045]

#M._ap_S397 ... ... ... ... ... ... ... ... ... ... ... ... ... ... ... [ 9045]

#M._ap_JQ5 ... ... ... ... ... ... ... ... ... ... ... ... ... ... ... [ 9045]

#M._ap_JQ6 ... ... ... ... ... ... ... ... ... ... ... ... ... ... ... [ 9045]

#M._av_104 CCC GGC GTG GTG GCC GAG AAG GTC CGC AAC TCG CCC GCC GGT ACC [ 9090]

#M.ap_K10 ... ... ... ... ... ... ... ... ... ... ... ... ... ... ... [ 9090]

#M._ap_S397 ... ... ... ... ... ... ... ... ... ... ... ... ... ... ... [ 9090]

#M._ap_JQ5 ... ... ... ... ... ... ... ... ... ... ... ... ... ... ... [ 9090]

#M._ap_JQ6 ... ... ... ... ... ... ... ... ... ... ... ... ... ... ... [ 9090]

#M._av_104 GGC CTC AAC GCC GCC ACC GGT GAG TAC GAG GAC CTG CTC AAG GCC [ 9135]

#M.ap_K10 ... ... ... ... ... ... ... ... ... ... ... ... ... ... ... [ 9135]

#M._ap_S397 ... ... ... ... ... G.. ... ... ... ... ... ... ... ... ... [ 9135]

#M._ap_JQ5 ... ... ... ... ... G.. ... ... ... ... ... ... ... ... ... [ 9135]

#M._ap_JQ6 ... ... ... ... ... G.. ... ... ... ... ... ... ... ... ... [ 9135]

#M._av_104 GGC GTT GCC GAC CCG GTG AAG GTG ACC CGC TCG GCG CTG CAG AAC [ 9180]

#M.ap_K10 ... A.. ... ... ... ... ... ..C ... ... ... ... ... ... ... [ 9180]

#M._ap_S397 ... A.. ... ... ... ... ... ..C ... ... ... ... ... ... ... [ 9180]

#M._ap_JQ5 ... A.. ... ... ... ... ... ..C ... ... ... ... ... ... ... [ 9180]

#M._ap_JQ6 ... A.. ... ... ... ... ... ..C ... ... ... ... ... ... ... [ 9180]

#M._av_104 GCG GCG TCC ATC GCG GGG CTG TTC CTA ACC ACC GAG GCG GTC GTC [ 9225]

#M.ap_K10 ... ... ... ... ... ... ... ... ..G ... ... ... ... ... ... [ 9225]

#M._ap_S397 ... ... ... ... ... ... ... ... ..G ... ... ... ... ... ... [ 9225]

#M._ap_JQ5 ... ... ... ... ... ... ... ... ..G ... ... ... ... ... ... [ 9225]

#M._ap_JQ6 ... ... ... ... ... ... ... ... ..G ... ... ... ... ... ... [ 9225]

#M._av_104 GCC GAC AAG CCG GAG AAG GCG GCC GCT CCC GCG GGC GAC CCG ACC [ 9270]

#M.ap_K10 ... ... ... ... ... ... ... ... ... ... ... ... ... ... ... [ 9270]

#M._ap_S397 ... ... ... ... ... ... ... ... ... ... ... ... ... ... ... [ 9270]

#M._ap_JQ5 ... ... ... ... ... ... ... ... ... ... ... ... ... ... ... [ 9270]

#M._ap_JQ6 ... ... ... ... ... ... ... ... ... ... ... ... ... ... ... [ 9270]

#M._av_104 GGC GGC ATG GGC GGC ATG GAC TTC TGA ATG ACT GAC ACC ACG CTG [ 9315]

#M.ap_K10 ... ... ... ... ... ... ... ... ... ... ... ... ... ... ... [ 9315]

#M._ap_S397 ... ... ... ... ... ... ... ... ... ... ... ... ... ... ... [ 9315]

#M._ap_JQ5 ... ... ... ... ... ... ... ... ... ... ... ... ... ... ... [ 9315]

#M._ap_JQ6 ... ... ... ... ... ... ... ... ... ... ... ... ... ... ... [ 9315]

#M._av_104 CCA CCC GGC GGT GAC GCC GCC GAC CGC GTC GAA CCG GTC GAC ATC [ 9360]

#M.ap_K10 ... ... ... ... ... ... ... ... ... ... ... ... ... ... ... [ 9360]

#M._ap_S397 ... ... ... ... ... ... ... ... ... ... ... ... ... ... ... [ 9360]

#M._ap_JQ5 ... ... ... ... ... ... ... ... ... ... ... ... ... ... ... [ 9360]

#M._ap_JQ6 ... ... ... ... ... ... ... ... ... ... ... ... ... ... ... [ 9360]

#M._av_104 CAG CAG GAG ATG CAG CGC AGC TAC ATC GAT TAC GCG ATG AGC GTG [ 9405]

#M.ap_K10 ... ... ... ... ... ... ... ... ... ... ... ... ... ... ..A [ 9405]

#M._ap_S397 ... ... ... ... ... ... ... ... ... ... ... ... ... ... ..A [ 9405]

#M._ap_JQ5 ... ... ... ... ... ... ... ... ... ... ... ... ... ... ..A [ 9405]

#M._ap_JQ6 ... ... ... ... ... ... ... ... ... ... ... ... ... ... ..A [ 9405]

#M._av_104 ATC GTC GGC CGC GCG CTG CCC GAG GTG CGC GAC GGC CTC AAG CCG [ 9450]

#M.ap_K10 ... ... ... ... ... ... ... ... ... ... ... ... ... ... ... [ 9450]

#M._ap_S397 ... ... ... ... ... ... ... ... ... ... ... ... ... ... ... [ 9450]

#M._ap_JQ5 ... ... ... ... ... ... ... ... ... ... ... ... ... ... ... [ 9450]

#M._ap_JQ6 ... ... ... ... ... ... ... ... ... ... ... ... ... ... ... [ 9450]

#M._av_104 GTG CAC CGC CGG GTG CTC TAC GCC ATG TAC GAC TCG GGT TTC CGC [ 9495]

#M.ap_K10 ... ... ... ... ..A ..T ... ... ... ... ... ... ... ... ... [ 9495]

#M._ap_S397 ... ... ... ... ... ..T ... ... ... ... ... ... ... ... ... [ 9495]

#M._ap_JQ5 ... ... ... ... ... ..T ... ... ... ... ... ... ... ... ... [ 9495]

#M._ap_JQ6 ... ... ... ... ... ..T ... ... ... ... ... ... ... ... ... [ 9495]

#M._av_104 CCG GAC CGC AGC CAC GCC AAA TCG GCG CGG TCG GTC GCC GAA ACG [ 9540]

#M.ap_K10 ... ... ... ... ... ... ..G ... ... ... ... ... ... ..G ... [ 9540]

#M._ap_S397 ... ... ... ... ... ... ..G ... ... ... ... ... ... ..G ... [ 9540]

#M._ap_JQ5 ... ... ... ... ... ... ..G ... ... ... ... ... ... ..G ... [ 9540]

#M._ap_JQ6 ... ... ... ... ... ... ..G ... ... ... ... ... ... ..G ... [ 9540]

#M._av_104 ATG GGC AAC TAC CAC CCG CAC GGC GAC GCC TCG ATC TAC GAC ACC [ 9585]

#M.ap_K10 ... ... ... ... ... ... ... ... ... ... ... ... ... ... ... [ 9585]

#M._ap_S397 ... ... ... ... ... ... ... ... ... ... ... ... ... ... ... [ 9585]

#M._ap_JQ5 ... ... ... ... ... ... ... ... ... ... ... ... ... ... ... [ 9585]

#M._ap_JQ6 ... ... ... ... ... ... ... ... ... ... ... ... ... ... ... [ 9585]

#M._av_104 CTG GTG CGG ATG GCC CAG CCG TGG TCG CTG CGC TAT CCG TTG GTC [ 9630]

#M.ap_K10 ... ... ... ... ... ... ... ... ... ... ... ..C ... ... ... [ 9630]

#M._ap_S397 ... ... ... ... ... ... ... ... ... ... ... ..C ... ... ... [ 9630]

#M._ap_JQ5 ... ... ... ... ... ... ... ... ... ... ... ..C ... ... ... [ 9630]

#M._ap_JQ6 ... ... ... ... ... ... ... ... ... ... ... ..C ... ... ... [ 9630]

#M._av_104 GAC GGG CAG GGC AAT TTT GGT TCG CCG GGC AAC GAC CCG CCG GCC [ 9675]

#M.ap_K10 ... ... ... ... ..C ... ... ... ... ... ... ... ... ... ... [ 9675]

#M._ap_S397 ... ... ... ... ..C ... ... ... ... ... ... ... ... ... ... [ 9675]

#M._ap_JQ5 ... ... ... ... ..C ... ... ... ... ... ... ... ... ... ... [ 9675]

#M._ap_JQ6 ... ... ... ... ..C ... ... ... ... ... ... ... ... ... ... [ 9675]

#M._av_104 GCG ATG CGG TAC ACC GAG GCG CGG CTG ACC CCG CTG GCC ATG GAG [ 9720]

#M.ap_K10 ... ... ... ... ... ... ... ... ... ... ... ... ... ... ... [ 9720]

#M._ap_S397 ... ... ... ... ... ... ... ... ... ... ... ... ... ... ... [ 9720]

#M._ap_JQ5 ... ... ... ... ... ... ... ... ... ... ... ... ... ... ... [ 9720]

#M._ap_JQ6 ... ... ... ... ... ... ... ... ... ... ... ... ... ... ... [ 9720]

#M._av_104 ATG CTG CGC GAA ATC GAC GAG GAG ACA GTC GAT TTC ATT CCC AAC [ 9765]

#M.ap_K10 ... ... ... ... ... ... ... ... ... ... ... ... ... ... ... [ 9765]

#M._ap_S397 ... ... ... ... ... ... ... ... ... ... ... ... ... ... ... [ 9765]

#M._ap_JQ5 ... ... ... ... ... ... ... ... ... ... ... ... ... ... ... [ 9765]

#M._ap_JQ6 ... ... ... ... ... ... ... ... ... ... ... ... ... ... ... [ 9765]

#M._av_104 TAC GAC GGC CGG GTG CAA GAG CCG ACG GTG CTG CCC AGC CGG TTC [ 9810]

#M.ap_K10 ... ... ... ... ... ... ... ... ... ... ... ... ... ... ... [ 9810]

#M._ap_S397 ... ... ... ... ... ... ... ... ... ... ... ... ... ... ... [ 9810]

#M._ap_JQ5 ... ... ... ... ... ... ... ... ... ... ... ... ... ... ... [ 9810]

#M._ap_JQ6 ... ... ... ... ... ... ... ... ... ... ... ... ... ... ... [ 9810]

#M._av_104 CCC AAC CTG CTG GCC AAC GGG TCG GGG GGC ATC GCG GTC GGC ATG [ 9855]

#M.ap_K10 ... ... ... ... ... ... ... ... ... ... ... ... ... ... ... [ 9855]

#M._ap_S397 ... ... ... ... ... ... ... ... ... ... ... ... ... ... ... [ 9855]

#M._ap_JQ5 ... ... ... ... ... ... ... ... ... ... ... ... ... ... ... [ 9855]

#M._ap_JQ6 ... ... ... ... ... ... ... ... ... ... ... ... ... ... ... [ 9855]

#M._av_104 GCC ACC AAC ATC CCG CCG CAC AAC CTC GGC GAG CTC GCC GAG GCG [ 9900]

#M.ap_K10 ... ..G ... ... ... ... ... ... ... ... ... ... ... ... ... [ 9900]

#M._ap_S397 ... ..G ... ... ... ... ... ... ... ... ... ... ... ... ... [ 9900]

#M._ap_JQ5 ... ..G ... ... ... ... ... ... ... ... ... ... ... ... ... [ 9900]

#M._ap_JQ6 ... ..G ... ... ... ... ... ... ... ... ... ... ... ... ... [ 9900]

#M._av_104 GTG TTC TGG GCG CTG GAC AAT TAC GAG GCC GAC GAA GAG GCC ACC [ 9945]

#M.ap_K10 ... ... ... ... ... ... ... ... ... ... ... ... ... ... ... [ 9945]

#M._ap_S397 ... ... ... ... ... ... ... ... ... ... ... ... ... ... ... [ 9945]

#M._ap_JQ5 ... ... ... ... ... ... ... ... ... ... ... ... ... ... ... [ 9945]

#M._ap_JQ6 ... ... ... ... ... ... ... ... ... ... ... ... ... ... ... [ 9945]

#M._av_104 CTG GCC GCC GTG ATG GAA CGG GTG AAA GGA CCC GAC TTC CCG ACT [ 9990]

#M.ap_K10 ... ... ... ... ... ... ... ... ... ... ... ... ... ... ..C [ 9990]

#M._ap_S397 ... ... ... ... ... ... ... ... ... ... ... ... ... ... ..C [ 9990]

#M._ap_JQ5 ... ... ... ... ... ... ... ... ... ... ... ... ... ... ..C [ 9990]

#M._ap_JQ6 ... ... ... ... ... ... ... ... ... ... ... ... ... ... ..C [ 9990]

#M._av_104 TCG GGC CTG ATC GTC GGC ACG CAG GGC ATC GCC GAC GCC TAC AAG [10035]

#M.ap_K10 ..C ... ... ... ... ... ... ... ... ... ... ... ... ... ... [10035]

#M._ap_S397 ..C ... ... ... ... ... ... ... ... ... ... ... ... ... ... [10035]

#M._ap_JQ5 ..C ... ... ... ... ... ... ... ... ... ... ... ... ... ... [10035]

#M._ap_JQ6 ..C ... ... ... ... ... ... ... ... ... ... ... ... ... ... [10035]

#M._av_104 ACC GGC CGC GGC TCC ATC CGG ATG CGC GGA GTC GTT GAG GTG GAA [10080]

#M.ap_K10 ... ... ... ..T ... ... ... ... ... ... ... ... ... ... ... [10080]

#M._ap_S397 ... ... ... ..T ... ... ... ... ... ... ... ... ... ... ... [10080]

#M._ap_JQ5 ... ... ... ..T ... ... ... ... ... ... ... ... ... ... ... [10080]

#M._ap_JQ6 ... ... ... ..T ... ... ... ... ... ... ... ... ... ... ... [10080]

#M._av_104 GAG GAT TCG CGC GGT CGC ACC TCG CTG GTC ATC ACC GAG TTG CCG [10125]

#M.ap_K10 ... ... ... ... ..C ... ... ... ... ... ... ... ... ... ... [10125]

#M._ap_S397 ... ... ... ... ..C ... ... ... ... ... ... ... ... ... ... [10125]

#M._ap_JQ5 ... ... ... ... ..C ... ... ... ... ... ... ... ... ... ... [10125]

#M._ap_JQ6 ... ... ... ... ..C ... ... ... ... ... ... ... ... ... ... [10125]

#M._av_104 TAT CAG GTC AAC CAC GAC AAC TTC ATC ACC TCG ATC GCC GAG CAG [10170]

#M.ap_K10 ... ... ... ... ... ... ... ... ... ... ... ... ... ... ... [10170]

#M._ap_S397 ... ... ... ... ... ... ... ... ... ... ... ... ... ... ... [10170]

#M._ap_JQ5 ... ... ... ... ... ... ... ... ... ... ... ... ... ... ... [10170]

#M._ap_JQ6 ... ... ... ... ... ... ... ... ... ... ... ... ... ... ... [10170]

#M._av_104 GTG CGC GAC GGC AAG CTG GCC GGC ATC TCC AAC ATC GAG GAC CAG [10215]

#M.ap_K10 ... ... ... ... ... ... ... ... ... ... ..T ... ... ... ..A [10215]

#M._ap_S397 ... ... ... ... ... ... ... ... ... ... ..T ... ... ... ..A [10215]

#M._ap_JQ5 ... ... ... ... ... ... ... ... ... ... ..T ... ... ... ..A [10215]

#M._ap_JQ6 ... ... ... ... ... ... ... ... ... ... ..T ... ... ... ..A [10215]

#M._av_104 TCC AGC GAC CGG GTC GGG CTG CGC ATC GTC ATC GAG CTC AAG CGC [10260]

#M.ap_K10 ... ... ... ... ... ... ... ... ... ... ... ... ... ... ... [10260]

#M._ap_S397 ... ... ... ... ... ... ... ... ... ... ... ... ... ... ... [10260]

#M._ap_JQ5 ... ... ... ... ... ... ... ... ... ... ... ... ... ... ... [10260]

#M._ap_JQ6 ... ... ... ... ... ... ... ... ... ... ... ... ... ... ... [10260]

#M._av_104 GAC GCC GTC GCC AAG GTG GTG CTG AAC AAC CTC TAC AAG CAC ACC [10305]

#M.ap_K10 ... ... ... ... ... ... ... ... ... ... ... ... ... ... ... [10305]

#M._ap_S397 ... ... ... ... ... ... ... ... ... ... ... ... ... ... ... [10305]

#M._ap_JQ5 ... ... ... ... ... ... ... ... ... ... ... ... ... ... ... [10305]

#M._ap_JQ6 ... ... ... ... ... ... ... ... ... ... ... ... ... ... ... [10305]

#M._av_104 CAG CTG CAG ACC AGC TTC GGC GCC AAC ATG CTG GCC ATC GTC GAT [10350]

#M.ap_K10 ... ... ... ... ... ... ... ... ... ... ... ... ... ... ..C [10350]

#M._ap_S397 ... ... ... ... ... ... ... ... ... ... ... ... ... ... ..C [10350]

#M._ap_JQ5 ... ... ... ... ... ... ... ... ... ... ... ... ... ... ..C [10350]

#M._ap_JQ6 ... ... ... ... ... ... ... ... ... ... ... ... ... ... ..C [10350]

#M._av_104 GGG GTG CCG CGC ACC CTG CGG CTC GAC CAG CTG ATC CGC CAC TAC [10395]

#M.ap_K10 ... ... ... ... ... ... ... ... ... ... ... ... ... ... ... [10395]

#M._ap_S397 ... ... ... ... ... ... ... ... ... ... ... ... ... ... ... [10395]

#M._ap_JQ5 ... ... ... ... ... ... ... ... ... ... ... ... ... ... ... [10395]

#M._ap_JQ6 ... ... ... ... ... ... ... ... ... ... ... ... ... ... ... [10395]

#M._av_104 GTC GAC CAC CAA CTC GAC GTC ATC GTC CGG CGC ACC ACC TAC CGG [10440]

#M.ap_K10 ... ... ... ... ... ... ... ... ... ... ... ... ... ... ... [10440]

#M._ap_S397 ... ... ... ... ... ... ... ... ... ... ... ... ... ... ... [10440]

#M._ap_JQ5 ... ... ... ... ... ... ... ... ... ... ... ... ... ... ... [10440]

#M._ap_JQ6 ... ... ... ... ... ... ... ... ... ... ... ... ... ... ... [10440]

#M._av_104 TTG CGC AAG GCC AAC GAG CGG GCC CAC ATC CTG CGC GGT CTG GTC [10485]

#M.ap_K10 ... ... ... ... ... ... ... ... ... ... ... ... ... ... ... [10485]

#M._ap_S397 ... ... ... ... ... ... ... ... ... ... ... ... ... ... ... [10485]

#M._ap_JQ5 ... ... ... ... ... ... ... ... ... ... ... ... ... ... ... [10485]

#M._ap_JQ6 ... ... ... ... ... ... ... ... ... ... ... ... ... ... ... [10485]

#M._av_104 AAG GCG CTC GAT GCG CTC GAC GAG GTC ATC GCC CTG ATC CGG GCG [10530]

#M.ap_K10 ... ... ... ... ... ... ... ... ... ... ... ... ... ... ... [10530]

#M._ap_S397 ... ... ... ... ... ... ... ... ... ... ... ... ... ... ... [10530]

#M._ap_JQ5 ... ... ... ... ... ... ... ... ... ... ... ... ... ... ... [10530]

#M._ap_JQ6 ... ... ... ... ... ... ... ... ... ... ... ... ... ... ... [10530]

#M._av_104 TCG GAA ACC GTC GAC ATC GCG CGG CAG GGC TTG ATC GAG CTG CTC [10575]

#M.ap_K10 ... ... ... ... ... ... ... ... ... ... ... ... ... ... ... [10575]

#M._ap_S397 ... ... ... ... ... ... ... ... ... ... ... ... ... ... ... [10575]

#M._ap_JQ5 ... ... ... ... ... ... ... ... ... ... ... ... ... ... ... [10575]

#M._ap_JQ6 ... ... ... ... ... ... ... ... ... ... ... ... ... ... ... [10575]

#M._av_104 GAC ATC GAC GAG ATC CAG GCC CAG GCG ATC CTG GAC ATG CAG CTG [10620]

#M.ap_K10 ... ... ... ... ... ... ..G ... ... ... ... ... ... ... ... [10620]

#M._ap_S397 ... ... ... ... ... ... ..G ... ... ... ... ... ... ... ... [10620]

#M._ap_JQ5 ... ... ... ... ... ... ..G ... ... ... ... ... ... ... ... [10620]

#M._ap_JQ6 ... ... ... ... ... ... ..G ... ... ... ... ... ... ... ... [10620]

#M._av_104 CGC CGG CTG GCC GCC CTG GAG CGG CAG CGC ATC ATC GAC GAC CTG [10665]

#M.ap_K10 ... ... ... ... ..G ... ... ... ... ... ... ... ... ... ... [10665]

#M._ap_S397 ... ... ... ... ..G ... ... ... ... ... ... ... ... ... ... [10665]

#M._ap_JQ5 ... ... ... ... ..G ... ... ... ... ... ... ... ... ... ... [10665]

#M._ap_JQ6 ... ... ... ... ..G ... ... ... ... ... ... ... ... ... ... [10665]

#M._av_104 GCC AAG ATC GAG GCC GAG ATC GCC GAC CTG GAG GAC ATC CTG GCC [10710]

#M.ap_K10 ... ... ... ... ... ... ... ... ... ... ... ... ... ... ... [10710]

#M._ap_S397 ... ... ... ... ... ... ... ... ... ... ... ... ... ... ... [10710]

#M._ap_JQ5 ... ... ... ... ... ... ... ... ... ... ... ... ... ... ... [10710]

#M._ap_JQ6 ... ... ... ... ... ... ... ... ... ... ... ... ... ... ... [10710]

#M._av_104 AAG CCG GAA CGG CAA CGC GGC ATT GTG CGC GAC GAG CTC GCC GAG [10755]

#M.ap_K10 ... ... ... ... ..G ... ... ..C ... ... ... ... ... ... ... [10755]

#M._ap_S397 ... ... ... ... ..G ... ... ..C ... ... ... ... ... ... ... [10755]

#M._ap_JQ5 ... ... ... ... ..G ... ... ..C ... ... ... ... ... ... ... [10755]

#M._ap_JQ6 ... ... ... ... ..G ... ... ..C ... ... ... ... ... ... ... [10755]

#M._av_104 ATC GTC GAA AAG CAC GGC GAC GCG CGG CGC ACC CGG ATC GTG GCC [10800]

#M.ap_K10 ... ... ... ... ... ... ... ... ... ... ... ... ... ... ... [10800]

#M._ap_S397 ... ... ... ... ... ... ... ... ... ... ... ... ... ... ... [10800]

#M._ap_JQ5 ... ... ... ... ... ... ... ... ... ... ... ... ... ... ... [10800]

#M._ap_JQ6 ... ... ... ... ... ... ... ... ... ... ... ... ... ... ... [10800]

#M._av_104 GCC GAC GGC GAC GTC AGC GAC GAG GAT CTG ATC GCC CGC GAG GAC [10845]

#M.ap_K10 ... ... ... ... ..T ... ... ... ... ... ... ..T ... ... ... [10845]

#M._ap_S397 ... ... ... ... ..T ... ... ... ... ... ... ..T ... ... ... [10845]

#M._ap_JQ5 ... ... ... ... ..T ... ... ... ... ... ... ..T ... ... ... [10845]

#M._ap_JQ6 ... ... ... ... ..T ... ... ... ... ... ... ..T ... ... ... [10845]

#M._av_104 GTC GTC GTC ACC ATC ACC GAG ACC GGC TAC GCC AAG CGC ACC AAG [10890]

#M.ap_K10 ... ... ... ... ... ... ... ... ... ... ... ... ... ... ... [10890]

#M._ap_S397 ... ... ... ... ... ... ... ... ... ... ... ... ... ... ... [10890]

#M._ap_JQ5 ... ... ... ... ... ... ... ... ... ... ... ... ... ... ... [10890]

#M._ap_JQ6 ... ... ... ... ... ... ... ... ... ... ... ... ... ... ... [10890]

#M._av_104 ACC GAC CTG TAC CGC AGC CAG AAG CGG GGC GGC AAG GGC GTG CAG [10935]

#M.ap_K10 ... ... ... ... ... ... ... ... ..C ... ... ... ... ... ... [10935]

#M._ap_S397 ... ... ... ... ... ... ... ... ..C ... ... ... ... ... ... [10935]

#M._ap_JQ5 ... ... ... ... ... ... ... ... ..C ... ... ... ... ... ... [10935]

#M._ap_JQ6 ... ... ... ... ... ... ... ... ..C ... ... ... ... ... ... [10935]

#M._av_104 GGC GCC GGC CTC AAA CAG GAC GAC ATC GTG CGG CAC TTC TTC GTG [10980]

#M.ap_K10 ... ... ... ... ..C ... ... ... ... ... ... ... ... ... ... [10980]

#M._ap_S397 ... ... ... ... ... ... ... ... ... ... ... ... ... ... ... [10980]

#M._ap_JQ5 ... ... ... ... ... ... ... ... ... ... ... ... ... ... ... [10980]

#M._ap_JQ6 ... ... ... ... ... ... ... ... ... ... ... ... ... ... ... [10980]

#M._av_104 TGC TCG ACG CAC GAC TGG ATC CTG TTC TTC ACC ACC CAG GGC CGG [11025]

#M.ap_K10 ... ... ... ... ... ... ... ... ... ... ... ... ... ... ... [11025]

#M._ap_S397 ... ... ... ... ... ... ... ... ... ... ... ... ... ... ... [11025]

#M._ap_JQ5 ... ... ... ... ... ... ... ... ... ... ... ... ... ... ... [11025]

#M._ap_JQ6 ... ... ... ... ... ... ... ... ... ... ... ... ... ... ... [11025]

#M._av_104 GTC TAC CGC GCC AAG GCC TAC GAA CTG CCC GAG GCG TCT CGC ACC [11070]

#M.ap_K10 ... ... ... ... ... ... ... ... ... ... ... ... ..C ... ... [11070]

#M._ap_S397 ... ... ... ... ... ... ... ... ... ... ... ... ..C ... ... [11070]

#M._ap_JQ5 ... ... ... ... ... ... ... ... ... ... ... ... ..C ... ... [11070]

#M._ap_JQ6 ... ... ... ... ... ... ... ... ... ... ... ... ..C ... ... [11070]

#M._av_104 GCC CGC GGT CAG CAC GTG GCC AAC CTG CTG GCG TTC CAG CCC GAG [11115]

#M.ap_K10 ... ... ... ... ... ... ... ... ... ... ... ... ... ... ... [11115]

#M._ap_S397 ... ... ... ... ... ... ... ... ... ... ... ... ... ... ... [11115]

#M._ap_JQ5 ... ... ... ... ... ... ... ... ... ... ... ... ... ... ... [11115]

#M._ap_JQ6 ... ... ... ... ... ... ... ... ... ... ... ... ... ... ... [11115]

#M._av_104 GAG CGG ATC GCC CAG GTG ATC CAG ATC CGC AGC TAT GAG GAC GCC [11160]

#M.ap_K10 ... ... ... ..T ... ... ... ... ... ..G ... ... ... ... ..T [11160]

#M._ap_S397 ... A.. ... ..T ... ... ... ... ... ..G ... ... ... ... ..T [11160]

#M._ap_JQ5 ... A.. ... ..T ... ... ... ... ... ..G ... ... ... ... ..T [11160]

#M._ap_JQ6 ... A.. ... ..T ... ... ... ... ... ..G ... ... ... ... ..T [11160]

#M._av_104 CCC TAC CTG GTG CTG GCC ACC CGC AAC GGC CTG GTG AAG AAG ACC [11205]

#M.ap_K10 ... ... ... ... ... ... ... ... ... ... ... ... ... ... ... [11205]

#M._ap_S397 ... ... ... ... ... ... ... ... ... ... ... ... ... ... ... [11205]

#M._ap_JQ5 ... ... ... ... ... ... ... ... ... ... ... ... ... ... ... [11205]

#M._ap_JQ6 ... ... ... ... ... ... ... ... ... ... ... ... ... ... ... [11205]

#M._av_104 AAG CTG ACC GAC TTC GAC TCG AAC CGT TCG GGC GGC ATC GTG GCG [11250]

#M.ap_K10 ... ... ... ... ... ... ... ... ..C ... ... ... ... ... ... [11250]

#M._ap_S397 ... ... ... ... ... ... ... ... ..C ... ... ... ... ... ... [11250]

#M._ap_JQ5 ... ... ... ... ... ... ... ... ..C ... ... ... ... ... ... [11250]

#M._ap_JQ6 ... ... ... ... ... ... ... ... ..C ... ... ... ... ... ... [11250]

#M._av_104 ATC AAC CTG CGC GAC AAC GAC GAA CTC GTG GGC GCG GTG TTG TGC [11295]

#M.ap_K10 ... ... ... ... ... ... ... ... ... ... ... ... ... ... ... [11295]

#M._ap_S397 ... ... ... ... ... ... ... ... ... ... ..T ... ... ... ... [11295]

#M._ap_JQ5 ... ... ... ... ... ... ... ... ... ... ..T ... ... ... ... [11295]

#M._ap_JQ6 ... ... ... ... ... ... ... ... ... ... ..T ... ... ... ... [11295]

#M._av_104 TCG GCC GAG GAC GAT CTG CTG CTG GTG TCG GCC AAC GGC CAG TCC [11340]

#M.ap_K10 ... ... ... ... ... ... ... ... ... ... ... ... ... ... ... [11340]

#M._ap_S397 ... ... ... ... ... ... ... ... ... ... ... ... ... ... ... [11340]

#M._ap_JQ5 ... ... ... ... ... ... ... ... ... ... ... ... ... ... ... [11340]

#M._ap_JQ6 ... ... ... ... ... ... ... ... ... ... ... ... ... ... ... [11340]

#M._av_104 ATC CGG TTC TCG GCG ACC GAC GAG GCG CTG CGC CCG ATG GGC CGC [11385]

#M.ap_K10 ... ... ... ... ... ... ... ... ... ... ... ... ... ... ... [11385]

#M._ap_S397 ... ... ... ... ... ... ... ... ... ... ... ... ... ... ... [11385]

#M._ap_JQ5 ... ... ... ... ... ... ... ... ... ... ... ... ... ... ... [11385]

#M._ap_JQ6 ... ... ... ... ... ... ... ... ... ... ... ... ... ... ... [11385]

#M._av_104 GCC ACC TCC GGT GTG CAG GGC ATG CGC TTC AAC GCC GAC GAC TAC [11430]

#M.ap_K10 ... ... ... ... ... ... ... ... ... ... ... ... ... ... ... [11430]

#M._ap_S397 ... ... ... ... ... ... ... ... ... ... ... ... ... ... ... [11430]

#M._ap_JQ5 ... ... ... ... ... ... ... ... ... ... ... ... ... ... ... [11430]

#M._ap_JQ6 ... ... ... ... ... ... ... ... ... ... ... ... ... ... ... [11430]

#M._av_104 CTG CTG TCG CTC AAC GTG GTC CGC GAG GGC ACC TAC CTG CTG GTG [11475]

#M.ap_K10 ... ... ... ... ... ... ... ... ... ... ... ... ... ... ... [11475]

#M._ap_S397 ... ... ... ... ... ... ... ... ... ... ... ... ... ... ... [11475]

#M._ap_JQ5 ... ... ... ... ... ... ... ... ... ... ... ... ... ... ... [11475]

#M._ap_JQ6 ... ... ... ... ... ... ... ... ... ... ... ... ... ... ... [11475]

#M._av_104 GCG ACG TCC GGC GGG TAC GCC AAG CGC ACC GCG ATC GAG GAG TAT [11520]

#M.ap_K10 ... ... ... ... ... ... ... ... ... ... ... ... ... ... ... [11520]

#M._ap_S397 ... ... ... ... ... ... ... ... ... ... ... ... ... ... ... [11520]

#M._ap_JQ5 ... ... ... ... ... ... ... ... ... ... ... ... ... ... ... [11520]

#M._ap_JQ6 ... ... ... ... ... ... ... ... ... ... ... ... ... ... ... [11520]

#M._av_104 CCG GTG CAG GGC CGC GGC GGC AAG GGC GTG CTG ACC GTG ATG TAT [11565]

#M.ap_K10 ... ... ... ... ... ... ... ... ... ... ... ... ... ... ... [11565]

#M._ap_S397 ... ... ... ... ... ... ... ... ... ... ... ... ... ... ... [11565]

#M._ap_JQ5 ... ... ... ... ... ... ... ... ... ... ... ... ... ... ... [11565]

#M._ap_JQ6 ... ... ... ... ... ... ... ... ... ... ... ... ... ... ... [11565]

#M._av_104 GAC CGC CGC CGT GGC AGG CTG GTG GGT GCG CTG ATT GTG GAC GAG [11610]

#M.ap_K10 ... ... ... ... ... ... ... ... ... ... ... ... ... ... ... [11610]

#M._ap_S397 ... ... ... ... ... ... ... ... ... ... ... ... ... ... ... [11610]

#M._ap_JQ5 ... ... ... ... ... ... ... ... ... ... ... ... ... ... ... [11610]

#M._ap_JQ6 ... ... ... ... ... ... ... ... ... ... ... ... ... ... ... [11610]

#M._av_104 GAC AGC GAG CTG TAC GCG ATC ACC TCC GGC GGC GGT GTC ATC CGC [11655]

#M.ap_K10 ... ... ... ... ... ... ... ... ... ... ... ... ... ... ... [11655]

#M._ap_S397 ... ... ... ... ... ... ... ... ... ... ... ... ... ... ... [11655]

#M._ap_JQ5 ... ... ... ... ... ... ... ... ... ... ... ... ... ... ... [11655]

#M._ap_JQ6 ... ... ... ... ... ... ... ... ... ... ... ... ... ... ... [11655]

#M._av_104 ACC GCG GCG GGC CAG GTC CGT AAG GCG GGA CGG CAG ACC AAG GGC [11700]

#M.ap_K10 ... ... ... ... ... ... ... ... ... ... ... ... ... ... ... [11700]

#M._ap_S397 ... ... ... ... ... ... ... ... ... ... ... ... ... ... ... [11700]

#M._ap_JQ5 ... ... ... ... ... ... ... ... ... ... ... ... ... ... ... [11700]

#M._ap_JQ6 ... ... ... ... ... ... ... ... ... ... ... ... ... ... ... [11700]

#M._av_104 GTC CGG CTG ATG AAT CTG GGT GAG GGC GAC ACG CTG CTG GCC ATC [11745]

#M.ap_K10 ... ... ... ... ... ... ... ... ... ... ... ... ... ... ... [11745]

#M._ap_S397 ... ... ... ... ... ... ... ... ... ... ... ... ... ... ... [11745]

#M._ap_JQ5 ... ... ... ... ... ... ... ... ... ... ... ... ... ... ... [11745]

#M._ap_JQ6 ... ... ... ... ... ... ... ... ... ... ... ... ... ... ... [11745]

#M._av_104 GCC CGC AAC GCC GAG GAA GCC GCG GAC GAG GCC GTC GAG GAG AGC [11790]

#M.ap_K10 ..T ... ... ... ... ... ... ... ... ... ... ... ..C ... ... [11790]

#M._ap_S397 ..T ... ... ... ... ... ... ... ... ... ... ... ..C ... ... [11790]

#M._ap_JQ5 ..T ... ... ... ... ... ... ... ... ... ... ... ..C ... ... [11790]

#M._ap_JQ6 ..T ... ... ... ... ... ... ... ... ... ... ... ..C ... ... [11790]

#M._av_104 GAC GGT GCC GCG GGG TCG GAC GGC TAG GTG GCT GCC CAG AAG AAG [11835]

#M.ap_K10 ... ... ... ... ... ... ... ... ... ... ... ... ... ... ... [11835]

#M._ap_S397 ... ... ... ... ... ... ... ... ... ... ... ... ... ... ... [11835]

#M._ap_JQ5 ... ... ... ... ... ... ... ... ... ... ... ... ... ... ... [11835]

#M._ap_JQ6 ... ... ... ... ... ... ... ... ... ... ... ... ... ... ... [11835]

#M._av_104 AAG GCG CAA GAC GAA TAC GGT GCT TCA GCG ATC ACC GTC CTG GAA [11880]

#M.ap_K10 ... ... ... ... ... ... ... ... ... ... ... ... ... ... ... [11880]

#M._ap_S397 ... ... ... ... ... ... ... ... ... ... ... ... ... ... ... [11880]

#M._ap_JQ5 ... ... ... ... ... ... ... ... ... ... ... ... ... ... ... [11880]

#M._ap_JQ6 ... ... ... ... ... ... ... ... ... ... ... ... ... ... ... [11880]

#M._av_104 GGG CTG GAG GCG GTC CGC AAA CGC CCC GGT ATG TAC ATC GGC TCC [11925]

#M.ap_K10 ..A ... ... ... ... ... ... ... ... ..C ... ... ... ... ..T [11925]

#M._ap_S397 ..A ... ... ... ... ... ... ... ... ..C ... ... ... ... ... [11925]

#M._ap_JQ5 ..A ... ... ... ... ... ... ... ... ..C ... ... ... ... ... [11925]

#M._ap_JQ6 ..A ... ... ... ... ... ... ... ... ..C ... ... ... ... ... [11925]

#M._av_104 ACC GGC GAG CGA GGT CTG CAC CAC CTC ATC TGG GAG GTG GTC GAC [11970]

#M.ap_K10 ... ... ... ... ... ... ... ... ... ... ... ... ... ... ... [11970]

#M._ap_S397 ... ... ... ... ... ... ... ... ... ... ... ... ... ... ... [11970]

#M._ap_JQ5 ... ... ... ... ... ... ... ... ... ... ... ... ... ... ... [11970]

#M._ap_JQ6 ... ... ... ... ... ... ... ... ... ... ... ... ... ... ... [11970]

#M._av_104 AAC TCG GTC GAC GAA GCG ATG GCC GGC TAC GCC GAC CGG GTC GAC [12015]

#M.ap_K10 ... ... ... ... ... ... ... ... ... ... ... ... ... ... ... [12015]

#M._ap_S397 ... ... ... ... ... ... ... ... ... ... ... ... ... ... ... [12015]

#M._ap_JQ5 ... ... ... ... ... ... ... ... ... ... ... ... ... ... ... [12015]

#M._ap_JQ6 ... ... ... ... ... ... ... ... ... ... ... ... ... ... ... [12015]

#M._av_104 GTG CGG ATC CTG GAC GAC GGC AGC GTC GAG GTC GCC GAC AAC GGC [12060]

#M.ap_K10 ... ... ... ... ... ... ... ... ..T ... ... ... ... ... ... [12060]

#M._ap_S397 ... ... ... ... ... ... ... ... ..T ... ... ... ... ... ... [12060]

#M._ap_JQ5 ... ... ... ... ... ... ... ... ..T ... ... ... ... ... ... [12060]

#M._ap_JQ6 ... ... ... ... ... ... ... ... ..T ... ... ... ... ... ... [12060]

#M._av_104 CGC GGC ATC CCC GTC GCG ATG CAC GCG ACC GGC GCC CCC ACC GTC [12105]

#M.ap_K10 ... ... ... ... ... ... ... ... ... ... ... ... ... ... ... [12105]

#M._ap_S397 ... ... ... ... ... ... ..T ... ... ... ... ... ... ... ... [12105]

#M._ap_JQ5 ... ... ... ... ... ... ..T ... ... ... ... ... ... ... ... [12105]

#M._ap_JQ6 ... ... ... ... ... ... ..T ... ... ... ... ... ... ... ... [12105]

#M._av_104 GAC GTG GTG ATG ACG CAG CTG CAC GCC GGC GGC AAG TTC GGC GGC [12150]

#M.ap_K10 ... ... ... ... ... ... ... ... ... ... ... ... ... ... ... [12150]

#M._ap_S397 ... ... ... ... ... ... ... ... ... ... ... ... ... ... ... [12150]

#M._ap_JQ5 ... ... ... ... ... ... ... ... ... ... ... ... ... ... ... [12150]

#M._ap_JQ6 ... ... ... ... ... ... ... ... ... ... ... ... ... ... ... [12150]

#M._av_104 GAA AAC AGC GGC TAC AAC GTC AGC GGC GGT CTG CAC GGC GTC GGC [12195]

#M.ap_K10 ... ... ... ... ... ... ... ... ... ... ... ... ... ... ... [12195]

#M._ap_S397 ... ... ... ... ... ... ... ... ... ... ... ... ... ... ... [12195]

#M._ap_JQ5 ... ... ... ... ... ... ... ... ... ... ... ... ... ... ... [12195]

#M._ap_JQ6 ... ... ... ... ... ... ... ... ... ... ... ... ... ... ... [12195]

#M._av_104 GTC TCG GTG GTC AAC GCA CTG TCC ACC CGG CTC GAG GTC AAC ATC [12240]

#M.ap_K10 ... ... ... ... ... ... ... ... ... ... ... ... ... ... ... [12240]

#M._ap_S397 ... ... ... ... ... ... ... ... ... ... ... ... ... ... ... [12240]

#M._ap_JQ5 ... ... ... ... ... ... ... ... ... ... ... ... ... ... ... [12240]

#M._ap_JQ6 ... ... ... ... ... ... ... ... ... ... ... ... ... ... ... [12240]

#M._av_104 GCC CGC GAT GGC TAC GAG TGG TCG CAG TAC TAC GAC CAC GCC GTG [12285]

#M.ap_K10 ... ... ..C ... ... ... ... ... ... ... ... ... ... ... ... [12285]

#M._ap_S397 ... ... ..C ... ... ... ... ... ... ... ... ... ... ... ... [12285]

#M._ap_JQ5 ... ... ..C ... ... ... ... ... ... ... ... ... ... ... ... [12285]

#M._ap_JQ6 ... ... ..C ... ... ... ... ... ... ... ... ... ... ... ... [12285]

#M._av_104 CCC GGC ACC CTC AAG CAG GGC GAG GCC ACC AAG CGC ACC GGC ACC [12330]

#M.ap_K10 ... ... ... ... ..A ... ... ... ... ... ... ... ... ... ... [12330]

#M._ap_S397 ... ... ... ... ..A ... ... ... .T. ... ... ... ... ... ... [12330]

#M._ap_JQ5 ... ... ... ... ..A ... ... ..A .T. ... ... ... ... ... ... [12330]

#M._ap_JQ6 ... ... ... ... ..A ... ... ..A .T. ... ... ... ... ... ... [12330]

#M._av_104 ACC ATC CGG TTC TGG GCC GAC CCC GAC ATC TTC GAG ACC ACC GAG [12375]

#M.ap_K10 ... ... ... ... ... ... ... ... ... ... ... ... ... ... ... [12375]

#M._ap_S397 ... ... ... ... ... ... ... ... ... ... ... ... ... ... ... [12375]

#M._ap_JQ5 ... ... ... ... ... ... ... ... ... ... ... ... ... ... ... [12375]

#M._ap_JQ6 ... ... ... ... ... ... ... ... ... ... ... ... ... ... ... [12375]

#M._av_104 TAC GAC TTC GAA ACG GTG GCC CGG CGG CTG CAG GAA ATG GCG TTC [12420]

#M.ap_K10 ... ... ... ... ... ... ... ..A ... ... ... ... ... ... ... [12420]

#M._ap_S397 ... ... ... ... ... ... ... ..A ... ... ... ... ... ... ... [12420]

#M._ap_JQ5 ... ... ... ... ... ... ... ..A ... ... ... ... ... ... ... [12420]

#M._ap_JQ6 ... ... ... ... ... ... ... ..A ... ... ... ... ... ... ... [12420]

#M._av_104 CTC AAC AAG GGC CTG ACC ATC AAC CTC ACC GAC GAG CGG GTG ACC [12465]

#M.ap_K10 ... ... ... ... ... ... ... ... ... ... ... ... ... ... ... [12465]

#M._ap_S397 ... ... ... ... ... ... ... ... ... ... ... ... ... ... ... [12465]

#M._ap_JQ5 ... ... ... ... ... ... ... ... ... ... ... ... ... ... ... [12465]

#M._ap_JQ6 ... ... ... ... ... ... ... ... ... ... ... ... ... ... ... [12465]

#M._av_104 AAC GAA GAG GTC GTC GAC GAG GTG GTC AGC GAC ACC GCC GAC GCA [12510]

#M.ap_K10 ... ... ... ... ... ... ... ... ... ... ... ... ... ... ... [12510]

#M._ap_S397 ... ... ... ... ... ... ... ... ... ... ... ... ... ... ... [12510]

#M._ap_JQ5 ... ... ... ... ... ... ... ... ... ... ... ... ... ... ... [12510]

#M._ap_JQ6 ... ... ... ... ... ... ... ... ... ... ... ... ... ... ... [12510]

#M._av_104 CCC AAG TCG GCG CAG GAG AAG GCG GCG GAA TCG GCT GCG CCG CAT [12555]

#M.ap_K10 ... ... ... ... ... ... ... ... ... ... ... ... ... ... ... [12555]

#M._ap_S397 ... ... ... ... ... ... ... ... ... ... ... ... ... ... ... [12555]

#M._ap_JQ5 ... ... ... ... ... ... ... ... ... ... ... ... ... ... ... [12555]

#M._ap_JQ6 ... ... ... ... ... ... ... ... ... ... ... ... ... ... ... [12555]

#M._av_104 AAG GTC AAG CAC CGC ACC TTC CAC TAC CCC GGC GGC CTG GTC GAC [12600]

#M.ap_K10 ... ... ... ... ... ... ... ... ... ... ... ... ... ... ... [12600]

#M._ap_S397 ... ... ... ... ... ... ... ... ... ... ... ... ... ... ... [12600]

#M._ap_JQ5 ... ... ... ... ... ... ... ... ... ... ... ... ... ... ... [12600]

#M._ap_JQ6 ... ... ... ... ... ... ... ... ... ... ... ... ... ... ... [12600]

#M._av_104 TTC GTC AAA CAC ATC AAT CGC ACC AAA AAC CCC ATC CAC CAG AGC [12645]

#M.ap_K10 ... ... ... ... ... ... ... ... ... ... ... ... ... ... ... [12645]

#M._ap_S397 ... ... ... ... ... ... ... ... ... ... ... ... ... ... ... [12645]

#M._ap_JQ5 ... ... ... ... ... ... ... ... ... ... ... ... ... ... ... [12645]

#M._ap_JQ6 ... ... ... ... ... ... ... ... ... ... ... ... ... ... ... [12645]

#M._av_104 ATC ATC GAT TTC GGT GGG AAG GGC CCC GGC CAC GAG GTC GAG ATC [12690]

#M.ap_K10 ... ... ... ... ... ... ... ... ... ... ... ... ... ... ... [12690]

#M._ap_S397 ... ... ... ... ... ... ... ... ... ... ... ... ... ... ... [12690]

#M._ap_JQ5 ... ... ... ... ... ... ... ... ... ... ... ... ... ... ... [12690]

#M._ap_JQ6 ... ... ... ... ... ... ... ... ... ... ... ... ... ... ... [12690]

#M._av_104 GCG ATG CAG TGG AAC GGC GGC TAT TCC GAA TCG GTG CAC ACG TTC [12735]

#M.ap_K10 ... ... ... ... ... ... ... ... ... ... ... ... ... ... ... [12735]

#M._ap_S397 ... ... ... ... ... ... ... ... ... ... ... ... ... ... ... [12735]

#M._ap_JQ5 ... ... ... ... ... ... ... ... ... ... ... ... ... ... ... [12735]

#M._ap_JQ6 ... ... ... ... ... ... ... ... ... ... ... ... ... ... ... [12735]

#M._av_104 GCC AAC ACC ATC AAC ACG CAC GAG GGC GGC ACC CAC GAG GAG GGC [12780]

#M.ap_K10 ... ... ... ... ... ... ... ... ... ... ... ... ... ... ... [12780]

#M._ap_S397 ... ... ... ... ... ... ... ... ... ... ... ... ... ... ... [12780]

#M._ap_JQ5 ... ... ... ... ... ... ... ... ... ... ... ... ... ... ... [12780]

#M._ap_JQ6 ... ... ... ... ... ... ... ... ... ... ... ... ... ... ... [12780]

#M._av_104 TTC CGC AGC GCG TTG ACC TCC GTG GTC AAC AAG TAC GCC AAG GAC [12825]

#M.ap_K10 ... ... ... ... ... ... ... ... ... ... ... ... ... ... ... [12825]

#M._ap_S397 ... ... ... ... ... ... ... ... ... ... ... ... ... ... ... [12825]

#M._ap_JQ5 ... ... ... ... ... ... ... ... ... ... ... ... ... ... ... [12825]

#M._ap_JQ6 ... ... ... ... ... ... ... ... ... ... ... ... ... ... ... [12825]

#M._av_104 AAG AAG CTG CTC AAG GAC AAG GAC CCC AAC CTC ACC GGC GAC GAC [12870]

#M.ap_K10 ... ... ... ... ... ... ... ... ... ... ... ... ... ... ... [12870]

#M._ap_S397 ... ... ... ... ... ... ... ... ... ... ... ... ... ... ... [12870]

#M._ap_JQ5 ... ... ... ... ... ... ... ... ... ... ... ... ... ... ... [12870]

#M._ap_JQ6 ... ... ... ... ... ... ... ... ... ... ... ... ... ... ... [12870]

#M._av_104 ATC CGC GAG GGT TTG GCC GCG GTG ATC TCG GTC AAG GTG AGC GAA [12915]

#M.ap_K10 ... ... ... ... ... ... ... ... ... ... ... ... ... ... ... [12915]

#M._ap_S397 ... ... ... ... ... ... ... ... ... ... ... ... ... ... ... [12915]

#M._ap_JQ5 ... ... ... ... ... ... ... ... ... ... ... ... ... ... ... [12915]

#M._ap_JQ6 ... ... ... ... ... ... ... ... ... ... ... ... ... ... ... [12915]

#M._av_104 CCG CAG TTC GAG GGC CAG ACC AAG ACC AAA CTG GGC AAC ACC GAG [12960]

#M.ap_K10 ... ... ... ... ... ... ... ... ... ... ... ... ... ... ... [12960]

#M._ap_S397 ... ... ... ... ... ... ... ... ... ... ... ... ... ... ... [12960]

#M._ap_JQ5 ... ... ... ... ... ... ... ... ... ... ... ... ... ... ... [12960]

#M._ap_JQ6 ... ... ... ... ... ... ... ... ... ... ... ... ... ... ... [12960]

#M._av_104 GTG AAG TCG TTC GTG CAG AAG GTG TGC AAC GAA CAG CTC ACC CAC [13005]

#M.ap_K10 ... ... ... ... ... ... ... ... ... ... ... ... ... ... ... [13005]

#M._ap_S397 ... ... ... ... ... ... ... ... ... ... ... ... ... ... ... [13005]

#M._ap_JQ5 ... ... ... ... ... ... ... ... ... ... ... ... ... ... ... [13005]

#M._ap_JQ6 ... ... ... ... ... ... ... ... ... ... ... ... ... ... ... [13005]

#M._av_104 TGG TTC GAA GCC AAC CCC GCA GAC GCC AAA GTC ATT GTC AAC AAG [13050]

#M.ap_K10 ... ... ... ... ... ... ... ... ... ... ... ... ... ... ... [13050]

#M._ap_S397 ... ... ... ... ... ... ... ... ... ... ... ... ... ... ... [13050]

#M._ap_JQ5 ... ... ... ... ... ... ... ... ... ... ... ... ... ... ... [13050]

#M._ap_JQ6 ... ... ... ... ... ... ... ... ... ... ... ... ... ... ... [13050]

#M._av_104 GCG GTT TCG TCA GCG CAG GCG CGC ATT GCC GCC CGC AAG GCG CGA [13095]

#M.ap_K10 ... ... ... ... ... ... ... ... ... ... ..G ... ... ... ... [13095]

#M._ap_S397 ... ... ... ... ... ... ... ... ... ... ..G ... ... ... ... [13095]

#M._ap_JQ5 ... ... ... ... ... ... ... ... ... ... ..G ... ... ... ... [13095]

#M._ap_JQ6 ... ... ... ... ... ... ... ... ... ... ..G ... ... ... ... [13095]

#M._av_104 GAG TTG GTG CGC CGC AAG AGC GCA ACT GAC CTG GGC GGG CTG CCC [13140]

#M.ap_K10 ... ... ... ... ... ... ... ... ..C ... ... ... ... ... ... [13140]

#M._ap_S397 ... ... ... ... ... ... ... ... ..C ... ... ... ... ... ... [13140]

#M._ap_JQ5 ... ... ... ... ... ... ... ... ..C ... ... ... ... ... ... [13140]

#M._ap_JQ6 ... ... ... ... ... ... ... ... ..C ... ... ... ... ... ... [13140]

#M._av_104 GGC AAG CTC GCC GAC TGC CGC TCG ACC GAC CCG CGC AAG TCG GAA [13185]

#M.ap_K10 ... ... ... ... ... ... ..G ... ... ..T ... ... ... ... ... [13185]

#M._ap_S397 ... ... ... ... ... ... ..G ... ... ... ... ... ... ... ... [13185]

#M._ap_JQ5 ... ... ... ... ... ... ..G ... ... ... ... ... ... ... ... [13185]

#M._ap_JQ6 ... ... ... ... ... ... ..G ... ... ... ... ... ... ... ... [13185]

#M._av_104 TTG TAT GTG GTC GAG GGT GAC TCG GCC GGC GGC TCG GCG AAA AGC [13230]

#M.ap_K10 ... ... ... ... ... ... ... ... ... ... ... ... ... ... ... [13230]

#M._ap_S397 ... ... ... ... ... ... ... ... ... ... ... ... ... ... ... [13230]

#M._ap_JQ5 ... ... ... ... ... ... ... ... ... ... ... ... ... ... ... [13230]

#M._ap_JQ6 ... ... ... ... ... ... ... ... ... ... ... ... ... ... ... [13230]

#M._av_104 GGC CGG GAC TCG ATG TTC CAG GCC ATC CTT CCG CTG CGC GGC AAG [13275]

#M.ap_K10 ... ... ... ... ... ... ... ... ... ... ... ... ... ... ... [13275]

#M._ap_S397 ... ... ... ... ... ... ... ... ... ... ... ... ... ... ... [13275]

#M._ap_JQ5 ... ... ... ... ... ... ... ... ... ... ... ... ... ... ... [13275]

#M._ap_JQ6 ... ... ... ... ... ... ... ... ... ... ... ... ... ... ... [13275]

#M._av_104 ATC ATC AAC GTC GAA AAG GCC CGC ATC GAC CGG GTT CTG AAG AAC [13320]

#M.ap_K10 ... ... ... ... ... ... ... ... ... ... ... ... T.. ... ... [13320]

#M._ap_S397 ... ... ... ... ... ... ... ... ... ... ... ... T.. ... ... [13320]

#M._ap_JQ5 ... ... ... ... ... ... ... ... ... ... ... ... T.. ... ... [13320]

#M._ap_JQ6 ... ... ... ... ... ... ... ... ... ... ... ... T.. ... ... [13320]

#M._av_104 ACC GAA GTG CAG GCG ATC ATC ACC GCG CTG GGC ACC GGG ATT CAC [13365]

#M.ap_K10 ... ... ... ... ... ... ... ... ... ... ... ... ... ... ... [13365]

#M._ap_S397 ... ... ... ... ... ... ... ... ... ... ... ... ... ... ... [13365]

#M._ap_JQ5 ... ... ... ... ... ... ... ... ... ... ... ... ... ... ... [13365]

#M._ap_JQ6 ... ... ... ... ... ... ... ... ... ... ... ... ... ... ... [13365]

#M._av_104 GAC GAG TTC GAC ATC ACC AAG CTG CGC TAC CAC AAG ATC GTG TTG [13410]

#M.ap_K10 ... ... ... ... ... ... ... ... ... ... ... ... ... ... ... [13410]

#M._ap_S397 ... ... ... ... ... ... ... ... ... ... ... ... ... ... ... [13410]

#M._ap_JQ5 ... ... ... ... ... ... ... ... ... ... ... ... ... ... ... [13410]

#M._ap_JQ6 ... ... ... ... ... ... ... ... ... ... ... ... ... ... ... [13410]

#M._av_104 ATG GCC GAC GCC GAC GTG GAC GGC CAG CAC ATC TCG ACG CTG TTG [13455]

#M.ap_K10 ... ... ... ... ... ... ... ... ... ... ... ... ... ... ... [13455]

#M._ap_S397 ... ... ... ... ... ... ... ... ... ... ..T ... ... ... ... [13455]

#M._ap_JQ5 ... ... ... ... ... ... ... ... ... ... ..T ... ... ... ... [13455]

#M._ap_JQ6 ... ... ... ... ... ... ... ... ... ... ..T ... ... ... ... [13455]

#M._av_104 TTG ACG CTG CTG TTC CGG TTC ATG CGG CCG CTG ATC GAA CAC GGG [13500]

#M.ap_K10 ... ... ... ... ... ... ... ... ... ... ... ... ... ... ... [13500]

#M._ap_S397 ... ... ... ... ... ... ... ... ... ... ... ... ... ... ... [13500]

#M._ap_JQ5 ... ... ... ... ... ... ... ... ... ... ... ... ... ... ... [13500]

#M._ap_JQ6 ... ... ... ... ... ... ... ... ... ... ... ... ... ... ... [13500]

#M._av_104 CAC GTG TTC TTG GCC CAG CCA CCG CTG TAC AAG CTG AAA TGG CAG [13545]

#M.ap_K10 ... ... ... ... ... ... ... ... ... ... ... ... ... ... ... [13545]

#M._ap_S397 ... ... ... ... ... ... ... ... ... ... ... ... ... ... ... [13545]

#M._ap_JQ5 ... ... ... ... ... ... ... ... ... ... ... ... ... ... ... [13545]

#M._ap_JQ6 ... ... ... ... ... ... ... ... ... ... ... ... ... ... ... [13545]

#M._av_104 CGC AGC GAT CCC GAG TTC GCC TAC TCC GAC CGC GAG CGG GAC GGG [13590]

#M.ap_K10 ... ... ... ..A ... ... ... ... ... ... ... ... ... ... ... [13590]

#M._ap_S397 ... ... ... ..A ... ... ... ... ... ... ... ... ... ... ... [13590]

#M._ap_JQ5 ... ... ... ..A ... ... ... ... ... ... ... ... ... ... ... [13590]

#M._ap_JQ6 ... ... ... ..A ... ... ... ... ... ... ... ... ... ... ... [13590]

#M._av_104 CTG CTC GAG GCC GGC CTG AAG GCC GGC AAG AAG ATC AAC AAG GAC [13635]

#M.ap_K10 ... ... ... ... ... ... ... ... ... ... ... ... ... ... ... [13635]

#M._ap_S397 ... ... ... ... ... ... ... ... ... ... ... ... ... ... ... [13635]

#M._ap_JQ5 ... ... ... ... ... ... ... ... ... ... ... ... ... ... ... [13635]

#M._ap_JQ6 ... ... ... ... ... ... ... ... ... ... ... ... ... ... ... [13635]

#M._av_104 GAC GGC ATC CAG CGC TAC AAG GGG CTG GGC GAG ATG GAC GCC AAG [13680]

#M.ap_K10 ... ..T ... ... ... ... ... ..T ... ... ... ... ... ... ... [13680]

#M._ap_S397 ... ..T ... ... ... ... ... ..T ... ... ... ... ... ... ... [13680]

#M._ap_JQ5 ... ..T ... ... ... ... ... ..T ... ... ... ... ... ... ... [13680]

#M._ap_JQ6 ... ..T ... ... ... ... ... ..T ... ... ... ... ... ... ... [13680]

#M._av_104 GAA TTG TGG GAA ACC ACA ATG GAT CCC ACC GTG CGG GTG CTG CGC [13725]

#M.ap_K10 ... ... ... ... ... ... ... ... ... ... ... ... ... ... ... [13725]

#M._ap_S397 ... ... ... ... ... ... ... ... ... ... ... ... ... ... ... [13725]

#M._ap_JQ5 ... ... ... ... ... ... ... ... ... ... ... ... ... ... ... [13725]

#M._ap_JQ6 ... ... ... ... ... ... ... ... ... ... ... ... ... ... ... [13725]

#M._av_104 CAG GTC ACG CTG GAC GAC GCC GCG GCC GCC GAC GAG CTG TTC TCC [13770]

#M.ap_K10 ... ... ... ... ... ... ... ... ... ... ... ... ... ... ... [13770]

#M._ap_S397 ... ... ... ... ... ... ... ... ... ... ... ... ... ... ... [13770]

#M._ap_JQ5 ... ... ... ... ... ... ... ... ... ... ... ... ... ... ... [13770]

#M._ap_JQ6 ... ... ... ... ... ... ... ... ... ... ... ... ... ... ... [13770]

#M._av_104 ATC CTG ATG GGC GAG GAC GTC GAC GCG CGC CGC AGC TTC ATC ACC [13815]

#M.ap_K10 ... ... ... ... ... ... ... ... ... ... ... ... ... ... ... [13815]

#M._ap_S397 ... ... ... ... ... ... ... ... ... ... ... ... ... ... ... [13815]

#M._ap_JQ5 ... ... ... ... ... ... ... ... ... ... ... ... ... ... ... [13815]

#M._ap_JQ6 ... ... ... ... ... ... ... ... ... ... ... ... ... ... ... [13815]

#M._av_104 CGC AAT GCC AAA GAC GTT CGC TTC CTA GAC GTT TAA GTG ATG CGG [13860]

#M.ap_K10 ... ... ... ... ... ... ... ... ... ... ... ... --- --- --- [13860]

#M._ap_S397 ... ... ... ... ... ... ... ... ... ... ... ... --- --- --- [13860]

#M._ap_JQ5 ... ... ... ... ... ... ... ... ... ... ... ... --- --- --- [13860]

#M._ap_JQ6 ... ... ... ... ... ... ... ... ... ... ... ... --- --- --- [13860]

#M._av_104 ATG AGC ACA GAA CCC GGT TAC GCC TCC CCC GTC GTC AAC GTC GCC [13905]

#M.ap_K10 ... ... ... ... ... ... ... ... ... ... ... ... ... ... ... [13905]

#M._ap_S397 ... ... ... ... ... ... ... ... ... ... ... ... ... ... ... [13905]

#M._ap_JQ5 ... ... ... ... ... ... ... ... ... ... ... ... ... ... ... [13905]

#M._ap_JQ6 ... ... ... ... ... ... ... ... ... ... ... ... ... ... ... [13905]

#M._av_104 TCC TCG CTG CCC CGC CGC GCC GCC GCG TCC ACC GTG CTG ATC GTG [13950]

#M.ap_K10 ... ... ... ... ... ... ... ... ... ... ... ... ... ... ... [13950]

#M._ap_S397 ... ... ... ... ... ... ... ... ... ... ... ... ... ... ... [13950]

#M._ap_JQ5 ... ... ... ... ... ... ... ... ... ... ... ... ... ... ... [13950]

#M._ap_JQ6 ... ... ... ... ... ... ... ... ... ... ... ... ... ... ... [13950]

#M._av_104 CCC GTC GTC TCC ACC GGC GAC GAC GAC AAG CCG GGC GCC GTC GTC [13995]

#M.ap_K10 ... ... ... ... ... ... ... ... ... ... ... ... ... ... ... [13995]

#M._ap_S397 ... ... ... ... ... ... ... ... ... ... ... ... ... ... ... [13995]

#M._ap_JQ5 ... ... ... ... ... ... ... ... ... ... ... ... ... ... ... [13995]

#M._ap_JQ6 ... ... ... ... ... ... ... ... ... ... ... ... ... ... ... [13995]

#M._av_104 GCG CCG GCC GGG TCG TTC CTG TCC TCC GAC GCG GTC GCC GAG ATC [14040]

#M.ap_K10 ... T.. ... ... ... ... ... ... ... ... ... ... ... ... ... [14040]

#M._ap_S397 ... T.. ... ... ... ... ... ... ... ... ... ... ... ... ... [14040]

#M._ap_JQ5 ... T.. ... ... ... ... ... ... ... ... ... ... ... ... ... [14040]

#M._ap_JQ6 ... T.. ... ... ... ... ... ... ... ... ... ... ... ... ... [14040]

#M._av_104 GAA TCC GGC CTG CGG GCG CTG GCG GCC ACC GGC GGC GCC GAA CAA [14085]

#M.ap_K10 ... ... ... ... ... ... ... ... ... ... ... ... ... ... ... [14085]

#M._ap_S397 ... ... ... ... ... ... ... ... ... ... ... ... ... ... ... [14085]

#M._ap_JQ5 ... ... ... ... ... ... ... ... ... ... ... ... ... ... ... [14085]

#M._ap_JQ6 ... ... ... ... ... ... ... ... ... ... ... ... ... ... ... [14085]

#M._av_104 CTG CAC CGG CTG GTG GTG GGA TCG CTG CCG GTG TCC AGC GTG CTG [14130]

#M.ap_K10 ... ... ... ... ... ... ... ... ... ... ... ... ... ... ... [14130]

#M._ap_S397 ... ... ... ... ... ... ... ... ... ... ... ... ... ... ... [14130]

#M._ap_JQ5 ... ... ... ... ... ... ... ... ... ... ... ... ... ... ... [14130]

#M._ap_JQ6 ... ... ... ... ... ... ... ... ... ... ... ... ... ... ... [14130]

#M._av_104 ACC GTC GGC CTG GGC AAG CCG CGA TCC GAG TGG CCG GCC GAC ACC [14175]

#M.ap_K10 ... ... ... ... ... ... ... ... ... ... ... ... ... ... ... [14175]

#M._ap_S397 ... ... ... ... ... ... ... ... ... ... ... ... ... ... ... [14175]

#M._ap_JQ5 ... ... ... ... ... ... ... ... ... ... ... ... ... ... ... [14175]

#M._ap_JQ6 ... ... ... ... ... ... ... ... ... ... ... ... ... ... ... [14175]

#M._av_104 GTC CGC CGT GCC GCG GGG GTG GCC GCG CGG TCG CTG TCC AAC ACC [14220]

#M.ap_K10 ... ... ... ... ... ... ... ... ... ... ... ... ... ... ... [14220]

#M._ap_S397 ... ... ... ... ... ... ... ... ... ... ... ... ... ... ... [14220]

#M._ap_JQ5 ... ... ... ... ... ... ... ... ... ... ... ... ... ... ... [14220]

#M._ap_JQ6 ... ... ... ... ... ... ... ... ... ... ... ... ... ... ... [14220]

#M._av_104 GAG ACG GTG TTC ACC ACG CTG GCG GCG CTG CCC GGC GAG GGC GTG [14265]

#M.ap_K10 ... ... ... ... ... ... ... ... ... ... ... ... ... ... ... [14265]

#M._ap_S397 ... ... ... ... ... ... ... ... ... ... ... ... ... ... ... [14265]

#M._ap_JQ5 ... ... ... ... ... ... ... ... ... ... ... ... ... ... ... [14265]

#M._ap_JQ6 ... ... ... ... ... ... ... ... ... ... ... ... ... ... ... [14265]

#M._av_104 GCC TCG GCC GCC GTC GAG GGC CTG ATC CTG GGC AGC TAC CGG TTC [14310]

#M.ap_K10 ... ... ... ... ... ... ... ... ... ... ... ... ... ... ... [14310]

#M._ap_S397 ... ... ... ... ... ... ... ... ... ... ... ... ... ... ... [14310]

#M._ap_JQ5 ... ... ... ... ... ... ... ... ... ... ... ... ... ... ... [14310]

#M._ap_JQ6 ... ... ... ... ... ... ... ... ... ... ... ... ... ... ... [14310]

#M._av_104 ACC GAA TTC CGC AGC GCC AAG ACC GCC CCG AAA GAC AAA GGG CTG [14355]

#M.ap_K10 ... ... ... ... ... ... ... ... ... ... ... ... ... ... ... [14355]

#M._ap_S397 ... ... ... ... ... ... ... ... ... ... ... ... ... ... ... [14355]

#M._ap_JQ5 ... ... ... ... ... ... ... ... ... ... ... ... ... ... ... [14355]

#M._ap_JQ6 ... ... ... ... ... ... ... ... ... ... ... ... ... ... ... [14355]

#M._av_104 CAA AAG ATC ACG GTG CTG GCC ACC GCC AAG GAC GCC AAG CAG GAG [14400]

#M.ap_K10 ... ... ... ... ... ... ... ... ... ... ... ... ... ... ... [14400]

#M._ap_S397 ... ... ... ... ... ... ... ... ... ... ... ... ... ... ... [14400]

#M._ap_JQ5 ... ... ... ... ... ... ... ... ... ... ... ... ... ... ... [14400]

#M._ap_JQ6 ... ... ... ... ... ... ... ... ... ... ... ... ... ... ... [14400]

#M._av_104 GTC GCG CAC GGT GCG GCC GTC GCG ACC GCG GTC GCC ACC GCC CGC [14445]

#M.ap_K10 ... ... ... ... ... ... ... ... ... ... ... ... ... ... ... [14445]

#M._ap_S397 ... ... ... ... ... ... ... ... ... ... ... ... ... ... ... [14445]

#M._ap_JQ5 ... ... ... ... ... ... ... ... ... ... ... ... ... ... ... [14445]

#M._ap_JQ6 ... ... ... ... ... ... ... ... ... ... ... ... ... ... ... [14445]

#M._av_104 GAT CTG GTC AAC ACC CCG CCC AGC CAC CTG TTT CCC GAC GAA TTC [14490]

#M.ap_K10 ... ... ... ... ... ... ... ..T ... ... ... ... ... ... ... [14490]

#M._ap_S397 ... ... ... ... ... ... ... ..T ... ... ... ... ... ... ... [14490]

#M._ap_JQ5 ... ... ... ... ... ... ... ..T ... ... ... ... ... ... ... [14490]

#M._ap_JQ6 ... ... ... ... ... ... ... ..T ... ... ... ... ... ... ... [14490]

#M._av_104 GCC AAG CGG GCA AGG GCT TTG GGT GAG TCC GTC GGC CTC GAG GTG [14535]

#M.ap_K10 ... ... ... ... ... ... ... ... ... ... ... ... ... ... ... [14535]

#M._ap_S397 ... ... ... ... ... ... ... ... ... ... ... ... ... ... ... [14535]

#M._ap_JQ5 ... ... ... ... ... ... ... ... ... ... ... ... ... ... ... [14535]

#M._ap_JQ6 ... ... ... ... ... ... ... ... ... ... ... ... ... ... ... [14535]

#M._av_104 GAG GTG ATC GAC GAG AAG GCG CTG CAA AAG GGC GGC TAC GGA GGG [14580]

#M.ap_K10 ... ... C.. ... ... ... ... ... ... ... ... ... ... ... ... [14580]

#M._ap_S397 ... ... C.. ... ... ... ... ... ... ... ... ... ... ... ... [14580]

#M._ap_JQ5 ... ... C.. ... ... ... ... ... ... ... ... ... ... ... ... [14580]

#M._ap_JQ6 ... ... C.. ... ... ... ... ... ... ... ... ... ... ... ... [14580]

#M._av_104 ATT CTG GGC GTC GGC CAG GGT TCG TCG CGC CCG CCG CGG CTG GTG [14625]

#M.ap_K10 ... ... ... ... ... ... ... ... ... ... ... ... ... ... ... [14625]

#M._ap_S397 ... ... ... ... ... ... ... ... ... ... ... ... ... ... ... [14625]

#M._ap_JQ5 ... ... ... ... ... ... ... ... ... ... ... ... ... ... ... [14625]

#M._ap_JQ6 ... ... ... ... ... ... ... ... ... ... ... ... ... ... ... [14625]

#M._av_104 CGG CTG ATC CAC CGC GGT TCG CGG CTG GCC AAG AAA TCC AAG CAG [14670]

#M.ap_K10 ... ... ... ... ... ... ... ... ... ... ... ... ... ... ... [14670]

#M._ap_S397 ... ... ... ... ... ... ... ... ... ... ... ... ... ... ... [14670]

#M._ap_JQ5 ... ... ... ... ... ... ... ... ... ... ... ... ... ... ... [14670]

#M._ap_JQ6 ... ... ... ... ... ... ... ... ... ... ... ... ... ... ... [14670]

#M._av_104 GCG AAA AAA GTG GCG CTA GTC GGC AAG GGT GTC ACC TTC GAC ACC [14715]

#M.ap_K10 ... ... ..G ... ... ..G ... ... ... ..C ... ... ... ... ... [14715]

#M._ap_S397 ... ... ..G ... ... ..G ... ... ... ..C ... ... ... ... ... [14715]

#M._ap_JQ5 ... ... ..G ... ... ..G ... ... ... ..C ... ... ... ... ... [14715]

#M._ap_JQ6 ... ... ..G ... ... ..G ... ... ... ..C ... ... ... ... ... [14715]

#M._av_104 GGC GGC ATC TCG ATC AAG CCG GCG GCC TCG ATG CAT CAC ATG ACC [14760]

#M.ap_K10 ... ... ... ... ... ... ... ... ... ... ... ... ... ... ... [14760]

#M._ap_S397 ... ... ... ... ... ... ... ... ... ... ... ... ... ... ... [14760]

#M._ap_JQ5 ... ... ... ... ... ... ... ... ... ... ... ... ... ... ... [14760]

#M._ap_JQ6 ... ... ... ... ... ... ... ... ... ... ... ... ... ... ... [14760]

#M._av_104 TCG GAC ATG GGC GGG GCC GCC GCC GTC ATC GCC ACC GTG GCG CTG [14805]

#M.ap_K10 ... ... ... ... ... ... ... ... ... ... ... ... ... ... ... [14805]

#M._ap_S397 ... ... ... ... ... ... ... ... ... ... ... ... ... ... ... [14805]

#M._ap_JQ5 ... ... ... ... ... ... ... ... ... ... ... ... ... ... ... [14805]

#M._ap_JQ6 ... ... ... ... ... ... ... ... ... ... ... ... ... ... ... [14805]

#M._av_104 GCC GCG CGG CTG CAG CTG CCG ATC GAC GTG ATC GCC ACC GTG CCG [14850]

#M.ap_K10 ... ... .A. ..T ... ... ... ... ... ... ... ... ... ... ... [14850]

#M._ap_S397 ... ... .A. ..T ... ... ... ... ... ... ... ... ... ... ... [14850]

#M._ap_JQ5 ... ... .A. ..T ... ... ... ... ... ... ... ... ... ... ... [14850]

#M._ap_JQ6 ... ... .A. ..T ... ... ... ... ... ... ... ... ... ... ... [14850]

#M._av_104 ATG GCG GAG AAC ATG CCG TCG GGC ACC GCG CAG CGG CCC GGC GAC [14895]

#M.ap_K10 ... ... ... ... ... ... ... ... ... ... ... ... ... ... ..T [14895]

#M._ap_S397 ... ... ... ... ... ... ... ... ... ... ... ... ... ... ..T [14895]

#M._ap_JQ5 ... ... ... ... ... ... ... ... ... ... ... ... ... ... ..T [14895]

#M._ap_JQ6 ... ... ... ... ... ... ... ... ... ... ... ... ... ... ..T [14895]

#M._av_104 GTG CTG ACG CAG TAC GGC GGC ACC ACC GTC GAG GTG CAG AAC ACC [14940]

#M.ap_K10 ... ... ... ... ... ... ... ... ... ... ... ... ... ... ... [14940]

#M._ap_S397 ... ... ... ... ... ... ... ... ... ... ... ... ... ... ... [14940]

#M._ap_JQ5 ... ... ... ... ... ... ... ... ... ... ... ... ... ... ... [14940]

#M._ap_JQ6 ... ... ... ... ... ... ... ... ... ... ... ... ... ... ... [14940]

#M._av_104 GAC GCC GAG GGC CGG CTG ATC CTG GCC GAC GCC ATC GTG CGG GCC [14985]

#M.ap_K10 ... ... ... ... ... ... ..T ... ... ... ... ... ... ... ... [14985]

#M._ap_S397 ... ... ... ... ... ... ..T ... ... ... ... ... ... ... ... [14985]

#M._ap_JQ5 ... ... ... ... ... ... ..T ... ... ... ... ... ... ... ... [14985]

#M._ap_JQ6 ... ... ... ... ... ... ..T ... ... ... ... ... ... ... ... [14985]

#M._av_104 TGC GAG GAC AAC CCG GAC TAC CTG ATC GAG ACC TCC ACG CTG ACC [15030]

#M.ap_K10 ... ... ... ... ... ... ... ... ... ... ... ... ... ... ... [15030]

#M._ap_S397 ... ... ... ... ... ... ... ... ... ... ... ... ... ... ... [15030]

#M._ap_JQ5 ... ... ... ... ... ... ... ... ... ... ... ... ... ... ... [15030]

#M._ap_JQ6 ... ... ... ... ... ... ... ... ... ... ... ... ... ... ... [15030]

#M._av_104 GGC GCG CAG ACC GTC GCG CTG GGC GCC CGG ATC CCC GGC GTG ATG [15075]

#M.ap_K10 ... ... ... ... ... ... ... ... ... ... ... ... ... ... ... [15075]

#M._ap_S397 ... ... ... ... ... ... ... ... ... ... ... ... ... ... ... [15075]

#M._ap_JQ5 ... ... ... ... ... ... ... ... ... ... ... ... ... ... ... [15075]

#M._ap_JQ6 ... ... ... ... ... ... ... ... ... ... ... ... ... ... ... [15075]

#M._av_104 GGC AGC GAC GAG TTC CGC GAC CGG GTC GCG GCG ATC TCG CAG CGG [15120]

#M.ap_K10 ... ... ... ... ... ... ... ... ..G ... ... ... ... ... ... [15120]

#M._ap_S397 ... ... ... ... ... ... ... ... ..G ... ... ... ... ... ... [15120]

#M._ap_JQ5 ... ... ... ... ... ... ... ... ..G ... ... ... ... ... ... [15120]

#M._ap_JQ6 ... ... ... ... ... ... ... ... ..G ... ... ... ... ... ... [15120]

#M._av_104 GTC GGT GAG AAC GGC TGG CCG ATG CCG CTG CCC GAC GAG CTC AAG [15165]

#M.ap_K10 ... ... ... ... ... ... ... ... ... ... ... ... ... ... ... [15165]

#M._ap_S397 ... ... ... ... ... ... ... ... ... ... ... ... ... ... ... [15165]

#M._ap_JQ5 ... ... ... ... ... ... ... ... ... ... ... ... ... ... ... [15165]

#M._ap_JQ6 ... ... ... ... ... ... ... ... ... ... ... ... ... ... ... [15165]

#M._av_104 GAG GAC CTG AAG TCC ACG GTG GCC GAC CTG TCG AAC ATC AGC GGG [15210]

#M.ap_K10 ... ... ... ... ... ... ... ... ... ... ... ... ... ... ... [15210]

#M._ap_S397 ... ... ... ... ... ... ... ... ... ... ... ... ... ... ... [15210]

#M._ap_JQ5 ... ... ... ... ... ... ... ... ... ... ... ... ... ... ... [15210]

#M._ap_JQ6 ... ... ... ... ... ... ... ... ... ... ... ... ... ... ... [15210]

#M._av_104 CAG CGC TTC GCC GGC ATG CTG GTG GCC GGC GTG TTC CTG CGC GAG [15255]

#M.ap_K10 ... ... ... ... ... ... ... ... ... ... ... ... ... ... ... [15255]

#M._ap_S397 ... ... ... ... ... ... ... ... ... ... ... ... ... ... ... [15255]

#M._ap_JQ5 ... ... ... ... ... ... ... ... ... ... ... ... ... ... ... [15255]

#M._ap_JQ6 ... ... ... ... ... ... ... ... ... ... ... ... ... ... ... [15255]

#M._av_104 TTC GTC GCC GAC GGG GTG GGC TGG GCG CAC ATC GAC GTG GCC GGC [15300]

#M.ap_K10 ... A.. ... ... ... ... ... ... ... ... ... ... ... ... ... [15300]

#M._ap_S397 ... A.. ... ... ... ... ... ... ... ... ... ... ... ... ... [15300]

#M._ap_JQ5 ... A.. ... ... ... ... ... ... ... ... ... ... ... ... ... [15300]

#M._ap_JQ6 ... A.. ... ... ... ... ... ... ... ... ... ... ... ... ... [15300]

#M._av_104 CCG GCC TAC AAC ACC GGC AGC CCG TGG GGT TAT TCG CCC AAG GGC [15345]

#M.ap_K10 ... ... ... ... ... ... ... ... ... ... ... ... ... ..A ... [15345]

#M._ap_S397 ... ... ... ... ... ... ... ... ... ... ... ... ... ..A ... [15345]

#M._ap_JQ5 ... ... ... ... ... ... ... ... ... ... ... ... ... ..A ... [15345]

#M._ap_JQ6 ... ... ... ... ... ... ... ... ... ... ... ... ... ..A ... [15345]

#M._av_104 GCC ACC GGG GTG CCG ACG CGC ACC ATG TTC GCG GTG CTC GAA GAC [15390]

#M.ap_K10 ... ... ... ... ... ... ... ... ... ... ..A ... ... ... ... [15390]#M._ap_S397 ... ... ... ... ... ... ... ... ... ... ..A ... ... ... ... [15390]

#M._ap_JQ5 ... ... ... ... ... ... ... ... ... ... ..A ... ... ... ... [15390]

#M._ap_JQ6 ... ... ... ... ... ... ... ... ... ... ..A ... ... ... ... [15390]

#M._av_104 ATC GCC GCG AAC GGC TAA GTG TAC GTC CGG CAT TTA GGA CTG CGC [15435]

#M.ap_K10 ... ... ... ... ... ... ... ... ... ... ... ... ... ... ... [15435]

#M._ap_S397 ... ... ... ... ... ... ... ... ... ... ... ... ... ... ... [15435]

#M._ap_JQ5 ... ... ... ... ... ... ... ... ... ... ... ... ... ... ... [15435]

#M._ap_JQ6 ... ... ... ... ... ... ... ... ... ... ... ... ... ... ... [15435]

#M._av_104 GAC TTC CGG TCC TGG GCA CAC GCC GAC CTC GAA CTG CAG CCG GGT [15480]

#M.ap_K10 ... ... ... ... ... ... ... ... ... ... ... ... ... ... ... [15480]

#M._ap_S397 ... ... ... ... ... ... ... ... ... ... ... ... ... ... ... [15480]

#M._ap_JQ5 ... ... ... ... ... ... ... ... ... ... ... ... ... ... ... [15480]

#M._ap_JQ6 ... ... ... ... ... ... ... ... ... ... ... ... ... ... ... [15480]

#M._av_104 CGG ACG GTC TTC ATC GGG TCC AAC GGC TTC GGG AAG ACG AAT CTG [15525]

#M.ap_K10 ... ... ... ... ... ... ... ... ... ... ... ... ... ... ... [15525]

#M._ap_S397 ... ... ... ... ... ... ... ... ... ... ... ... ... ... ... [15525]

#M._ap_JQ5 ... ... ... ... ... ... ... ... ... ... ... ... ... ... ... [15525]

#M._ap_JQ6 ... ... ... ... ... ... ... ... ... ... ... ... ... ... ... [15525]

#M._av_104 CTT GAG GCG CTG TGG TAT TCG AGC ACG CTG GGG TCA CAC CGG GTG [15570]

#M.ap_K10 ... ... ... ... ... ... ... ... ... ... ... ... ... ... ... [15570]

#M._ap_S397 ... ... ... ... ... ... ... ... ... ... ... ... ... ... ... [15570]

#M._ap_JQ5 ... ... ... ... ... ... ... ... ... ... ... ... ... ... ... [15570]

#M._ap_JQ6 ... ... ... ... ... ... ... ... ... ... ... ... ... ... ... [15570]

#M._av_104 GGC ACG GAC GCG CCG TTG ATC CGC GCC GGC GCC GAC CGG ACC GTG [15615]

#M.ap_K10 ... ... ... ... ... ... ... ... ... ... ... ... ... G.G ... [15615]

#M._ap_S397 ... ... ... ... ... ... ... ... ... ... ... ... ... G.G ... [15615]

#M._ap_JQ5 ... ... ... ... ... ... ... ... ... ... ... ... ... G.G ... [15615]

#M._ap_JQ6 ... ... ... ... ... ... ... ... ... ... ... ... ... G.G ... [15615]

#M._av_104 GTG TCG ACC ATC GTG GTC AAC GAC GGC CGG GAA TGC GCG GTC GAT [15660]

#M.ap_K10 ... ... ... ... ... ... ... ... ... ... ... ..T ... ... ... [15660]

#M._ap_S397 ... ... ... ... ... ... ... ... ... ... ... ..T ... ... ... [15660]

#M._ap_JQ5 ... ... ... ... ... ... ... ... ... ... ... ..T ... ... ... [15660]

#M._ap_JQ6 ... ... ... ... ... ... ... ... ... ... ... ..T ... ... ... [15660]

#M._av_104 CTG GAG ATC GCC GCC GGC CGG GCG AAC AAG GCG CGG CTG AAC CGG [15705]

#M.ap_K10 ... ... ... ... ... ... ... ... ... ... ... ... ... ... ... [15705]

#M._ap_S397 ... ... ... ... ... ... ... ... ... ... ... ... ... ... ... [15705]

#M._ap_JQ5 ... ... ... ... ... ... ... ... ... ... ... ... ... ... ... [15705]

#M._ap_JQ6 ... ... ... ... ... ... ... ... ... ... ... ... ... ... ... [15705]

#M._av_104 TCA CCC GTG CGC AGC ACC CGC GAG GTA CTC GGC GTG CTG CGC GCG [15750]

#M.ap_K10 ... ... ... ... ... ... ... ... ..G ... ... ... ... ... ... [15750]

#M._ap_S397 .T. ... ... ... ... ... ... ... ..G ... ... ... ... ... ... [15750]

#M._ap_JQ5 .T. ... ... ... ... ... ... ... ..G ... ... ... ... ... ... [15750]

#M._ap_JQ6 .T. ... ... ... ... ... ... ... ..G ... ... ... ... ... ... [15750]

#M._av_104 GTG CTG TTC GCC CCC GAG GAC CTG GCC CTG GTG CGC GGG GAT CCC [15795]

#M.ap_K10 ... ... ... ... ... ... ... ... ... ... ... ... ... ... ... [15795]

#M._ap_S397 ... ... ... ... ... ... ... ... ... ... ... ... ... ... ... [15795]

#M._ap_JQ5 ... ... ... ... ... ... ... ... ... ... ... ... ... ... ... [15795]

#M._ap_JQ6 ... ... ... ... ... ... ... ... ... ... ... ... ... ... ... [15795]

#M._av_104 TCC GAG CGG CGC CGC TAC CTC GAC GAC CTG GCG ACG CTG CGG CGC [15840]

#M.ap_K10 ... ... ... ... ..T ... ... ... ... ... ... ... ... ... ... [15840]

#M._ap_S397 ... ... ... ... ..T ... ... ... ... ... ... ... ... ... ... [15840]

#M._ap_JQ5 ... ... ... ... ..T ... ... ... ... ... ... ... ... ... ... [15840]

#M._ap_JQ6 ... ... ... ... ..T ... ... ... ... ... ... ... ... ... ... [15840]

#M._av_104 CCG GCG ATC GCC GCG GTG CGC GCC GAC TAC GAC AAG GTG TTG CGG [15885]

#M.ap_K10 ... ... ... ... ... ... ... ... ... ... ... ... ... ... ... [15885]

#M._ap_S397 ... ... ... ... ... ... ... ... ... ... ... ... ... ... ... [15885]

#M._ap_JQ5 ... ... ... ... ... ... ... ... ... ... ... ... ... ... ... [15885]

#M._ap_JQ6 ... ... ... ... ... ... ... ... ... ... ... ... ... ... ... [15885]

#M._av_104 CAG CGC ACC GCG TTG CTC AAA TCG CTG TCC GGT GCC CGC CAC CGG [15930]

#M.ap_K10 ... ... ... ... ... ... ... ... ... ... ... ... ... ... ... [15930]

#M._ap_S397 ... ... ... ... ... ... ... ... ... ... ... ... ... ... ... [15930]

#M._ap_JQ5 ... ... ... ... ... ... ... ... ... ... ... ... ... ... ... [15930]

#M._ap_JQ6 ... ... ... ... ... ... ... ... ... ... ... ... ... ... ... [15930]

#M._av_104 GGC GAC CGC GGC GCG CTG GAC ACC CTC GAC GTG TGG GAC AGC CGG [15975]

#M.ap_K10 ... ... ... ... ..T ... ... ... ... ... ... ... ... ... ... [15975]

#M._ap_S397 ... ... ... ... ..T ... ... ... ... ... ... ... ... ... ... [15975]

#M._ap_JQ5 ... ... ... ... ..T ... ... ... ... ... ... ... ... ... ... [15975]

#M._ap_JQ6 ... ... ... ... ..T ... ... ... ... ... ... ... ... ... ... [15975]

#M._av_104 CTG GCC GAA TAC GGG GCC CAA TTG ATG GCT GCC CGA ATC GAT TTG [16020]

#M.ap_K10 ... ... ... ... ... ... ... ... ... ... ... ... ... ... ... [16020]

#M._ap_S397 ... ... ... ... ... ... ... ... ... ... ... ... ... ... ... [16020]

#M._ap_JQ5 ... ... ... ... ... ... ... ... ... ... ... ... ... ... ... [16020]

#M._ap_JQ6 ... ... ... ... ... ... ... ... ... ... ... ... ... ... ... [16020]

#M._av_104 GTG AAC CAG CTG GCG CCG GAG GTC GAG AAG GCC TAT CAG CTG CTG [16065]

#M.ap_K10 ... ... ... T.. ... ... ... ..G ... ... ... ... ... ... ... [16065]

#M._ap_S397 ... ... ... T.. ... ... ... ..G ... ... ... ... ... ... ... [16065]

#M._ap_JQ5 ... ... ... T.. ... ... ... ..G ... ... ... ... ... ... ... [16065]

#M._ap_JQ6 ... ... ... T.. ... ... ... ..G ... ... ... ... ... ... ... [16065]

#M._av_104 GCC CCG GGA TCG CGG GCG GCA TCG ATC GGC TAC CGG TCC AGC CTG [16110]

#M.ap_K10 ... ... ... ... ... ... ..G ... ... ... ... ..A ... ... ... [16110]

#M._ap_S397 ... ... ... ... ... ... ..G ... ... ... ... ..A ... ... ... [16110]

#M._ap_JQ5 ... ... ... ... ... ... ..G ... ... ... ... ..A ... ... ... [16110]

#M._ap_JQ6 ... ... ... ... ... ... ..G ... ... ... ... ..A ... ... ... [16110]

#M._av_104 GGC GCG GCG GCC GCG GCC GAG GTG AAC GCC GGC GAC CGC GAC TAT [16155]

#M.ap_K10 ... ... ... ... T.. ... ... ... ... ... ... ... ... ... ... [16155]

#M._ap_S397 ... ... ... ... T.. ... ... ... ... ... ... ... ... ... ... [16155]

#M._ap_JQ5 ... ... ... ... T.. ... ... ... ... ... ... ... ... ... ... [16155]

#M._ap_JQ6 ... ... ... ... T.. ... ... ... ... ... ... ... ... ... ... [16155]

#M._av_104 CTG GAG GCC GCG CTG CTG GCC GGG TTG GCG GCC CGC CGG TAC GCC [16200]

#M.ap_K10 ... ... ... ... ... ... ... ... ... ... ... .A. ... G.. ... [16200]

#M._ap_S397 ... ... ... ... ... ... ... ... ... ... ... ... ... G.. ... [16200]

#M._ap_JQ5 ... ... ... ... ... ... ... ... ... ... ... ... ... G.. ... [16200]

#M._ap_JQ6 ... ... ... ... ... ... ... ... ... ... ... ... ... G.. ... [16200]

#M._av_104 GAA CTG GAA CGG GGC GTG TGC CTG GTC GGC CCG CAC CGC GAC GAC [16245]

#M.ap_K10 ... ... ... ... ... A.. ... ... ... ... ... ... ... ... ... [16245]

#M._ap_S397 ... ... ... ... ... A.. ... ... ... ... ... ... ... ... ... [16245]

#M._ap_JQ5 ... ... ... ... ... A.. ... ... ... ... ... ... ... ... ... [16245]

#M._ap_JQ6 ... ... ... ... ... A.. ... ... ... ... ... ... ... ... ... [16245]

#M._av_104 CTG GAG CTG TGG CTC GGT GAG CAG GTG GCG AAA GGC TTT GCC AGC [16290]

#M.ap_K10 ... ... ... ... ... ... ... ... ... ... ... ... ... ... ... [16290]

#M._ap_S397 ... ... ... ... ... ... ... ... ... ... ... ... ... ... ... [16290]

#M._ap_JQ5 ... ... ... ... ... ... ... ... ... ... ... ... ... ... ... [16290]

#M._ap_JQ6 ... ... ... ... ... ... ... ... ... ... ... ... ... ... ... [16290]

#M._av_104 CAT GGG GAA TCG TGG TCG CTG GCG CTG TCC CTT CGG CTC GCC GCC [16335]

#M.ap_K10 ... ... ... ... ... ... ... ... ... ... ..G ... ..G ... ... [16335]

#M._ap_S397 ... ... ... ... ... ... ... ... ... ... ..G ... ..G ... ... [16335]

#M._ap_JQ5 ... ... ... ... ... ... ... ... ... ... ..G ... ..G ... ... [16335]

#M._ap_JQ6 ... ... ... ... ... ... ... ... ... ... ..G ... ..G ... ... [16335]

#M._av_104 TAC GAG TTG TTG CGG GCC GAC GAA AGC GAT CCG GTG TTG CTG CTC [16380]

#M.ap_K10 .T. ... ... C.. ... ... ... ... ... ... ... ... ... ... ... [16380]

#M._ap_S397 .T. ... ... C.. ... ... ... ... ... ... ... ... ... ... ... [16380]

#M._ap_JQ5 .T. ... ... C.. ... ... ... ... ... ... ... ... ... ... ... [16380]

#M._ap_JQ6 .T. ... ... C.. ... ... ... ... ... ... ... ... ... ... ... [16380]

#M._av_104 GAC GAC GTG TTC GCC GAG CTG GAC GCC GCC CGC CGC CGG GCA CTG [16425]

#M.ap_K10 ... ... ... ... ... ... ..C ... ... ... ... ... ... ... ... [16425]

#M._ap_S397 ... ... ... ... ... ... ..C ... ... ... ... ... ... ... ... [16425]

#M._ap_JQ5 ... ... ... ... ... ... ..C ... ... ... ... ... ... ... ... [16425]

#M._ap_JQ6 ... ... ... ... ... ... ..C ... ... ... ... ... ... ... ... [16425]

#M._av_104 GCC GCC GTG GCC GAA TCC GCC GAG CAG GTG TTG GTC ACC GCG GCG [16470]

#M.ap_K10 ... ..G ... ... ... ... ... ..A ... ... ... ... ... ... ... [16470]

#M._ap_S397 ... ..G ... ... ... ... ... ..A ... ... ... ... ... ... ... [16470]

#M._ap_JQ5 ... ..G ... ... ... ... ... ..A ... ... ... ... ... ... ... [16470]

#M._ap_JQ6 ... ..G ... ... ... ... ... ..A ... ... ... ... ... ... ... [16470]

#M._av_104 GTG CTC GAA GAC ATC CCG GCG GGC TGG CAG GCT CGG CGG CTC TTC [16515]

#M.ap_K10 ... ... ... ... ... ... A.. ... ... ... ... ... ... ... ... [16515]

#M._ap_S397 ... ... ... ... ... ... A.. ... ... ... ... ... ... ... ... [16515]

#M._ap_JQ5 ... ... ... ... ... ... A.. ... ... ... ... ... ... ... ... [16515]

#M._ap_JQ6 ... ... ... ... ... ... A.. ... ... ... ... ... ... ... ... [16515]

#M._av_104 GTC GAG TTG CGC GAC ACC GAC GCG GGC CGG GTA TCG GAG CTG CGC [16560]

#M.ap_K10 ... ... ... ... ... ... ... ... ... ... ... ... ... ... ... [16560]

#M._ap_S397 ... ... ... ... ... ... ... ... ... ... ... ... ... ... ... [16560]

#M._ap_JQ5 ... ... ... ... ... ... ... ... ... ... ... ... ... ... ... [16560]

#M._ap_JQ6 ... ... ... ... ... ... ... ... ... ... ... ... ... ... ... [16560]

#M._av_104 CCA TGA GTG GCT GAA TAC ACC CTG CCC GAC CTG GAC TGG GAC TAT [16605]

#M.ap_K10 ... ... ... ... ... ... ... ... ... ... ... ... ... ... ... [16605]

#M._ap_S397 ... ... ... ... ... ... ... ... ... ... ... ... ... ... ... [16605]

#M._ap_JQ5 ... ... ... ... ... ... ... ... ... ... ... ... ... ... ... [16605]

#M._ap_JQ6 ... ... ... ... ... ... ... ... ... ... ... ... ... ... ... [16605]

#M._av_104 GCA GCG TTG GAA CCG CAC ATC TCG GGG CAG ATC AAC GAG ATC CAC [16650]

#M.ap_K10 ... ... ... ... ... ... ... ... ... ... ... ... ... ... ... [16650]

#M._ap_S397 ... ... ... ... ... ... ... ... ... ... ... ... ... ... ... [16650]

#M._ap_JQ5 ... ... ... ... ... ... ... ... ... ... ... ... ... ... ... [16650]

#M._ap_JQ6 ... ... ... ... ... ... ... ... ... ... ... ... ... ... ... [16650]

#M._av_104 CAC ACC AAG CAC CAC GCC ACC TAC GTC AAA GGC GTG AAC GAC GCT [16695]

#M.ap_K10 ... ... ... ... ... ... ... ... ... ... ... ... ... ... ... [16695]

#M._ap_S397 ... ... ... ... ... ... ... ... ... ... ... ... ... ... ... [16695]

#M._ap_JQ5 ... ... ... ... ... ... ... ... ... ... ... ... ... ... ... [16695]

#M._ap_JQ6 ... ... ... ... ... ... ... ... ... ... ... ... ... ... ... [16695]

#M._av_104 CTT GCC AAG CTC GAA GAG GCC CGC GCC AAC GAG GAC CAC GCT GCG [16740]

#M.ap_K10 ... ... ... ... ... ... ... ... ... ... ... ... ... ... ... [16740]

#M._ap_S397 ... ... ... ... ... ... ... ... ... ... ... ... ... ... ... [16740]

#M._ap_JQ5 ... ... ... ... ... ... ... ... ... ... ... ... ... ... ... [16740]

#M._ap_JQ6 ... ... ... ... ... ... ... ... ... ... ... ... ... ... ... [16740]

#M._av_104 ATC TTC CTG AAC GAA AAG AAC CTC GCC TTC CAC CTG GGC GGC CAC [16785]

#M.ap_K10 ... ... ... ... ... ... ... ... ... ... ... ... ... ... ... [16785]

#M._ap_S397 ... ... ... ... ... ... ... ... ... ... ... ... ... ... ... [16785]

#M._ap_JQ5 ... ... ... ... ... ... ... ... ... ... ... ... ... ... ... [16785]

#M._ap_JQ6 ... ... ... ... ... ... ... ... ... ... ... ... ... ... ... [16785]

#M._av_104 GTC AAC CAC TCG ATC TGG TGG AAG AAC CTG TCG CCG GAC GGC GGT [16830]

#M.ap_K10 ... ... ... ... ... ... ... ... ... ... ... ... ... ... ... [16830]

#M._ap_S397 ... ... ... ... ... ... ... ... ... ... ... ... ... ... ... [16830]

#M._ap_JQ5 ... ... ... ... ... ... ... ... ... ... ... ... ... ... ... [16830]

#M._ap_JQ6 ... ... ... ... ... ... ... ... ... ... ... ... ... ... ... [16830]

#M._av_104 GAC AAG CCC ACC GGT GAG CTG GCC GCC GCG ATC GAC GAC GCG TTC [16875]

#M.ap_K10 ... ... ... ... ..C ... ... ... ... ... ... ... ... ... ... [16875]

#M._ap_S397 ... ... ... ... ..C ... ... ... ... ... ... ... ... ... ... [16875]

#M._ap_JQ5 ... ... ... ... ..C ... ... ... ... ... ... ... ... ... ... [16875]

#M._ap_JQ6 ... ... ... ... ..C ... ... ... ... ... ... ... ... ... ... [16875]

#M._av_104 GGG TCC TTC GAC AAG TTC CGA GCG CAA TTC AGC GCC GCC GCC AAC [16920]

#M.ap_K10 ..C ... ... ... ... ... ..G ... ... ... ... ... ... ... ... [16920]

#M._ap_S397 ..C ... ... ... ... ... ..G ... ... ... ... ... ... ... ... [16920]

#M._ap_JQ5 ..C ... ... ... ... ... ..G ... ... ... ... ... ... ... ... [16920]

#M._ap_JQ6 ..C ... ... ... ... ... ..G ... ... ... ... ... ... ... ... [16920]

#M._av_104 GGC CTG CAG GGC TCC GGC TGG GCG GTG CTG GGC TAT GAC ACC CTG [16965]

#M.ap_K10 ... ... ... ... ... ... ... ... ... ... ..T ... ... ... G.. [16965]

#M._ap_S397 ... ... ... ... ... ... ... ... ... ... ..T ... ... ... ... [16965]

#M._ap_JQ5 ... ... ... ... ... ... ... ... ... ... ..T ... ... ... ... [16965]

#M._ap_JQ6 ... ... ... ... ... ... ... ... ... ... ..T ... ... ... ... [16965]

#M._av_104 GGC AGC CGG TTG CTG ACC TTC CAG CTC TAC GAC CAG CAG GCC AAC [17010]

#M.ap_K10 ... ... ... ... ... ... ... ... ... ... ... ... ... ... ... [17010]

#M._ap_S397 ... ... ... ... ... ... ... ... ... ... ... ... ... ... ... [17010]

#M._ap_JQ5 ... ... ... ... ... ... ... ... ... ... ... ... ... ... ... [17010]

#M._ap_JQ6 ... ... ... ... ... ... ... ... ... ... ... ... ... ... ... [17010]

#M._av_104 GTC CCG CTG GGC ATC ATC CCG CTG CTG CAG GTC GAC ATG TGG GAG [17055]

#M.ap_K10 ... ... ... ... ... ... ... ... ... ... ... ... ... ... ... [17055]

#M._ap_S397 ... ... ... ... ... ... ... ... ... ... ... ... ... ... ... [17055]

#M._ap_JQ5 ... ... ... ... ... ... ... ... ... ... ... ... ... ... ... [17055]

#M._ap_JQ6 ... ... ... ... ... ... ... ... ... ... ... ... ... ... ... [17055]

#M._av_104 CAC GCG TTC TAC CTG CAG TAC AAG AAC GTC AAG GCG GAC TAC GTC [17100]

#M.ap_K10 ... ... ... ... ... ... ... ... ... ... ... ... ..T ... ... [17100]

#M._ap_S397 ... ... ... ... ... ... ... ... ... ... ... ... ..T ... ... [17100]

#M._ap_JQ5 ... ... ... ... ... ... ... ... ... ... ... ... ..T ... ... [17100]

#M._ap_JQ6 ... ... ... ... ... ... ... ... ... ... ... ... ..T ... ... [17100]

#M._av_104 AAG GCG TTC TGG AAC GTG GTC AAC TGG GCG GAC GTG CAG AAG CGG [17145]

#M.ap_K10 ... ... ... ... ... ... ... ... ... ... ... ... ... ... ... [17145]

#M._ap_S397 ... ... ... ... ... ... ... ... ... ... ... ... ... ... ... [17145]

#M._ap_JQ5 ... ... ... ... ... ... ... ... ... ... ... ... ... ... ... [17145]

#M._ap_JQ6 ... ... ... ... ... ... ... ... ... ... ... ... ... ... ... [17145]

#M._av_104 TAC GCC GCC GCC ACT TCC AAG GCC CAA GGC CTG ATC TTC GGC TGA [17190]

#M.ap_K10 ... ... ... ... ... ... ... ... ... ... ... ... ... ... ... [17190]

#M._ap_S397 ... ... ... ... ... ... ... ... ... ... ... ... ... ... ... [17190]

#M._ap_JQ5 ... ... ... ... ... ... ... ... ... ... ... ... ... ... ... [17190]

#M._ap_JQ6 ... ... ... ... ... ... ... ... ... ... ... ... ... ... ... [17190]
